# Chemoselective Alpha‐Deuteration of Amides via Retro‐ene Reaction

**DOI:** 10.1002/chem.202004103

**Published:** 2020-11-17

**Authors:** Vincent Porte, Giovanni Di Mauro, Manuel Schupp, Daniel Kaiser, Nuno Maulide

**Affiliations:** ^1^ Institute of Organic Chemistry University of Vienna Währinger Strasse 38 1090 Vienna Austria; ^2^ CeMM—Research Center for Molecular Medicine Austrian Academy of Sciences Lazarettgasse 14, AKH BT 25.3 1090 Vienna Austria

**Keywords:** amide activation, chemoselectivity, deuteration, sulfoxide isotopic labelling

## Abstract

A synthetically convenient approach for the direct α‐deuteration of amides is reported. This mechanistically unusual process relies on a retro‐ene‐type process, triggered by the addition of deuterated dimethyl sulfoxide to a keteniminium intermediate, generated through electrophilic amide activation. The transformation displays broad functional‐group tolerance and high deuterium incorporation.

## Graphical abstract

**Label me softly**: A facile method for the α‐deuteration of amides via a retro‐ene reaction was developed. The process is triggered by the addition of [D_6_]DMSO to activated amides and tolerates a broad‐range of functional groups, affording products in good yields and with high levels of deuterium incorporation throughout.

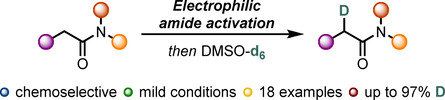

The elucidation of reaction mechanisms has long been at the heart of organic synthesis. In particular, isotope labelling is a powerful tool that enables the precise monitoring of specific atoms by using them as markers during chemical transformations.[^1^, ^2^] Moreover, understanding the fate of a drug candidate is of great importance to drug discovery, especially when studying the absorption, distribution, metabolism and excretion (ADME) properties.^[3]^ The introduction of isotopic labels is also one of the most effective and least invasive methods for monitoring bioactive substances.^[4]^ While radioactive compounds containing T or ^14^C are frequently used for quantification of metabolites and ADME properties,[^5^, ^6^] stable isotopes such as D, ^13^C, ^18^O or ^15^N can also be used as internal standards for bioassays,^[7]^ or for overcoming matrix effects from sample analysis in LC/MS studies.^[8]^ Moreover, life sciences can exploit the deuterium kinetic isotope effect to slow down cytochrome P450 metabolism, optimize pharmacokinetic properties or reduce toxicity.[^9^, ^10^, ^11^] Expanding the toolbox to enable cheap, regioselective and mild late‐stage deuterium incorporation is therefore highly desirable. A prime example of such a transformation was reported by MacMillan, who developed a photoredox‐catalyzed deuteration and tritiation of bioactive compounds using D_2_O or T_2_O,^[12]^ while more recently Wasa et al. reported a frustrated Lewis pair‐catalyzed α‐deuteration of carbonyl compounds.^[13]^ Also, late‐stage hydrogen isotope exchange, often mediated by an organometallic complex, has been a method of choice to incorporate a D or T.[^14^, ^15^]

While other methods for the α‐deuteration of carbonyls are known, many of these suffer from low selectivity, owing to the fact that they are strongly pK_a_‐dependent and therefore only allow for the deuteration of aldehydes, ketones or esters, using a large excess of D_2_O.[^16^, ^17^, ^18^, ^19^, ^20^, ^21^, ^22^, ^23^] α‐Deuterated amides have occasionally been prepared during the course of mechanistic studies,[^24^, ^25^, ^26^, ^27^, ^28^, ^29^] with only few reports of these valuable compounds as the actual targets. The α‐deuteration of amides can be accomplished employing a MeO^−^/MeOD system at elevated temperatures. However, this requires several cycles to achieve high levels of labelling.[^30^, ^31^] In addition to thwarting any possibility of chemoselective labelling in compounds carrying multiple carbonyl functionalities, any base‐sensitive functional groups, or those prone to solvolysis, can be potentially negatively impacted (Scheme 1 A). A further straightforward approach to α‐deuterated amides involves deprotonation with *sec*‐BuLi and subsequent quenching of the resulting anion with D_2_O (Scheme 1 B),^[32]^ although the use of a very strong base once more limits potential functional‐group tolerance. Recently, Atzrodt and Derdau described an iridium‐catalyzed deuteration of aliphatic amides using gaseous D_2_ that was even applicable to small peptides (Scheme 1 C).^[33]^

**Scheme 1:**
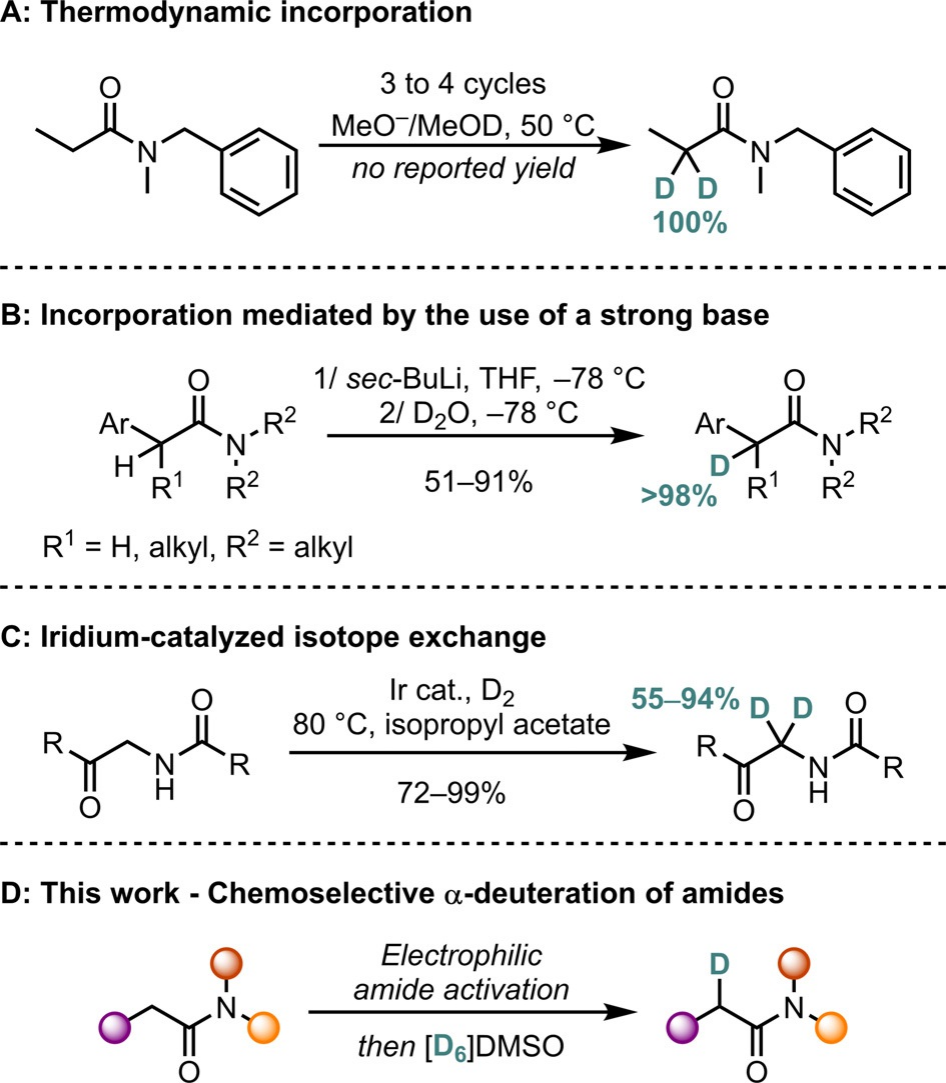
Different approaches to incorporate a deuterium atom in the α‐position of an amide.

Our group has developed research programs centered on the chemoselective, electrophilic activation of amides,[^34^, ^35^, ^36^, ^37^] as well as the sigmatropic rearrangement chemistry of aryl and vinyl sulfoxides.[^38^, ^39^, ^40^, ^41^, ^42^, ^43^] During ongoing studies into the reactivity of keteniminium ions with various classes of sulfoxides, we noticed that the reaction with the simplest sulfoxide, DMSO (dimethyl sulfoxide), led to recovery of seemingly unreacted starting material. Our interest having been sparked by the unexpected result, we performed the reaction with deuterated dimethyl sulfoxide ([D_6_]DMSO) and, surprisingly, we observed the α‐incorporation of a deuterium atom (Scheme 1 D). Eyeing the aforementioned potential benefits of a mild and chemoselective α‐deuteration, we set out to find optimal reaction conditions using the amide **1 a** as a model substrate (Table 1).

**Table 1:** Optimization table.

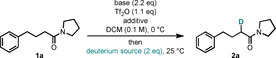				
Entry	Base	D source	Additive	Incorporation [%]^[a]^
1	pyridine	[D_6_]DMSO	–	43
2	2‐F‐pyr	[D_6_]DMSO	–	40
3	2‐Cl‐pyr	[D_6_]DMSO	–	70
4	2‐Br‐pyr	[D_6_]DMSO	–	72
5	2‐I‐pyr	[D_6_]DMSO	–	73
6	2‐I‐pyr	D_2_O^[b]^	–	55
7	2‐I‐pyr	[D_3_]AcOD	–	40
8	2‐I‐pyr	[D_6_]DMSO	4 Å MS	89
9	2‐I‐pyr (1.1 equiv)	[D_6_]DMSO	4 Å MS	26
10	2‐I‐pyr (3.3 equiv)	[D_6_]DMSO	4 Å MS	90

[a] Reactions were performed on a 0.2 mmol scale and deuterium incorporation was measured by ^1^H NMR on the crude mixture after work‐up. [b] 5 equiv.

Our investigations commenced with a broad screening of reaction conditions (Table 1, see the SI for a complete optimization table). The influence of a variety of different bases was evaluated (Table 1, entries 1 to 5), with 2‐iodopyridine (2‐I‐pyr) ultimately proving most suited. [D_6_]DMSO was found to be the most effective source of deuterium, with D_2_O and [D_4_]acetic acid ([D_3_]AcOD) affording considerably lower degrees of incorporation (Table 1, entries 6 and 7). Introduction of molecular sieves (4 Å MS) led to an increase in deuterium incorporation, while variation of the stoichiometry of the base did not prove beneficial (Table 1, entries 8 to 10).

Apart from the potential synthetic practicability of this method, the mechanistic aspects are highly intriguing. Our initial hypothesis started out with the textbook electrophilic activation of amides with trifluoromethanesulfonic anhydride (Tf_2_O) in the presence of 2‐halopyridines to form a keteniminium ion **4** and its stabilized adduct **3** (Scheme 2 A). In analogy to the previously described methodologies, we then assumed that addition of [D_6_]DMSO to the keteniminium intermediate **4** generates intermediate **5**. At this point, we proposed the latter to undergo a retro‐ene fragmentation to yield the deuterated product **2** through simultaneous cleavage of the C−D and the S−O bonds.[^44^, ^45^, ^46^, ^47^, ^48^, ^49^, ^50^] In studies reported during the preparation of this manuscript, Movassaghi and co‐workers proposed a similar mechanistic pathway.^[51]^

**Scheme 2:**
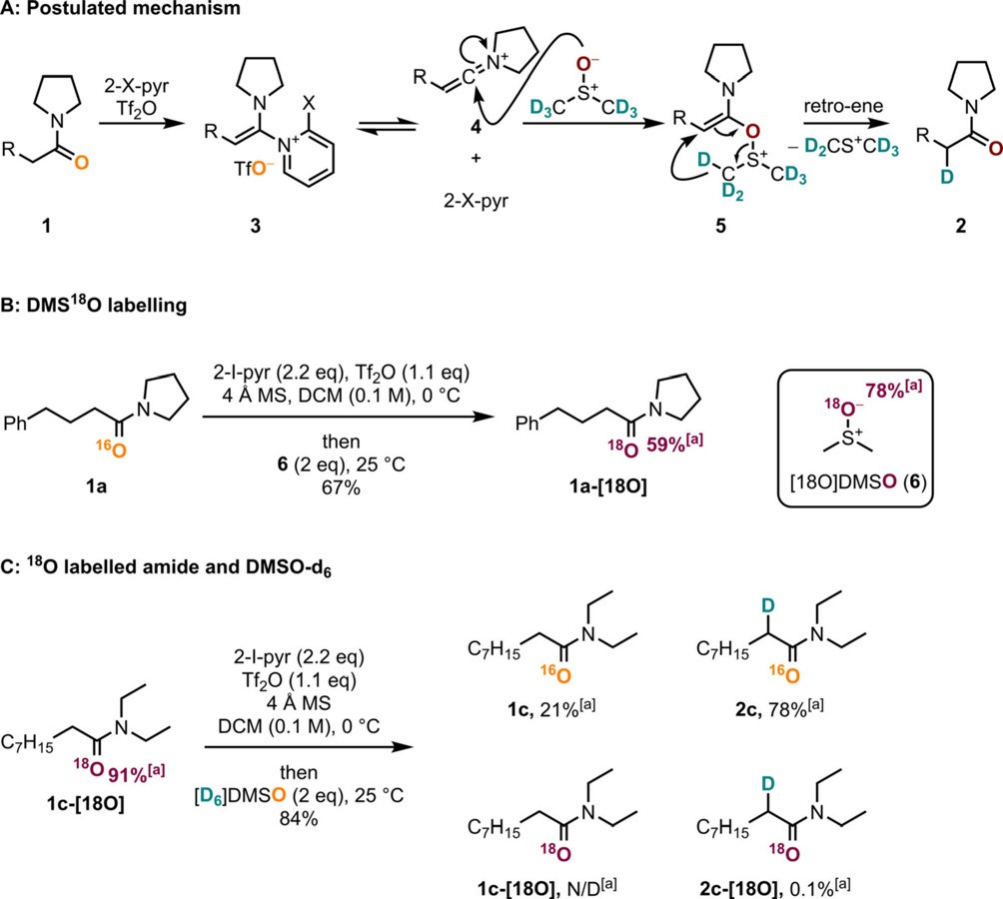
Mechanistic studies. [a] Percentages are relative intensities of the [*M*+Na^+^] ion measured by HRMS.

In this mechanistic proposal, the oxygen atom is ultimately transferred from DMSO to the amide. In support of this assumption, when **1 a** was treated with isotopically labelled [18O]DMSO (**6**) after amide activation, ^18^O incorporation into the product **1 a‐[18O]** was observed (Scheme 2 B). In a further experiment, ^18^O‐labelled amide **1 c‐[18O]** was treated with [D_6_]DMSO following activation, and afforded the ^16^O/D combination as the main product **2 c** (Scheme 2 C). These results confirm unambiguously that the carbonyl oxygen of the deuterated products stems from DMSO, lending strong support to our mechanistic hypothesis.

To further corroborate the reaction mechanism, we undertook DFT analysis of the reaction system, which showed the proposed pathway to be thermodynamically favorable by 51.0 kcal mol^−1^ (Scheme 3). Starting from **1 a**, the amide follows a classical amide activation pathway^[52]^ to yield ion pairs **A** (**A_E_** at 0.7 kcal mol^−1^ and **A_Z_** at −1.4 kcal mol^−1^) after addition of dimethyl sulfoxide to the keteniminium ion **4** (see Scheme 2 A). Deuterium transfer takes place in a concerted fashion through **TS_E_** or **TS_Z_** (8.4 and 12.0 kcal mol^−1^, respectively) in a retro‐ene‐type reaction, generating product **2 a** and methylene sulfonium triflate.

**Scheme 3:**
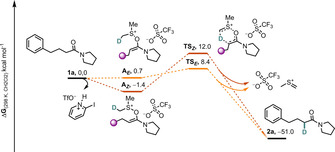
Computed reaction profile for the amide deuteration through a retro‐ene‐type reaction at the PBE0/def‐2SVP level including solvation factor SMD(CH_2_Cl_2_) at 298.15 K. Free energies are reported using **1 a** as the reference point.

Having established optimized reaction conditions and with a better understanding of the mechanistic intricacies of this transformation, the applicability of this reaction to different amides was explored (Scheme 4). Initial focus was placed on the substitution patterns tolerated at the amide nitrogen with different carbon chains. A broad range of tertiary amides derived from dimethyl‐ (**2 b**), diethyl‐ (**2 c**), diallyl‐ (**2 d**) and dibenzylamine (**2 e**), as well as pyrrolidine (**2 a** and **2 i**), piperidine (**2 f**) and azepane (**2 g**) were successfully deuterated. Notably, deuterated lactam **2 h** was also formed in excellent yield and with a high level of deuterium incorporation. We then turned our attention to the carbon chain and the tolerance of reactive functional groups. We were pleased to find that the reaction displayed good functional‐group tolerance, leaving alkyne (**2 j**), alkene (**2 k**), ester (**2 l**), methyl ketone (**2 m**), nitrile (**2 n**), halide (**2 o** and **2 p**) and trifluoromethyl (**2 q**) moieties untouched. Satisfyingly, the amide obtained from the natural product dehydrocholic acid (**2 r**) also afforded the desired product with high levels of deuterium incorporation and no noticeable labelling around the ketone functionalities. In terms of limitations, we found that amides bearing bulkier substituents on either side of the central sp^2^ carbon, such as **2 s** and **2 t**, were less amenable to this protocol.

**Scheme 4:**
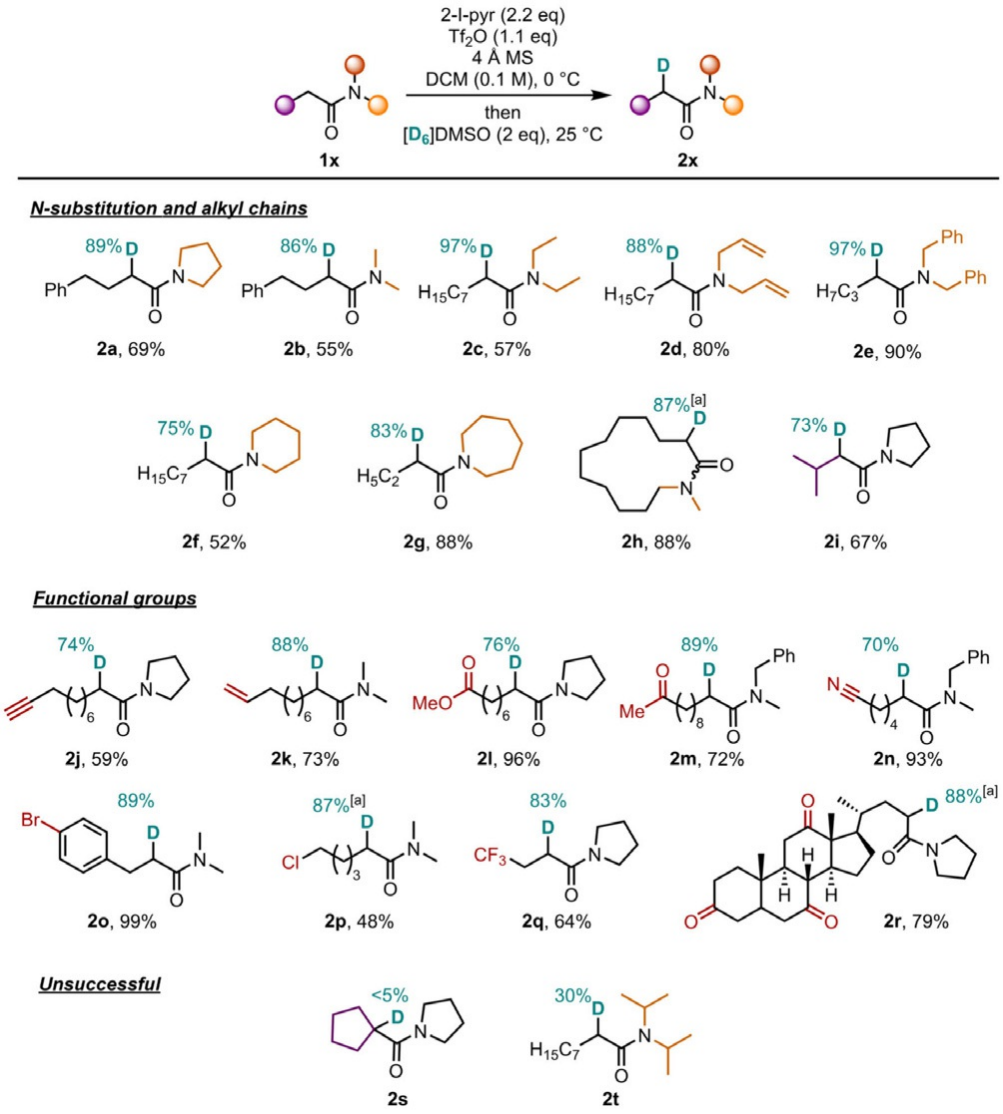
Scope of deuterated amides. Reactions were performed on a 0.2 mmol scale. Deuterium incorporation was determined by ^1^H NMR. All yields refer to isolated materials (see the Supporting Information for details). [a] Deuterium incorporation was determined by HRMS.

Lastly, we were eager to address the question, whether this approach is capable to similarly deliver α‐bisdeuterated amides, as such compounds promise more wide‐spread applicability in the study of biological and medicinal systems.[^10^, ^11^] To this end, monodeuterated amide **2 e** (97 % D) was subjected to the reaction conditions, providing the desired product **7** in 61 % yield with 78 % of bisdeuteration (Eq. 1).^[53]^ While a pronounced kinetic isotope effect is not uncommon for enolization,^[54]^ this result must still be highlighted for its high selectivity.
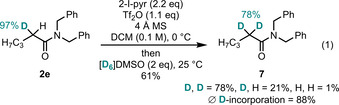



In conclusion, we have presented the highly chemoselective α‐deuteration of amides via a retro‐ene reaction triggered by the addition of [D_6_]DMSO to activated amides. Experimental mechanistic probing and DFT analysis shed light on this intriguing process that is able to tolerate a broad range of functional groups and tertiary amides. Good yields and high levels of deuterium incorporation were obtained throughout. The possibility to perform chemoselective bisdeuteration was established, adding to the potential applicability of this method in the study of reaction mechanisms and in life sciences.

## Conflict of interest

The authors declare no conflict of interest.

## Supporting information

As a service to our authors and readers, this journal provides supporting information supplied by the authors. Such materials are peer reviewed and may be re‐organized for online delivery, but are not copy‐edited or typeset. Technical support issues arising from supporting information (other than missing files) should be addressed to the authors.

Supplementary
