# Supplementary material for: Chemoselective Alpha‐Deuteration of Amides via Retro‐ene Reaction
Source: Chemistry. 2020 Nov 17;26(67):15509–12. doi: 10.1002/chem.202004103 (PMC7756638; doi:10.1002/chem.202004103)
Supplement: Supplementary file 1 — Supplementary [file CHEM-26-15509-s001.pdf]

Chemistry–A European Journal

Supporting Information

Chemoselective Alpha-Deuteration of Amides via Retro-ene Reaction

Vincent Porte,[a] Giovanni Di Mauro+,[a] Manuel Schupp+,[a, b] Daniel Kaiser+,[a] and Nuno Maulide*[a, b]

Table of Contents

1. General Information 2

2. Optimization 3

3. Experimental Procedures 4

   3.1. Synthesis of Starting Materials 4

   3.2. Synthesis of α-Deuterated Amides 11

   3.3. Specific Reactions and Mechanistic Studies 26

4. NMR Spectra 30

4.1. NMR of Starting Materials 30

4.2. NMR of Products 34

4.3. NMR of Specific Reactions and Mechanistic Studies 54

5. Computational Details 57

6. References 63

1. General Information

Unless otherwise stated, all glassware was flame-dried before use and all reactions were performed under an atmosphere of argon. All solvents were distilled from appropriate drying agents prior to use or used as received, if anhydrous. All reagents were used as received from commercial suppliers unless otherwise stated. Trifluoromethanesulfonic anhydride (triflic anhydride, Tf_2O) was distilled over P_4O_{10} prior to use and stored under inert atmosphere in the fridge for a maximum of roughly three weeks.[1] DMSO-d_6 was purchased from Eurisotop (10 mL septum vial). Reaction progress was monitored by thin layer chromatography (TLC) performed on aluminum plates coated with silica gel F254 with 0.2 mm thickness. Chromatograms were visualized by fluorescence quenching with UV light at 254 nm or by staining using potassium permanganate. Flash column chromatography was performed using silica gel 60 (230-400 mesh, Merck and co.) or prepacked columns (Chromabond silica) using a Biotage Selekt Flash Purification System. Neat infrared spectra were recorded using a Perkin-Elmer Spectrum 100 FT-IR spectrometer. Wavenumbers (ν_{max}) are reported in cm^{-1}. Mass spectra were obtained using a Finnigan MAT 8200 or (70 eV) or an Agilent 5973 (70 eV) spectrometer, using electrospray ionization (ESI). All ^{1}H NMR and ^{13}C NMR spectra were recorded using a Bruker AV-400, AV-600 and AV-700 spectrometer at 300K. Chemical shifts are given in parts per million (ppm, δ), referenced to the solvent peak of $CDCl_3$, defined at δ = 7.26 ppm (^{1}H-NMR) and δ = 77.16 ppm (^{13}C-NMR). Coupling constants are quoted in Hz (J). ^{1}H NMR splitting patterns are designated as singlet (s), doublet (d), triplet (t), quartet (q) as they appeared in the spectrum. If the appearance of a signal differs from the expected splitting pattern, the observed pattern is designated as apparent (app). Splitting patterns that could not be interpreted or easily visualized are designated as multiplet (m) or broad (br).

2. Optimization

Table 1: Optimization table - deuterium incorporation was measured by ^{1}H NMR on the crude mixture after work-up. Reactions were performed on a 0.2 mmol scale.

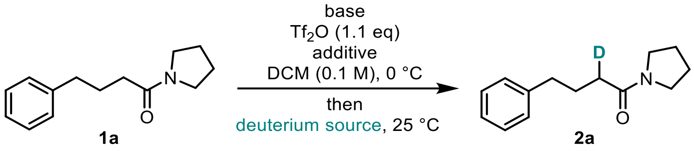

Entry	Base	D source	Additive	D incorporation (%)
1	Pyridine (2.2 eq)	DMSO-d_6 (2 eq)	/	43
2	2-F-pyr (2.2 eq)	DMSO-d_6 (2 eq)	/	40
3	2-Cl-pyr (2.2 eq)	DMSO-d_6 (2 eq)	/	70
4	2-Br-pyr (2.2 eq)	DMSO-d_6 (2 eq)	/	72
5	2-I-pyr (2.2 eq)	DMSO-d_6 (2 eq)	/	73
6	2-Nitro-pyr (2.2 eq)	DMSO-d_6 (2 eq)	/	0
7	2-OMe-pyr (2.2 eq)	DMSO-d_6 (2 eq)	/	59
8	Collidine (2.2 eq)	DMSO-d_6 (2 eq)	/	25
9	2-I-pyr (2.2 eq)	D_2O (5 eq)	/	55
10	2-I-pyr (2.2 eq)	D_2O (10 eq)	/	50
11	2-I-pyr (2.2 eq)	AcOD (2 eq)	/	40
12	**2-I-pyr (2.2 eq)**	**DMSO-d_6 (2 eq)**	**4 Å MS**	**89**
13	2-I-pyr (1.1 eq)	DMSO-d_6 (2 eq)	4 Å MS	26
14	2-I-pyr (3.3 eq)	DMSO-d_6 (2 eq)	4 Å MS	90

3. Experimental Procedures

3.1. Synthesis of Starting Materials

a) 4-Phenyl-1-(pyrrolidin-1-yl)butan-1-one (1a)

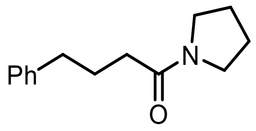

The amide was synthesized following a previously described procedure.[2]

b) *N,N*-Dimethyl-4-phenylbutanamide (1b)

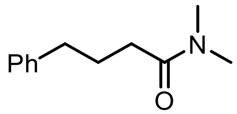

The amide was synthesized following a previously described procedure.[3]

c) *N,N*-Diethylnonanamide (1c)

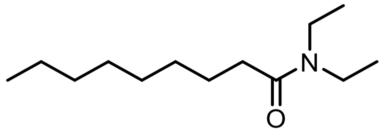

The amide was synthesized following a previously described procedure.[4]

d) *N,N*-Diallylnonanamide (1d)

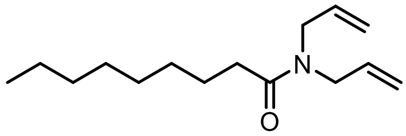

The amide was synthesized following a previously described procedure.[4]

e) *N,N*-Dibenzylpentanamide (1e)

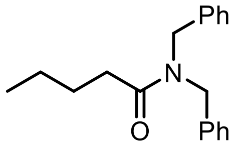

The amide was synthesized following a previously described procedure.[5]

f) 1-(Piperidin-1-yl)nonan-1-one (1f)

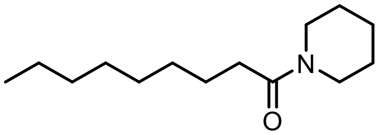

The amide was synthesized following a previously described procedure.[2]

g) 1-(Azepan-1-yl)butan-1-one (1g)

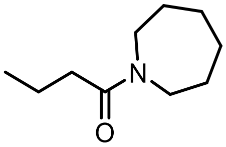

The amide was synthesized following a previously described procedure.[5]

h) 1-Methylazacyclotridecan-2-one (1h)

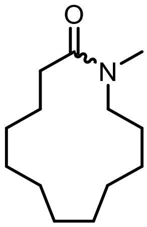

The amide was synthesized following a previously described procedure.[5]

i) 3-Methyl-1-(pyrrolidin-1-yl)butan-1-one (1i)

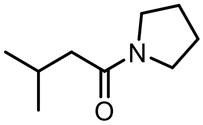

The amide was synthesized following a previously described procedure.[6]

j) 1-(Pyrrolidin-1-yl)undec-10-yn-1-one (1j)

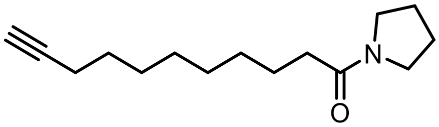

The amide was synthesized following a previously described procedure.[5]

k) *N,N*-Dimethylundec-10-enamide (1k)

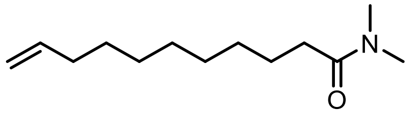

To a solution of dimethylamine (20.7 mL, 31.3 mmol, 2.1 equiv.) in dichloromethane (310 mL, 0.1 M) cooled with an ice bath (0 °C), 10-undecenoyl chloride (3.23 mL, 15 mmol, 1.0 equiv.) was added dropwise. After stirring for 12 h at room temperature, NH_4Cl (sat. aq.) was added and the resulting biphasic mixture was extracted with EtOAc. The organic phase was dried (Na_2SO_4), filtered and concentrated under reduced pressure. The resulting crude mixture was purified by flash chromatography (heptane/EtOAc) to yield *N,N*-dimethylundec-10-enamide as a colorless oil (2.78 g, 13.1 mmol, 88%). (see NMR)

^{1}H NMR (400 MHz, $CDCl_3$): δ 5.94 – 5.67 (m, 1H), 5.07 – 4.83 (m, 2H), 2.99 (s, 3H), 2.93 (s, 3H), 2.29 (t, *J* = 7.7 Hz, 2H), 2.03 (q, *J* = 6.9 Hz, 2H), 1.67 – 1.56 (m, 2H), 1.42 – 1.18 (m, 10H) ppm.
^{13}C NMR (101 MHz, $CDCl_3$): δ 173.4, 139.4, 114.2, 37.4, 35.5, 33.9, 33.6, 29.6, 29.5, 29.5, 29.2, 29.0, 25.3 ppm.
IR (neat) ν_{max}: 2924, 2853, 1643, 1395, 907.
HRMS (ESI$^+$): exact mass calculated for $[M+Na]^+$ ($C_{13}H_{25}NNaO$) requires *m/z* 234.1828, found *m/z* 234.1833.

l) Methyl 9-oxo-9-(pyrrolidin-1-yl)nonanoate (1l)

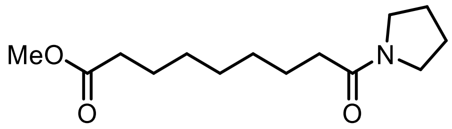

The amide was synthesized following a previously described procedure.[3]

m) *N*-Benzyl-*N*-methyl-10-oxoundecanamide (1m)

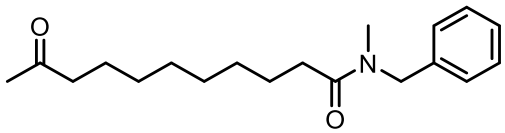

The amide was synthesized following a previously described procedure.[5]

n) *N*-Benzyl-6-cyano-*N*-methylhexanamide (1n)

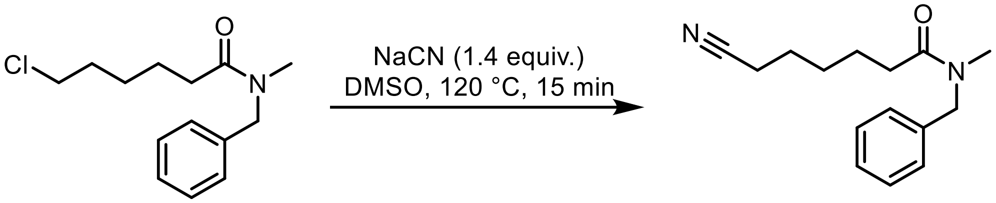

To a solution of *N*-benzyl-6-chloro-*N*-methylhexanamide[4] (300 mg, 1.18 mmol, 1.00 equiv.) in DMSO (800 µL, 1.5 M), sodium cyanide (81.1 mg, 1.66 mmol, 1.40 equiv.) was added and the resulting suspension was heated at 120 °C. After 15 min, the reaction mixture was allowed to cool to ambient temperature and the DMSO was removed under high vacuum. The crude residue was purified by flash column chromatography (heptane/ethyl acetate 1/1) to afford 215 mg (74%) of the title compound as a light-yellow oil. (see NMR)

As a mixture of conformers.

^{1}H NMR (400 MHz, $CDCl_3$): δ 7.40 – 7.20 (m, 4H), 7.17 – 7.13 (m, 1H), 4.56 (app d, J = 23.6 Hz, 2H), 2.94 (app d, J = 19.0 Hz, 3H), 2.43 – 2.30 (m, 4H), 1.78 – 1.61 (m, 4H), 1.58 – 1.42 (m, 2H).

^{13}C NMR (101 MHz, $CDCl_3$): δ 173.0, 172.6, 137.6, 136.8, 129.1, 128.7, 128.2, 127.8, 127.5, 126.3, 119.8, 53.5, 51.0, 34.9, 34.2, 33.2, 32.7, 28.6, 28.5, 25.5, 25.4, 24.5, 24.3, 17.2, 17.1.

IR (neat) ν_{max}: 2939, 1640, 1493, 1453, 1406, 1120, 735.

HRMS (ESI$^+$): exact mass calculated for $[M+Na]^+$ ($C_{15}H_{20}N_2NaO$) requires *m/z* 267.1468, found *m/z* 267.1466.

o) 3-(4-Bromophenyl)-*N,N*-dimethylpropanamide (1o)

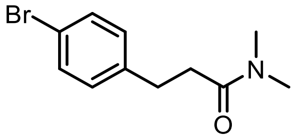

The amide was synthesized following a previously described procedure.[2]

p) 6-Chloro-*N,N*-dimethylhexanamide (1p)

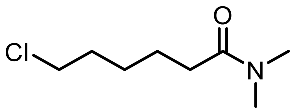

To a solution of 6-chlorohexanoic acid (3.0 g, 19.9 mmol, 1 equiv.) in dichloromethane (30 mL, 0.67 M) cooled in an ice-bath, was added thionyl chloride (1.76 mL, 23.9 mmol, 1.2 equiv.) followed by *N,N*-dimethylformamide (16 µL, 0.20 mmol, 0.01 equiv.). The resulting solution was allowed to return to ambient temperature and was stirred for 2 h. After this time, the volatiles were removed, the crude mixture was dissolved once again in dichloromethane (16.5 mL, 1.2 M) and was subsequently cooled with an ice-bath followed by addition of dimethylamine (33 mL, 49.8 mmol, 2.5 equiv.). After stirring at ambient temperature for 16 h, NH_4Cl (sat. aq.) was added and the resulting biphasic mixture was extracted with EtOAc. The organic phase was dried (Na_2SO_4), filtered and concentrated under reduced pressure. The crude mixture was purified by flash chromatography (heptane/EtOAc) to yield 6-chloro-*N,N*-dimethylhexanamide as a colorless oil (2.07 g, 11.7 mmol, 59%). (see NMR)

^{1}H NMR (400 MHz, $CDCl_3$): δ 3.54 (t, *J* = 6.7 Hz, 2H), 3.00 (s, 3H), 2.94 (s, 3H), 2.32 (t, *J* = 7.5 Hz, 2H), 1.86 – 1.74 (m, 2H), 1.70 – 1.63 (m, 2H), 1.55 – 1.42 (m, 2H) ppm.
^{13}C NMR (101 MHz, $CDCl_3$): δ 172.9, 45.1, 37.4, 35.5, 33.2, 32.6, 26.8, 24.5 ppm.
IR (neat) ν_{max}: 2937, 2866, 1639, 1396.
HRMS (ESI$^+$): exact mass calculated for $[M+Na]^+$ ($C_8H_{16}ClNNaO$) requires *m/z* 200.0813, found *m/z* 200.0807.

q) 4,4,4-Trifluoro-1-(pyrrolidin-1-yl)butan-1-one (1q)

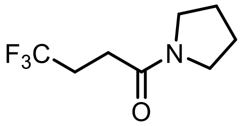

The amide was synthesized following a previously described procedure.[5]

r) (8R,9S,10S,13R,14S,17R)-10,13-Dimethyl-17-((R)-5-oxo-5-(pyrrolidin-1-yl)pentan-2-yl)dodecahydro-3H-cyclopenta[a]phenanthrene-3,7,12(2H,4H)-trione (1r)

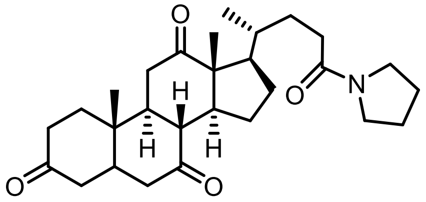

The amide was synthesized following a previously described procedure.[5]

s) Cyclopentyl(pyrrolidin-1-yl)methanone (2s)

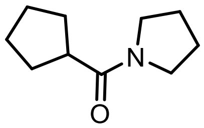

To a solution of pyrrolidine (164 µL, 2.0 mmol, 1.0 equiv.), triethylamine (279 µL, 2.0 mmol, 1.0 equiv.), 1-hydroxybenzotriazole (270 mg, 2.0 mmol, 1.0 equiv.) and 1-ethyl-3-(3-dimethylaminopropyl)carbodiimide hydrochloride (311 mg, 2.0 mmol, 1.0 equiv.) in dichloromethane (20 mL, 0.1 M) was added cyclopentanecarboxylic acid (228 mg, 2.0 mmol, 1.0 equiv.) and the resulting solution was stirred at ambient temperature for 14 h. After this time, the organic solution was extracted sequentially with 0.5 M HCl, $NaHCO_3$ (sat. aq.) and saturated NaCl (sat. aq.). The washed solution was dried over (Na_2SO_4), filtered and concentrated under reduced pressure. The resulting crude material was purified by flash column chromatography (heptane/EtOAc) to yield cyclopentyl(pyrrolidin-1-yl)methanone as a yellow oil (209 mg, 1.25 mmol, 63%). All analytical data were in good accordance with reported data.[7]

t) *N,N*-Diisopropylnonanamide (1t)

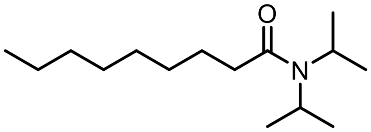

To a solution of diisopropylamine (1.05 mL, 7.5 mmol, 1.5 equiv.) and triethylamine (2.09 mL, 15 mmol, 3 equiv.) in dichloromethane (25 mL) cooled with an ice bath (0 °C) was added dropwise nonanoyl chloride (0.90 mL, 5 mmol, 1.0 equiv.). After stirring for 12 h at ambient temperature, NH_4Cl (sat. aq.) and aqueous layer was extracted with dichloromethane, dried (Na_2SO_4) and concentrated under reduced pressure. The crude mixture was purified by flash chromatography (hexane/EtOAc) to yield *N,N*-dimethylundec-10-enamide as a yellow oil (0.93 g, 3.85 mmol, 77%). (see NMR)

^{1}H NMR (600 MHz, CDCl$_3$): δ 4.05 – 3.94 (m, 1H), 3.47 (b, 1H), 2.29 – 2.23 (t, *J* = 8 Hz, 2H), 1.71 – 1.47 (m, 2H), 1.37 (d, *J* = 6.5 Hz, 6H), 1.30 – 1.26 (m, 10H), 1.19 (d, *J* = 6.5 Hz, 6H), 0.87 (t, *J* = 6.3 Hz, 3H) ppm.
^{13}C NMR (151 MHz, CDCl$_3$): δ 172.3, 48.4, 45.6, 35.6, 32.0, 29.7, 29.6, 29.3, 25.6, 22.8, 21.2, 20.9, 14.2 ppm.
IR (neat) ν_{max}: 2961, 2924, 2854, 1637, 1438.
HRMS (ESI$^+$): exact mass calculated for $[M+Na]^+$ ($C_{15}H_{31}NNaO$) requires *m/z* 264.2298, found *m/z* 264.2298.

3.2. Synthesis of α-Deuterated Amides

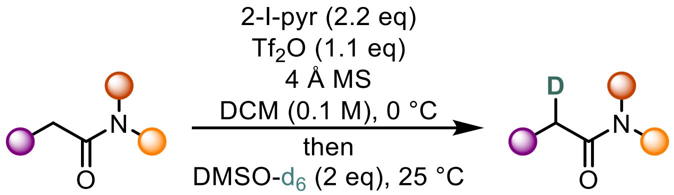

<u>General procedure:</u>

To a 5 mL Schlenk tube containing molecular sieves (4 Å) was added a solution of amide **1** (0.20 mmol, 1.0 equiv.) in dichloromethane (2 mL, 0.1 M), followed by 2-iodopyridine (47 µL, 0.44 mmol, 2.2 equiv.). The mixture was cooled using an ice bath (0 °C) and trifluoromethanesulfonic anhydride (Tf_2O, 37 µL, 0.22 mmol, 1.1 equiv.) was added. The resulting mixture was stirred at 0 °C for 15 min and coloration of the solution was observed. After this time, DMSO-d_6 (28 µL, 0.40 mmol, 2 equiv.) was added and mixture was allowed to warm to ambient temperature (between 23 °C and 25 °C). With time, progressive discoloration was observed. After stirring at ambient temperature for 3 h, water (1.5 mL) was added. The aqueous layer was removed and the organic phase was filtered over $MgSO_4$ with dichloromethane. The filtered solution was concentrated under reduced pressure and the crude mixture was purified by flash column chromatography (SiO_2, heptane/ethyl acetate) to afford the corresponding title compound.

Owing to incomplete deuteration, ^{13}C NMR shows multiple signals for some carbons. Where it is not possible to determine whether a signal stems from the deuterated or the non-deuterated compound, both peaks are reported and denoted with an asterisk (*). A signal that can be unambiguously assigned to the non-deuterated compound is denoted with a pound sign ($^{\#}$).

a) 4-Phenyl-1-(pyrrolidin-1-yl)butan-1-one-2-D (2a)

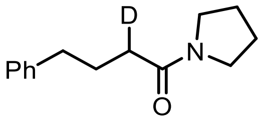

Synthesized following the general procedure: 43.5 mg of amide were used and 30.0 mg of a light yellow oil were obtained (0.14 mmol, 69% yield, 89% incorporation). (see NMR)
^{1}H NMR (400 MHz, CDCl$_3$): δ 7.39 – 7.00 (m, 5H), 3.46 (t, *J* = 6.8 Hz, 2H), 3.32 (t, *J* = 6.7 Hz, 2H), 2.69 (t, *J* = 7.5 Hz, 2H), 2.24 – 2.22 (m, 1.11H + 0.89D), 2.03 – 1.79 (m, 6H) ppm.
^{13}C NMR (101 MHz, CDCl$_3$): δ 171.5, 142.0, 128.7 (2C), 128.5 (2C), 126.0, 46.6, 45.7, 35.5*, 35.5*, 34.0$^{\#}$, 33.5 (t, *J* = 19 Hz), 26.4*, 26.3*, 26.2, 24.5 ppm.
IR (neat) $\nu_{max:}$ 3471, 3025, 2870, 1633, 1428, 749, 701.
HRMS (ESI$^+$): exact mass calculated for $[M+Na]^+$ ($C_{14}H_{18}DNNaO$) requires *m/z* 241.1422, found *m/z* 241.1425.

b) *N,N*-Dimethyl-4-phenylbutanamide-2-D (2b)

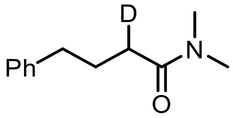

Synthesized following the general procedure: 38.3 mg of amide were used and 21.1 mg of a light yellow oil were obtained (0.11 mmol, 55% yield, 86% incorporation). (see NMR)
^{1}H NMR (600 MHz, CDCl$_3$): δ 7.38 – 7.05 (m, 5H), 2.94 (s, 6H), 2.68 (t, *J* = 7.6 Hz, 2H), 2.39 – 2.22 (m, 1.14H + 0.86D), 1.97 (dd, *J* = 15.1, 7.5 Hz, 2H) ppm.
^{13}C NMR (150 MHz, CDCl$_3$): δ 172.9, 142.0, 128.6 (2C), 128.5 (2C), 126.0, 37.3, 35.5, 35.5, 32.6$^{\#}$, 32.3 (t, *J* = 19 Hz), 26.6 ppm.
IR (neat) $\nu_{max:}$ 3025, 2929, 2860, 1639, 1495, 1395.
HRMS (ESI$^+$): exact mass calculated for $[M+Na]^+$ ($C_{12}H_{16}DNNaO$) requires *m/z* 215.1265, found *m/z* 215.1272.

c) *N,N*-Diethylnonanamide-2-D (2c)

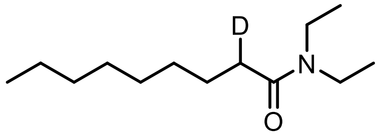

Synthesized following the general procedure: 42.7 mg of amide were used and 24.6 mg of a light yellow oil were obtained after two purifications (0.12 mmol, 57% yield, 97% incorporation). (see NMR)
^{1}H NMR (400 MHz, CDCl$_3$) δ 3.36 (t, *J* = 7.1 Hz, 2H), 3.29 (t, *J* = 7.1 Hz, 2H), 2.29 – 2.23 (m, 1.03H, + 0.97D), 1.69 – 1.60 (m, 2H), 1.35 – 1.21 (m, 10H), 1.16 (t, *J* = 7.1 Hz, 3H), 1.10 (t, *J* = 7.1 Hz, 3H), 0.87 (t, *J* = 6.9 Hz, 3H) ppm.
^{13}C NMR (101 MHz, CDCl$_3$) δ 172.5, 42.1, 40.1, 33.3$^{\#}$, 33.0 (t, *J* = 19 Hz), 32.0, 29.7, 29.6, 29.3, 25.7*, 25.6*, 22.8, 14.6, 14.2, 13.3 ppm.
IR (neat) ν_{max}: 2925, 2854, 1640, 1427.
HRMS (ESI$^+$): exact mass calculated for [M+Na]$^+$ ($C_{13}H_{26}DNNaO$) requires *m/z* 237.2048, found *m/z* 237.2046.

d) *N,N*-Diallylnonanamide-2-D (2d)

Synthesized following the general procedure: 47.5 mg of amide were used and 38.3 mg of a light yellow oil were obtained (0.16 mmol, 80% yield, 88% incorporation). (see NMR)

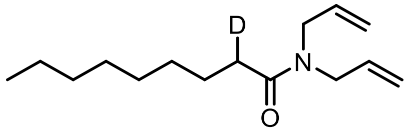

^{1}H NMR (500 MHz, CDCl$_3$) δ 5.81 – 5.67 (m, 2H), 5.21 – 5.07 (m, 4H), 3.97 (d, *J* = 5.9 Hz, 2H), 3.86 (dd, *J* = 2.9, 1.9 Hz, 2H), 2.28 (ddd, *J* = 10.2, 9.1, 5.0 Hz, 1.2H, 0.8D), 1.62 (dd, *J* = 14.3, 7.3 Hz, 2H), 1.35 – 1.22 (m, 10H), 0.86 (t, *J* = 7.0 Hz, 3H) ppm.
^{13}C NMR (126 MHz, CDCl$_3$) δ 173.3, 133.6, 133.2, 117.2, 116.6, 49.3, 47.9, 33.0$^{\#}$, 32.9 (t, *J* = 19 Hz), 32.0, 29.6, 29.5, 29.3, 25.4, 22.8, 14.2 ppm.
IR (neat) ν_{max}: 2923, 2854, 1641, 1434, 1343, 1282, 920.
HRMS (ESI$^+$): exact mass calculated for [M+Na]$^+$ ($C_{15}H_{26}DNNaO$) requires *m/z* 261.2048, found *m/z* 261.2049.

e) *N,N*-Dibenzylpentanamide-2-D (2e)

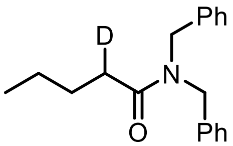

Synthesized following the general procedure: 56.3 mg of amide were used and 51 mg of a light yellow oil were obtained (0.18 mmol, 90% yield, 97% incorporation). (see NMR)

^{1}H NMR (400 MHz, CDCl$_3$) δ 7.39 – 7.15 (m, 10H), 4.61 (s, 2H), 4.45 (s, 2H), 2.41 (t, *J* = 7.6 Hz, 1.03H + 0.97D), 1.73 – 1.67 (m, 2H), 1.40 – 1.33 (m, 2H), 0.91 (t, *J* = 7.3 Hz, 3H) ppm.
^{13}C NMR (101 MHz, CDCl$_3$) δ 173.9, 137.7, 136.8, 129.1 (2C), 128.7 (2C), 128.4 (2C), 127.7, 127.5, 126.5 (2C), 50.0, 48.2, 32.8 (t, *J* = 19 Hz), 27.6, 22.6, 14.0 ppm.
IR (neat) ν_{max}: 2930, 2871, 2360, 1646, 1421, 750, 698.
HRMS (ESI$^+$): exact mass calculated for $[M+Na]^+$ ($C_{19}H_{22}DNNaO$) requires *m/z* 305.1735, found *m/z* 305.1732.

f) 1-(Piperidin-1-yl)nonan-1-one-2-D (2f)

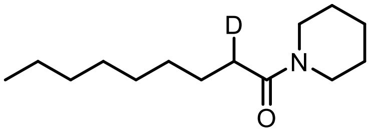

Synthesized following the general procedure: 45.1 mg of amide were used and 23.5 mg of a light yellow oil were obtained (0.10 mmol, 52% yield, 75% incorporation). (see NMR)

^{1}H NMR (400 MHz, CDCl$_3$) δ 3.56 – 3.52 (m, 2H), 3.40 – 3.37 (m, 2H), 2.33 – 2.25 (m, 1.25H + 0.75D), 1.66 – 1.49 (m, 8H), 1.35 – 1.20 (m, 10H), 0.87 (t, *J* = 6.8 Hz, 3H) ppm.
^{13}C NMR (101 MHz, CDCl$_3$) δ 171.72*, 171.70*, 46.9, 42.7, 33.7$^{\#}$, 33.4 (t, *J* = 19 Hz), 32.0, 29.69*, 29.67*, 29.6, 29.3, 26.7, 25.7, 25.65*, 25.59*, 24.8, 22.79, 14.23 ppm.
IR (neat) ν_{max}: 2924, 2856, 1640, 1433.
HRMS (ESI$^+$): exact mass calculated for $[M+Na]^+$ ($C_{14}H_{26}DNONa$) requires *m/z* 249.2048, found *m/z* 249.2043.

g) 1-(Azepan-1-yl)butan-1-one-2-D (2g)

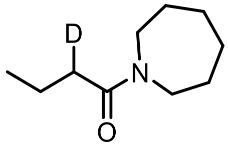

Synthesized following the general procedure: 33.9 mg of amide were used and 29.8 mg of a light yellow oil were obtained (0.18 mmol, 88% yield, 83% incorporation). (see NMR)

^{1}H NMR (400 MHz, $CDCl_3$) δ 3.51 (t, *J* = 6.0 Hz, 2H), 3.42 (t, *J* = 6.0 Hz, 2H), 2.27 (m, 1.17H + 0.83D), 1.78 – 1.62 (m, 6H), 1.55 (d, *J* = 2.5 Hz, 4H), 0.95 (t, *J* = 7.4 Hz, 3H) ppm.
^{13}C NMR (151 MHz, $CDCl_3$) δ 172.8*, 172.8*, 48.0, 46.0, 35.4$^{\#}$, 35.1 (t, *J* = 19 Hz), 29.4, 27.8, 27.2, 27.0, 19.0*, 18.9*, 14.2*, 14.2* ppm.
IR (neat) ν_{max}: 2925, 2855, 1632, 1424.
HRMS (ESI$^+$): exact mass calculated for $[M+Na]^+$ ($C_{10}H_{18}DNONa$) requires *m/z* 193.1422, found *m/z* 193.1423.

h) 1-Methylazacyclotridecan-2-one-2-D (2h)

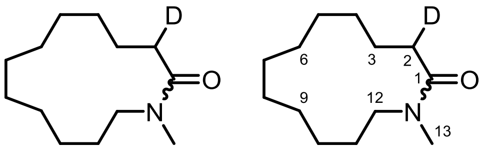

Synthesized following the general procedure: 39.5 mg of amide were used and 34.9 mg of a light yellow oil were obtained (0.16 mmol, 88% yield, 87% incorporation by HRMS, see below) (see NMR, mixture of cis and trans isomers[8])

^{1}H NMR (700 MHz, $CDCl_3$) δ 4.48 – 4.41 (m, H_{12}, minor isomer), 3.29 – 3.26 (td, *J* = 7.2, 1.9 Hz, 2H, H_{12}), 3.01 (s, H_{13}, minor isomer), 2.91 (s, 3H, H_{13}), 2.64 – 2.62 (m, H_{12}, minor isomer), 2.52 – 2.49 (m, H_{12}, minor isomer), 2.34 – 2.30 (m, 1.21H + 0.8D, H_2), 2.11 – 2.09 (m, H_2, minor isomer), 1.77 – 1.19 (m, 18H, H_{3-12}, major and minor) ppm.

^{13}C NMR (101 MHz, $CDCl_3$) δ 173.6, 48.5, 48.2, 47.6, 46.7, 36.0, 33.5, 33.5, 32.1, 32.0, 31.8, 31.6, 27.2, 26.8, 26.4, 26.3, 25.9, 25.9, 25.8, 25.6, 25.5, 25.4, 25.3, 25.2, 25.1, 25.0, 24.9, 24.8, 24.5, 24.4, 24.1, 24.1, 23.7, 23.6, 23.4, 19.0 ppm. *Owing to incomplete deuteration and a mixture of cis and trans isomers, spectra could not be fully attributed and only the main peaks were reported.*

IR (neat) ν_{max}: 2926, 2858, 1631.

HRMS (ESI$^+$): exact mass calculated for $[M+Na]^+$ ($C_{13}H_{24}DNNaO$) requires *m/z* 235.1891, found *m/z* 235.1888.

#	m/z	I
1	210.1850	11966
2	211.1883	1789
3	212.2005	15069
4	213.2071	105365
5	214.2102	13554
6	215.2129	1096
7	228.1953	4438
8	232.1667	4781
9	234.1824	7603
10	235.1888	49704
11	236.1919	6953
12	250.1775	34836
13	251.1804	4294
14	257.1758	810
15	259.1912	1459
16	281.1731	1329
17	297.1680	2225
18	425.4058	1712
19	443.3603	834
20	444.3663	6021
21	445.3712	2325
22	446.3817	9891
23	447.3882	37723
24	448.3913	9534
25	449.3944	1403
26	461.3700	765
27	462.3765	5248
28	463.3797	1490
29	466.3528	1163
30	488.3352	1311

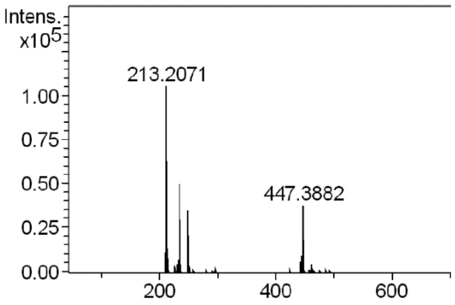

i) 3-Methyl-1-(pyrrolidin-1-yl)butan-1-one-2-D (2i)

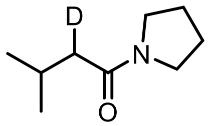

Synthesized following the general procedure: 31 mg of amide were used and 21 mg of a light yellow oil were obtained (0.13 mmol, 67% yield, 73% incorporation) (see NMR)

^{1}H NMR (400 MHz, CDCl$_3$) δ 3.46 (t, *J* = 6.9 Hz, 2H), 3.41 (t, *J* = 6.8 Hz, 2H), 2.21 – 2.11 (m, 2.27H + 0.73D), 1.97 – 1.89 (m, 2H), 1.87 – 1.80 (m, 2H), 0.96 (d, *J* = 6.3 Hz, 6H) ppm.
^{13}C NMR (101 MHz, CDCl$_3$) δ 171.5, 46.9, 45.7, 43.9$^{\#}$, 43.5 (t, *J* = 19 Hz), 26.3, 25.7*, 25.6*, 24.6, 22.87* (2C), 22.82* (2C) ppm.
IR (neat) $\nu_{max:}$ 2955, 2870, 1634, 1367.
HRMS (ESI$^+$): exact mass calculated for $[M+Na]^+$ ($C_9H_{16}DNONa$) requires *m/z* 179.1265, found *m/z* 179.1261.

j) 1-(Pyrrolidin-1-yl)undec-10-yn-1-one-2-D (2j)

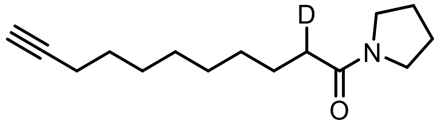

Synthesized following the general procedure: 47.1 mg of amide were used and 28 mg of a light yellow oil were obtained (0.12 mmol, 59% yield, 74% incorporation) (see NMR)

^{1}H NMR (400 MHz, CDCl$_3$) δ 3.46 (t, *J* = 6.8 Hz, 2H), 3.40 (t, *J* = 6.8 Hz, 2H), 2.25 – 2.19 (m, 3.26H + 0.74D), 2.00 – 1.89 (m, 3H), 1.89 – 1.79 (m, 2H), 1.69 – 1.59 (m, 2H), 1.50 – 1.48 (m, 2H), 1.38 – 1.24 (m, 8H) ppm.
^{13}C NMR (151 MHz, CDCl$_3$) δ 172.0, 84.9, 68.2, 46.8, 45.7, 35.0$^{\#}$, 34.7 (t, *J* = 19 Hz), 29.6, 29.5, 29.1, 28.9, 28.6, 26.3, 25.0, 24.6, 18.5 ppm.
IR (neat) $\nu_{max:}$ 3307, 3230, 2928, 2855, 1632, 1427, 627.
HRMS (ESI$^+$): exact mass calculated for $[M+Na]^+$ ($C_{15}H_{24}DNNaO$) requires *m/z* 259.1891, found *m/z* 259.1883.

k) *N,N*-Dimethylundec-10-enamide-2-D (2k)

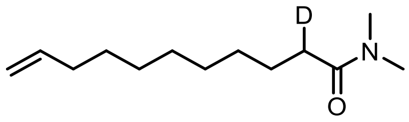

Synthesized following the general procedure: 42.3 mg of amide were used and 31 mg of a light yellow oil were obtained (0.15 mmol, 73% yield, 88% incorporation) (see NMR)

^{1}H NMR (600 MHz, $CDCl_3$) δ 5.87 – 5.72 (m, 1H), 5.05 – 4.83 (m, 2H), 3.00 (s, 3H), 2.94 (s, 3H), 2.37 – 2.21 (m, 1.12H + 0.88D), 2.05 – 2.01 (m, 2H), 1.61 (m, 2H), 1.42 – 1.20 (m, 10H) ppm.
^{13}C NMR (151 MHz, $CDCl_3$) δ 173.4, 139.4, 114.3, 37.4, 35.5, 33.9, 33.6$^{\#}$, 33.4 (t, *J* = 19 Hz), 29.6, 29.6, 29.5, 29.2, 29.1, 25.3 ppm.
IR (neat) ν_{max}: 2924, 2853, 1640, 1393, 907.
HRMS (ESI^{+}): exact mass calculated for $[M+Na]^{+}$ ($C_{13}H_{24}DNNaO$) requires *m/z* 235.1891, found *m/z* 235.1898.

l) Methyl 9-oxo-9-(pyrrolidin-1-yl)nonanoate-2-D (2l)

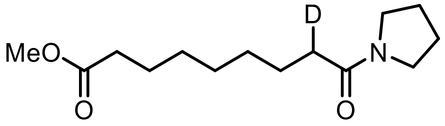

Synthesized following the general procedure: 51.1 mg of amide were used and 49.1 mg of a light yellow oil were obtained (0.19 mmol, 96% yield, 76% incorporation) (see NMR)

^{1}H NMR (400 MHz, $CDCl_3$) δ 3.66 (s, 3H), 3.46 (t, *J* = 6.9, 2H), 3.40 (t, *J* = 6.8 Hz, 2H), 2.31 – 2.22 (m, 3.24H + 0.76D), 1.94 (m, 2H), 1.84 (m, 2H), 1.65 – 1.59 (m, 4H), 1.36 -1.28 (m, 6H) ppm.
^{13}C NMR (101 MHz, $CDCl_3$) δ 174.5, 172.0, 51.6, 46.8, 45.8, 34.9$^{\#}$, 34.6 (t, *J* = 19 Hz), 34.2, 29.4, 29.2, 29.1, 26.3, 25.0, 24.9, 24.6 ppm.
IR (neat) ν_{max}: 2932, 2859, 1736, 1635, 1434, 750.
HRMS (ESI^{+}): exact mass calculated for $[M+Na]^{+}$ ($C_{14}H_{24}DNNaO_3$) requires *m/z* 279.1789, found *m/z* 279.1786.

m) *N*-Benzyl-*N*-methyl-10-oxoundecanamide-2-D (2m)

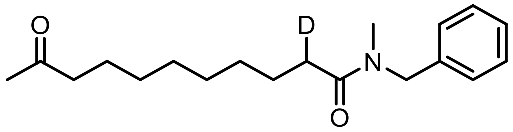

Synthesized following the general procedure: 60.7 mg of amide were used and 43.9 mg of a light yellow oil were obtained (0.14 mmol, 72% yield, 89% incorporation) (mixture of rotamers, see NMR)

^{1}H NMR (400 MHz, $CDCl_3$) δ 7.38 – 7.14 (m, 5H), 4.59/4.53 (12:8, s/s, 2H), 2.93/2.90 (8:12, s/s, 3H), 2.43 – 2.34 (m, 3.11H + 0.89D), 2.13 – 2.12 (m, 3H), 1.71 – 1.61 (m, 2H), 1.60 – 1.52 (m, 2H), 1.39 – 1.21 (m, 8H) ppm.

^{13}C NMR (101 MHz, $CDCl_3$) δ 209.5, 173.8, 173.4, 137.7, 137.0, 129.0, 128.7, 128.2, 127.7, 127.4, 126.4, 53.5, 50.9, 43.9, 34.9, 34.0, 33.5 – 32.7 (m), 30.0, 29.5 – 29.2 (m), 25.4, 25.2, 23.9 ppm.

IR (neat) ν_{max}: 3062, 3029, 2926, 2853, 1712, 1640, 1401.

HRMS (ESI$^+$): exact mass calculated for $[M+Na]^+$ ($C_{19}H_{28}DNNaO_2$) requires *m/z* 327.2153, found *m/z* 327.2150.

n) *N*-Benzyl-6-cyano-*N*-methylhexanamide-2-D (2n)

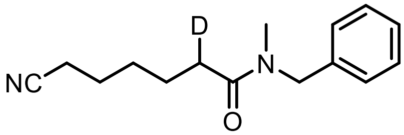

Synthesized following the general procedure: 49 mg of amide were used and 45.7 mg of a light yellow oil were obtained (0.19 mmol, 93% yield, 70% incorporation) (mixture of rotamers in a 1.4:1 ratio, see NMR)

^{1}H NMR (600 MHz, CDCl$_3$) δ 7.37 (t, *J* = 7.5 Hz, 1H), 7.35 – 7.29 (m, 1H), 7.29 – 7.25 (m, 1H), 7.23 (d, *J* = 7.1 Hz, 1H), 7.15 (d, *J* = 7.4 Hz, 1H), 4.59 (s, major rotamer) 4.53 (s, minor rotamer) (2H), 2.96 (s, minor rotamer), 2.92 (s, major rotamer) (3H), 2.41 – 2.32 (m, 3.3H + 0.7D), 1.76 – 1.62 (m, 4H), 1.57 – 1.43 (m, 2H) ppm.
^{13}C NMR (151 MHz, CDCl$_3$) δ 173.0*, 173.0*, 172.6*, 172.6*, 137.6, 136.8, 129.1, 128.7, 128.2, 127.8, 127.5, 126.3, 119.8*, 119.8*, 53.4, 51.0, 50.9, 34.9, 34.2, 33.2$^{\#}$, 32.8 (t, *J* = 19 Hz), 32.7$^{\#}$, 32.4 (t, *J* = 19 Hz), 31.1, 28.6*, 28.6*, 28.5*, 28.5*, 25.5*, 25.4*, 24.5*, 24.4*, 24.3*, 24.2*, 17.2*, 17.2* ppm.
IR (neat) $\nu_{max:}$ 2934, 2867, 1639, 1427, 1403.
HRMS (ESI$^+$): exact mass calculated for $[M+Na]^+$ ($C_{15}H_{19}DN_2ONa$) requires *m/z* 268.1531, found *m/z* 268.1532.

o) 3-(4-Bromophenyl)-*N,N*-dimethylpropanamide-2-D (2o)

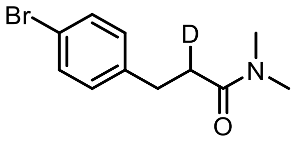

Synthesized following the general procedure: 51.2 mg of amide were used and 50.7 mg of a light yellow oil were obtained (0.19 mmol, 99% yield, 89% incorporation) (see NMR)

^{1}H NMR (600 MHz, CDCl$_3$) δ 7.39 (d, *J* = 8.3 Hz, 2H), 7.10 (d, *J* = 8.2 Hz, 2H), 2.94 (s, 3H), 2.93 (s, 3H), 2.93 – 2.88 (m, 2H), 2.60 – 2.52 (m, 1.11H + 0.89D) ppm.
^{13}C NMR (151 MHz, CDCl$_3$) δ 171.9, 140.6, 131.6 (2C), 130.4 (2C), 119.9, 37.3, 35.1#, 34.7 (t, *J* = 19 Hz), 34.6, 30.7 ppm.
IR (neat) ν_{max}: 3461, 2928, 1640, 1488, 1396, 773.
HRMS (ESI$^+$): exact mass calculated for $[M+Na]^+$ ($C_{11}H_{13}DBrNONa$) requires *m/z* 279.0214, found *m/z* 279.0216.

p) 6-Chloro-*N,N*-dimethylhexanamide-2-D (2p)

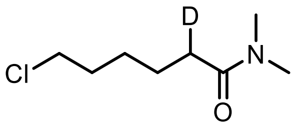

Synthesized following the general procedure: 35.5 mg of amide were used and 17.2 mg of a light yellow oil were obtained (0.09 mmol, 48% yield, 87% incorporation by HRMS, see below) (see NMR)

^{1}H NMR (400 MHz, CDCl$_3$) δ 3.54 (t, *J* = 6.7 Hz, 2H), 3.04 – 2.96 (m, 3H), 2.94 (s, 3H), 2.32 – 2.30 (m, 1H + 1D), 1.88 – 1.74 (m, 2H), 1.66 (m, 2H), 1.50 (m, 2H) ppm.
^{13}C NMR (101 MHz, CDCl$_3$) δ 173.0, 45.1, 37.4, 35.5, 33.2$^{\#}$, 33.07 (t, *J* = 19 Hz), 32.60, 26.9, 24.4 ppm.
IR (neat) ν_{max}: 2935, 2866, 1635, 1394.
HRMS (ESI$^+$): exact mass calculated for $[M+Na]^+$ ($C_8H_{15}DClNNaO$) requires *m/z* 201.0875, found *m/z* 201.0872.

#	m/z	I
1	178.0990	11071
2	178.8985	78
3	179.1054	75560
4	180.0319	106
5	180.1058	6758
6	181.1024	25120
7	182.1055	2174
8	183.1079	141
9	185.1133	81
10	186.0652	201
11	188.0630	76
12	189.1031	106
13	194.0938	1234
14	195.0981	186
15	196.0914	402
16	196.1113	522
17	197.1141	97
18	198.0651	768
19	199.0667	107
20	200.0807	7732
21	201.0872	53834
22	202.0873	4874
23	203.0842	17085
24	204.0873	1450

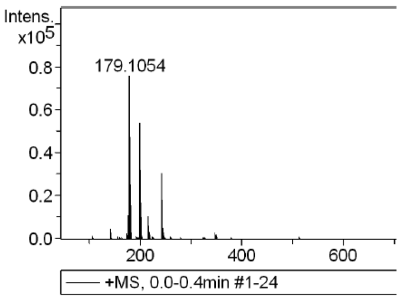

q) 4,4,4-Trifluoro-1-(pyrrolidin-1-yl)butan-1-one-2-D (2q)

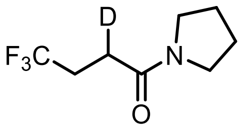

Synthesized following the general procedure: 39 mg of amide were used and 25.1 mg of a light yellow oil were obtained (0.13 mmol, 64% yield, 83% incorporation) (see NMR)

^{1}H NMR (400 MHz, CDCl$_3$) δ 3.47 (t, *J* = 6.9 Hz, 2H), 3.41 (t, *J* = 6.8 Hz, 2H), 2.59 – 2.45 (m, 3.17H + 0.83D), 1.98 (app p, *J* = 6.8 Hz, 2H), 1.87 (app p, *J* = 6.7 Hz, 2H) ppm.
^{13}C NMR (101 MHz, CDCl$_3$) δ 168.3, 127.3 (q, *J* = 275.9 Hz), 46.6, 46.0, 29.4* (app ddd, *J* = 36.8, 28.6, 6.3 Hz), 27.5 –26.9 (m)*, 26.2, 24.5 ppm.
IR (neat) ν_{max}: 2960, 2878, 1639, 1441, 1380, 1131.
^{19}F NMR (376 MHz, CDCl$_3$): δ –66.7 ppm.
HRMS (ESI$^+$): exact mass calculated for $[M+Na]^+$ ($C_8H_{11}DF_3NONa$) requires *m/z* 219.0826, found *m/z* 219.0819.

r) (8R,9S,10S,13R,14S,17R)-10,13-Dimethyl-17-((R)-5-oxo-5-(pyrrolidin-1-yl)pentan-2-yl)dodecahydro-3H-cyclopenta[a]phenanthrene-3,7,12(2H,4H)-trione-2-D (2r)

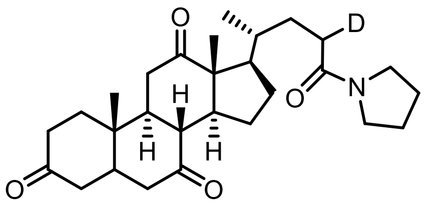

Synthesized following the general procedure but was purified by flash chromatography using a mixture of dichloromethane/MeOH/NH_3: 91.1 mg of amide were used and 72 mg of white solid were obtained (0.16 mmol, 79% yield, 88% incorporation by HRMS, see below) (see NMR)

^{1}H NMR (600 MHz, $CDCl_3$) δ 3.44 (t, *J* = 7 Hz, 2H), 3.40 (t, *J* = 7 Hz, 2H) 2.95–2.80 (m, 3H), 2.33–1.82 (m, 19H + *x*D), 1.66–1.55 (m, 1H), 1.40 - 1.36 (m, 6H), 1.28–1.23 (m, 1H), 1.07 (s, 3H), 0.86 (d, *J* = 6.3 Hz, 3H) ppm.
^{13}C NMR (151 MHz, $CDCl_3$) δ 212.3, 209.2, 208.9, 172.1, 57.1, 51.9, 49.2, 47.0, 46.7, 45.8, 45.7, 45.7, 45.1, 42.9, 38.8, 36.6, 36.1, 35.6*, 35.5*, 35.4, 31.7$^{\#}$, 31.4 (m), 30.4*, 30.4*, 27.6, 26.3, 25.3, 24.5, 22.0, 19.0, 12.0 ppm.
IR (neat) ν_{max}: 2967, 2927, 2867, 1699, 1632, 1423, 736.
HRMS (ESI$^+$): exact mass calculated for $[M+Na]^+$ ($C_{28}H_{40}DNNaO_4$) requires *m/z* 479.2991, found *m/z* 479.2992.

#	m/z	I
1	456.3103	1813
2	457.2055	150
3	457.2514	159
4	457.3169	13187
5	458.3202	3604
6	459.2201	135
7	459.3231	631
8	460.3256	90
9	463.3034	61
10	465.2453	72
11	467.2249	76
12	467.2588	73
13	468.3008	115
14	468.8034	73
15	469.2392	81
16	469.3133	65
17	471.2207	111
18	472.3052	446
19	473.2356	96
20	473.3103	157
21	473.3421	128
22	475.7894	61
23	476.2786	577
24	476.7920	89
25	477.2818	210
26	478.2925	4143
27	479.2992	31331
28	480.3022	8471
29	481.2424	96
30	481.3049	1322

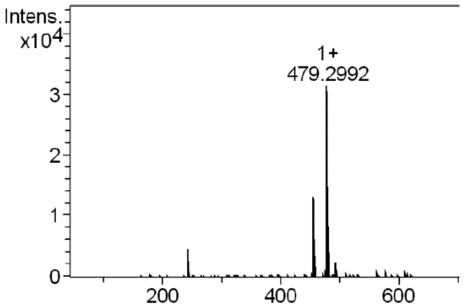

s) *N,N*-Dibenzylpentanamide-2-D_2 (7)

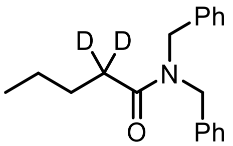

Synthesized following the general procedure: 21 mg of mono-deuterated amide **2e** were used and 12.9 mg of a light yellow oil were obtained (0.045 mmol, 61% yield, 177% incorporation overall, 80% incorporation for second step). (see NMR)

^{1}H NMR (600 MHz, $CDCl_3$) δ 7.39 – 7.15 (m, 10H), 4.60 (s, 2H), 4.45 (s, 2H), 2.42 – 2.39 (m, 0.23H + 1.77D), 1.70 – 1.68 (m, 2H), 1.39 – 1.33 (m, 2H), 0.91 (t, *J* = 7.4 Hz, 3H) ppm.

^{13}C NMR (151 MHz, $CDCl_3$) δ 173.9, 137.7, 136.8, 129.1 (2C), 128.7 (2C), 128.4 (2C), 127.7, 127.5, 126.5 (2C), 50.0, 48.2, 33.0 – 32.7 (m), 27.6, 22.6, 14.0 ppm.

IR (neat) ν_{max}: 2930, 2871, 2360, 1646, 1453, 1418, 732, 699.

HRMS (ESI$^+$): exact mass calculated for $[M+Na]^+$ ($C_{19}H_{21}D_2NNaO$) requires *m/z* 306.1797, found *m/z* 306.1800.

#	m/z	I
1	140.0684	1245
2	181.1013	679
3	257.1474	738
4	280.1697	749
5	281.1756	882
6	282.1845	953
7	283.1918	16580
8	284.1981	64022
9	285.2012	11532
10	286.2044	1248
11	290.2447	1059
12	304.1672	862
13	305.1736	18746
14	306.1800	74794
15	307.1831	13893
16	308.1863	1482
17	312.2265	628
18	480.2508	810
19	587.3571	1473
20	588.3636	8746
21	589.3696	19494
22	590.3730	6950
23	591.3758	1407
24	599.3240	1933
25	600.3294	2572
26	601.3356	3383
27	602.3390	1198
28	1177.6714	667
29	1178.6766	673
30	1179.6832	564

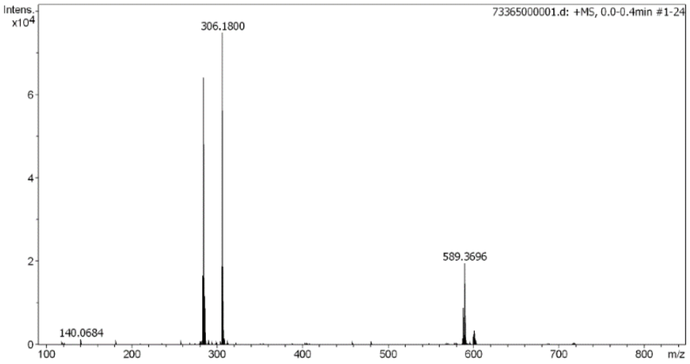

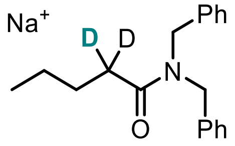

Exact Mass: 306.1797

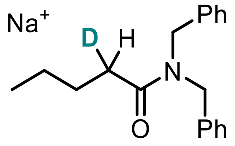

Exact Mass: 305.1735

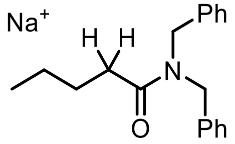

Exact Mass: 304.1672

D, D = **78%**
D, H = **21%**
H, H = **1%**

3.3. Specific Reactions and Mechanistic Studies

a) Dimethylsulfoxide ^{18}O (6)

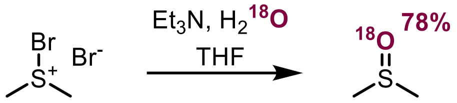

DMS^{18}O (**6**) was synthesized according to a modified literature procedure.[9]

In a three-necked round-bottom flask was added Et_3N (3.1 mL, 4.0 equiv.) freshly distilled over KOH followed by THF (15 mL) and $H_2{}^{18}O$ (99 µL, 1.0 equiv.). Bromodimethylsulfonium bromide (2.49 g, 2.0 equiv.) was added portionwise over 15 min, in order to keep the temperature below 45°C. The resulting precipitate was filtered and washed with diethyl ether. The filtrate was concentrated under reduced pressure and the crude residue was distilled by Kugelrohr (see NMR) – due to a lack of access to the necessary equipment, preparative GC purification, as described in the literature, was not possible.

HRMS (ESI$^+$): exact mass calculated for $[M+Na]^+$ ($C_2H_6Na^{18}OS$) requires *m/z* 103.0074, found *m/z* 103.0071. The ^{18}O content of the labeled DMSO was determined by HRMS at approximately 78%.

#	m/z	I
1	74.0965	1446
2	76.0644	432
3	77.9988	264
4	81.0254	1114
5	86.0963	5765
6	87.0039	271
7	87.0995	375
8	88.4891	282
9	90.5068	309
10	97.4942	281
11	98.5118	343
12	98.9611	499
13	99.5121	452
14	100.0240	430
15	100.0754	1451
16	100.1118	5995
17	101.0028	2176
18	101.1159	510
19	102.0071	316
20	102.0909	5060
21	102.1276	259099
22	103.0071	7856
23	103.0942	839
24	103.1307	19032
25	104.0097	264
26	104.0526	1038
27	104.0954	55188
28	104.1339	535
29	105.0028	415
30	105.0984	3235
31	106.4996	322

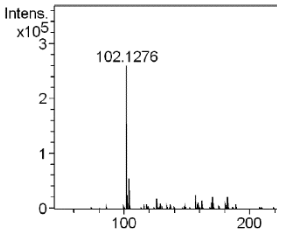

b) 4-Phenyl-1-(pyrrolidin-1-yl)butan-1-one-^{18}O (1a-[18O])

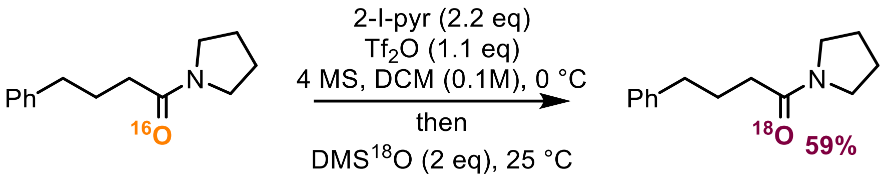

1a **1a-[18O]**

1a-[18O] was synthesized following the general procedure with slight modifications. The reaction mixture was stirred for 18h instead of 3h. 29 mg of a light yellow oil (67%) were obtained (see NMR).

^{1}H NMR (400 MHz, CDCl$_3$): δ 7.39 – 7.00 (m, 5H), 3.46 (t, *J* = 6.8 Hz, 2H), 3.32 (t, *J* = 6.7 Hz, 2H), 2.69 (t, *J* = 7.5 Hz, 2H), 2.24 – 2.22 (m, *J* = 7.5 Hz, 2H), 2.03 – 1.79 (m, 6H) ppm.
HRMS (ESI$^+$): exact mass calculated for [M+Na]$^+$ ($C_{14}H_{18}DNNa^{18}O$) requires *m/z* 242.1401, found *m/z* 242.1402. The ^{18}O content was determined by HRMS at 59% (relative intensity).

#	m/z	I
1	214.2164	86
2	216.1376	102
3	218.1538	11788
4	219.1571	2086
5	220.1582	18539
6	221.1614	2683
7	222.1648	207
8	236.1977	57
9	240.1358	27053
10	241.1391	4500
11	242.1402	39607
12	243.1432	5767
13	244.1464	460
14	252.1354	58
15	254.1402	152
16	256.1095	397
17	257.1122	66
18	258.1136	562
19	259.1170	87
20	286.1297	269
21	288.1341	324
22	289.1376	64
23	300.1010	102
24	302.1060	88
25	457.2809	406
26	458.2844	143
27	459.2858	1175
28	460.2891	392
29	461.2906	884
30	462.2942	240

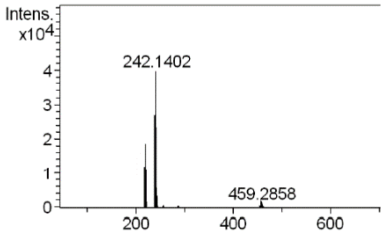

c) *N,N*-Diethylnonanamide-^{18}O (1c-[18O])

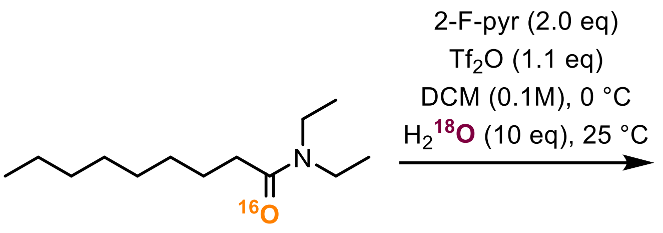

1c **1c-[18O]**

1c-[18O] was prepared from the non-labeled amide **1c** by activation with triflic anhydride (1.1 equiv.) and 2-fluoropyridine (2.0 equiv.) in dichloromethane (0.1 M) at 0 °C for 15 min and subsequent quenching with $H_2{}^{18}O$ (97% ^{18}O, 10 equiv.; vigorous stirring for 1 h) in quantitative yield (91% ^{18}O-incorporation).

HRMS (ESI⁺): exact mass calculated for $[M+Na]^+$ ($C_{13}H_{27}NNa^{18}O$) requires *m/z* 238.2027, found m/z 238.2024.

#	m/z	I
1	214.2162	16148
2	215.2197	3749
3	216.2206	161043
4	217.2237	22386
5	218.2270	1429
6	236.1979	6962
7	237.2015	1587
8	238.2024	69989
9	239.2055	9386
10	240.2087	665
11	244.2054	2492
12	245.2086	480
13	254.1769	1340
14	270.9803	399
15	354.3348	567
16	356.3397	352
17	376.3165	516
18	441.4040	400
19	443.4083	2525
20	444.4112	744
21	449.4045	355
22	451.4108	6366
23	452.4143	2510
24	453.4155	34192
25	454.4184	9555
26	455.4213	1325
27	463.3857	547
28	465.3900	3032
29	466.3930	961
30	907.7906	382

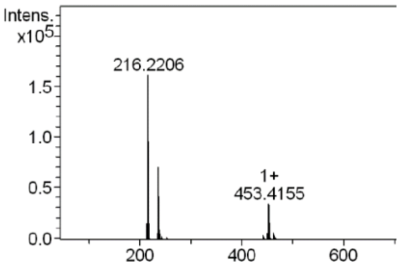

d) *N,N*-Diethylnonanamide-2-D (2c)

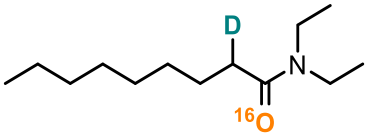

The amide was synthesized following the general procedure with slight modifications. The reaction was performed on a 0.1 mmol scale, 21.5 mg of amide **3c** were used and 18 mg of a light yellow oil were obtained (0.08 mmol, 84% yield, 80% D-incorporation) (see NMR)

^{1}H NMR (700 MHz, $CDCl_3$) δ 3.37 (q, *J* = 7.0 Hz, 2H), 3.30 (q, *J* = 7.2 Hz, 2H), 2.27 (m, 1.20H + 0.80D), 1.63 (m, 2H), 1.29 (m, 10H), 1.17 (t, *J* = 7.1 Hz, 3H), 1.11 (t, *J* = 7.1 Hz, 3H), 0.88 (t, *J* = 6.7 Hz, 3H) ppm.

^{13}C NMR (176 MHz, $CDCl_3$) δ 172.5, 42.1, 40.1, 33.4$^{\#}$, 33.0 (t, *J* = 19 Hz), 32.0, 29.7*, 29.7*, 29.6, 29.4, 25.7*, 25.6*, 22.8, 14.6, 14.3, 13.3 ppm.

HRMS (ESI^{+}): exact mass calculated for $[M+Na]^{+}$ ($C_{13}H_{26}DNNaO$) requires *m/z* 237.2048, found *m/z* 237.2047. This D/^{16}O isotope was found to be the main component between the 4 isotopes presented, expressed percentages (see below) are relative intensities.

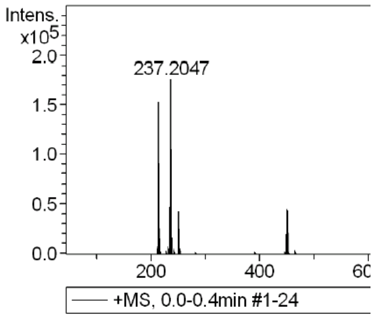

#	m/z	I
1	212.2006	4850
2	213.2044	869
3	214.2164	44168
4	215.2227	152896
5	216.2247	26332
6	217.2268	2328
7	229.2376	290
8	230.2111	2551
9	231.2144	429
10	232.1669	346
11	234.1827	4296
12	235.1866	848
13	236.1983	47159
14	237.1150	512
15	237.2047	175831
16	238.2066	28405
17	239.2088	2714

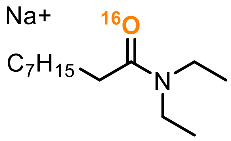

1c, 21%*
Exact Mass: 236.1985

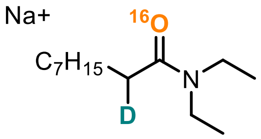

2c, 78%*
Exact Mass: 237.2048

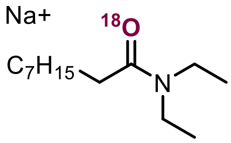

1c-[18O], N/D*
Exact Mass: 238.2027

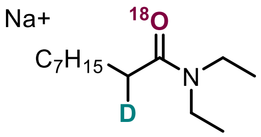

2c-[18O], 0.1%*
Exact Mass: 239.2090

*These numbers refer to relative intensities.

4. NMR Spectra

4.1. NMR of Starting Materials

a) ***N,N*-Dimethylundec-10-enamide (1k)**

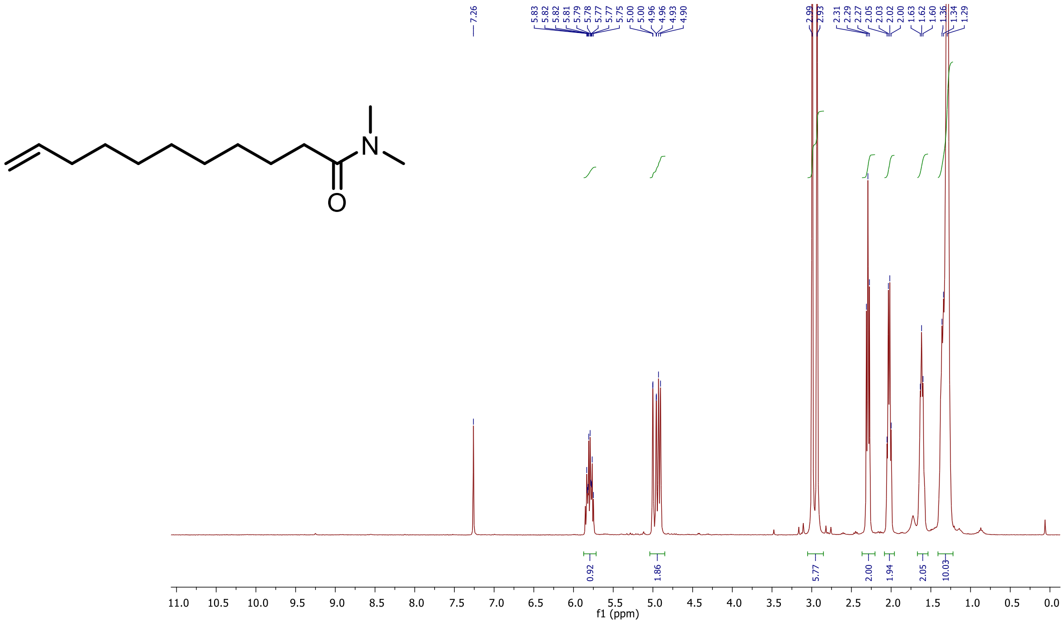

^{1}H
$CDCl_3$
400 MHz

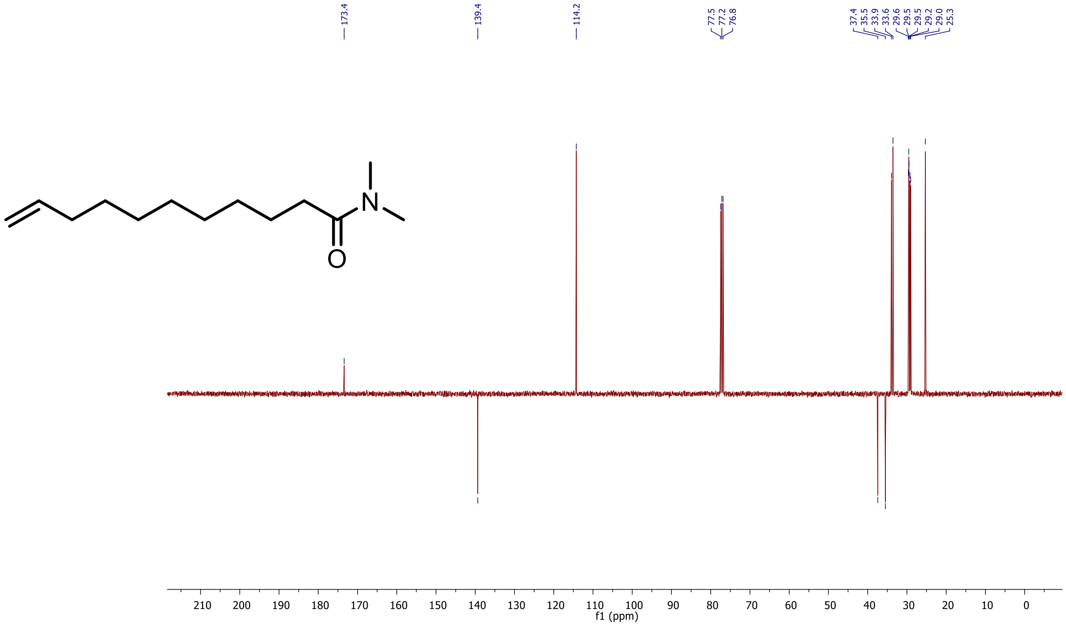

^{13}C
$CDCl_3$
101 MHz

b) 6-Chloro-*N*,*N*-dimethylhexanamide (1p)

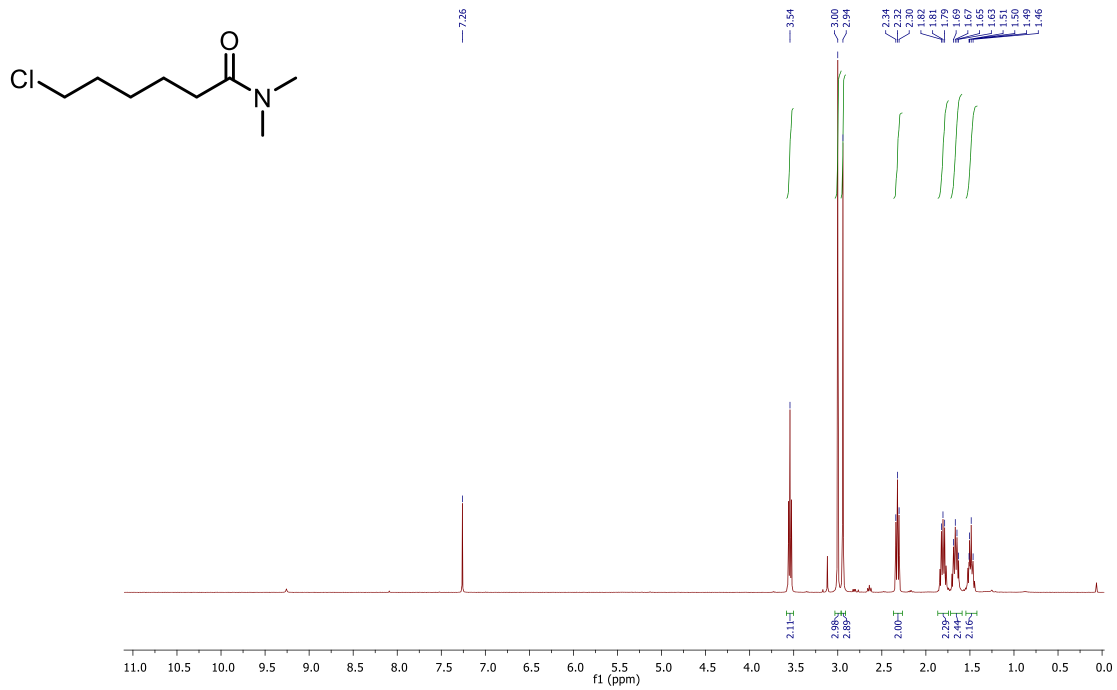

[1]H
$CDCl_3$
400 MHz

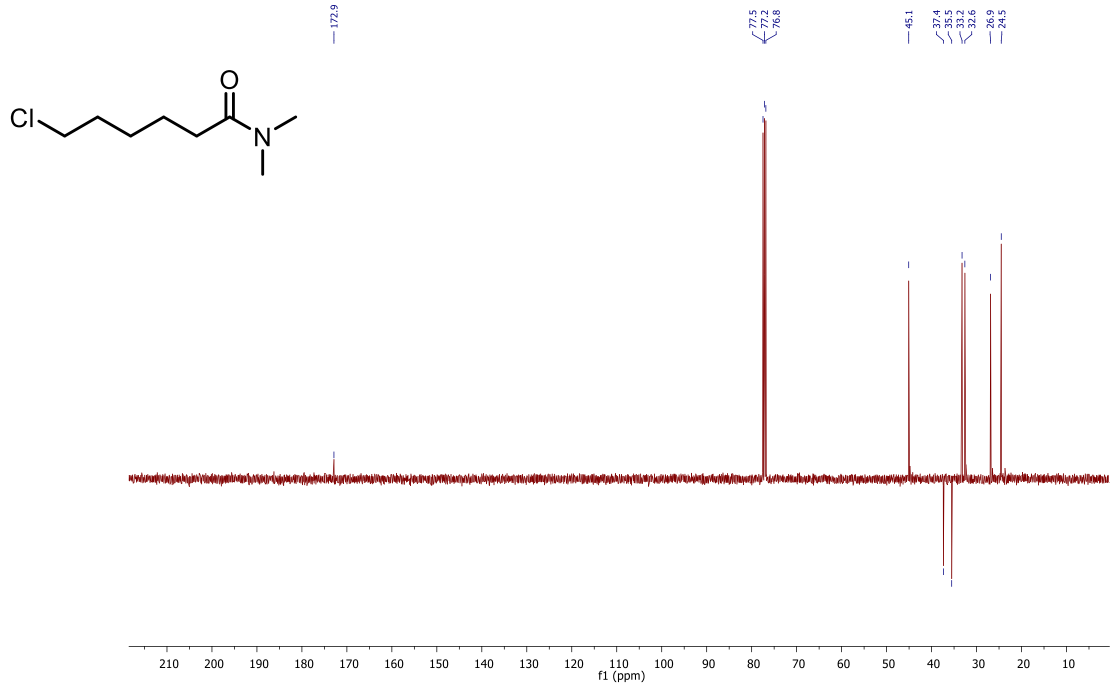

[13]C
$CDCl_3$
101 MHz

c) *N*-Benzyl-6-cyano-*N*-methylhexanamide (1n)

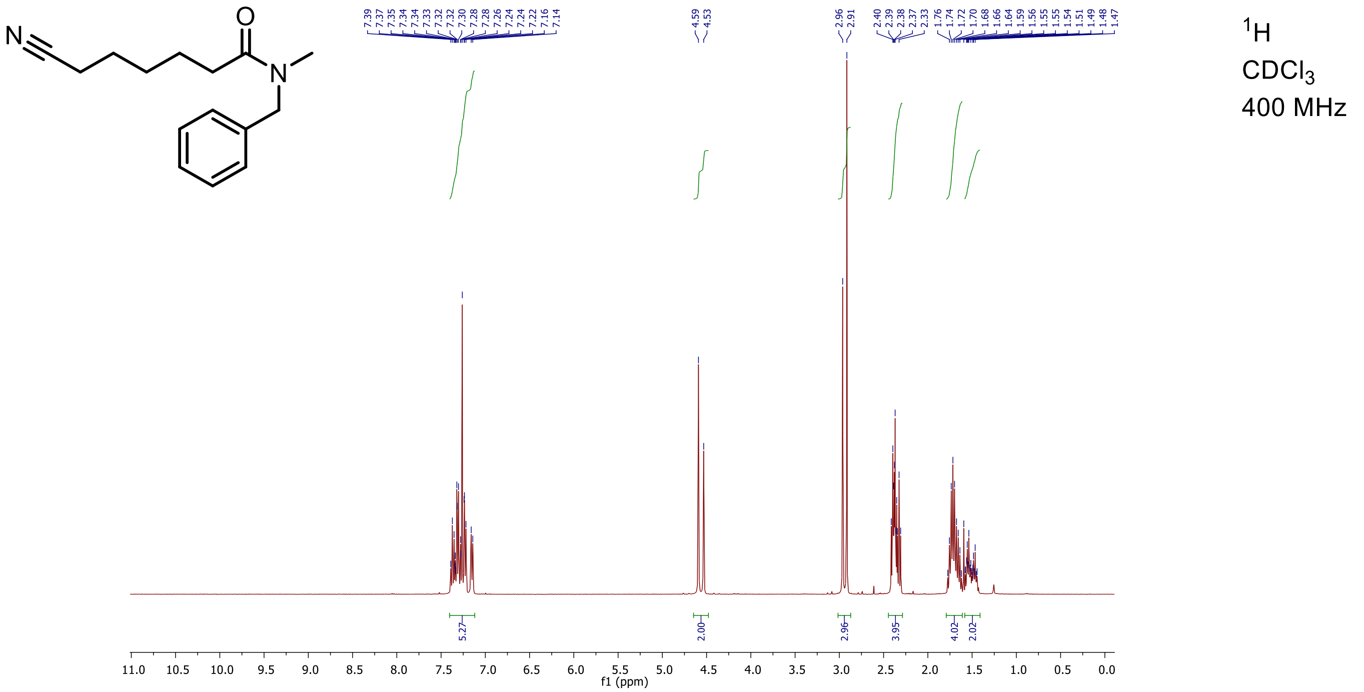

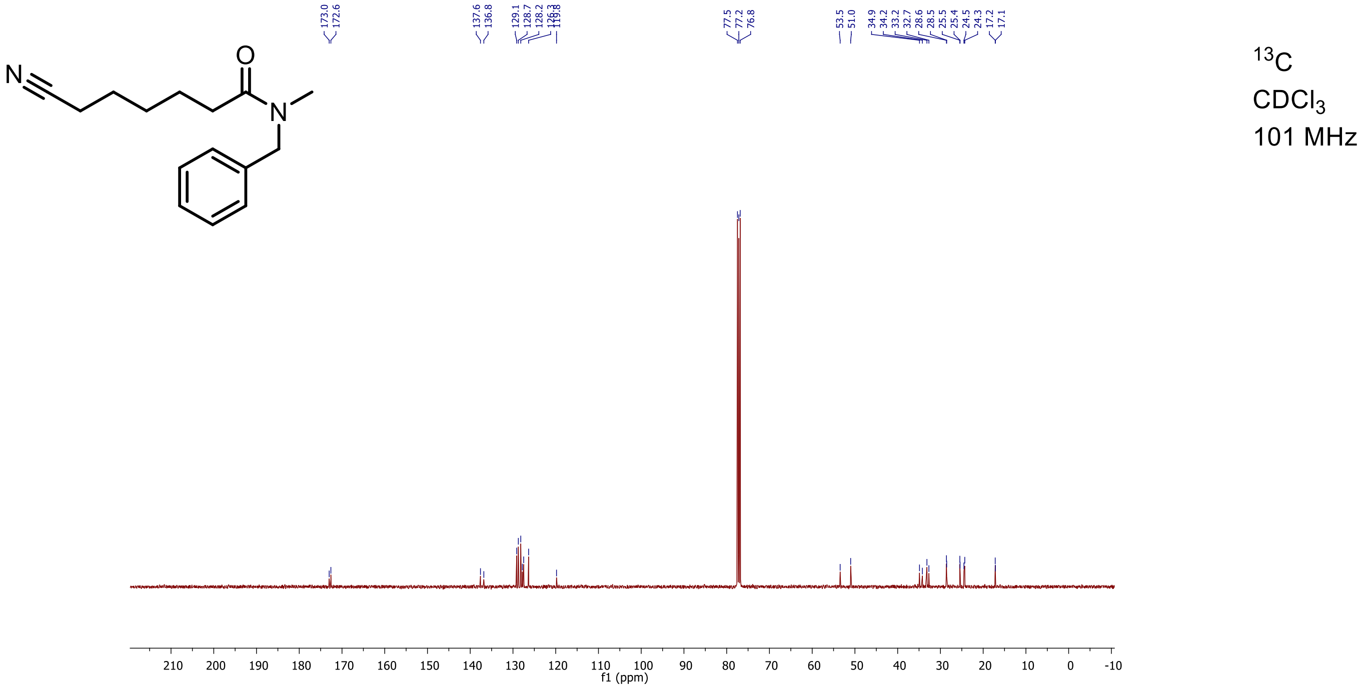

d) *N,N*-Diisopropylnonanamide (1t)

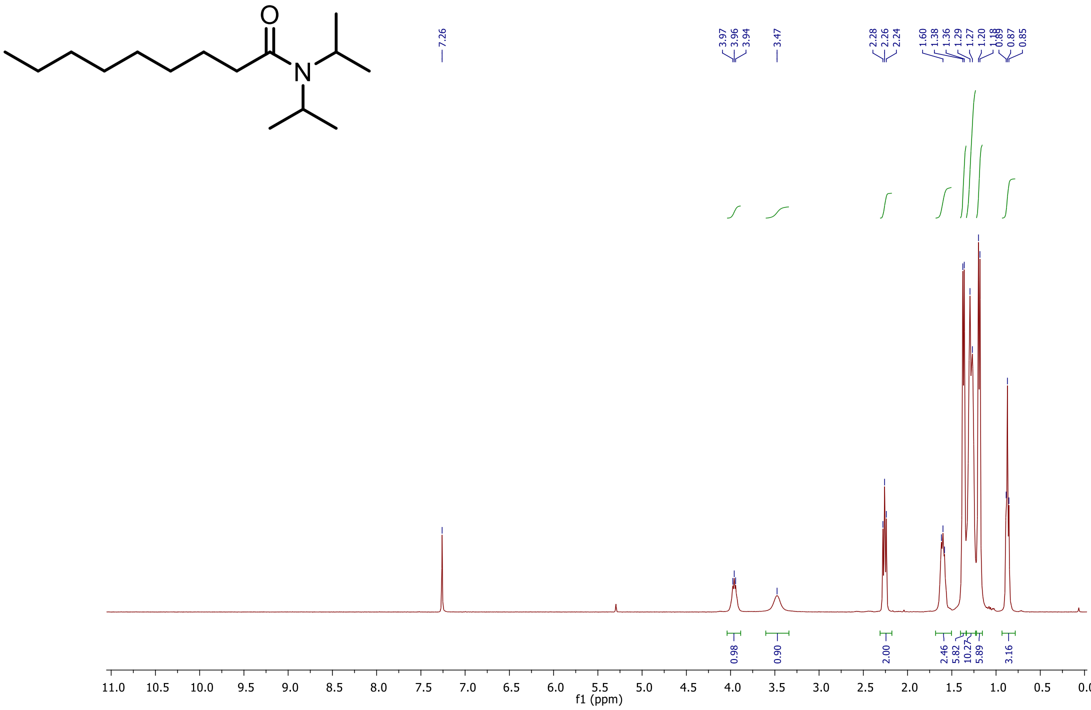

^{1}H
$CDCl_3$
400 MHz

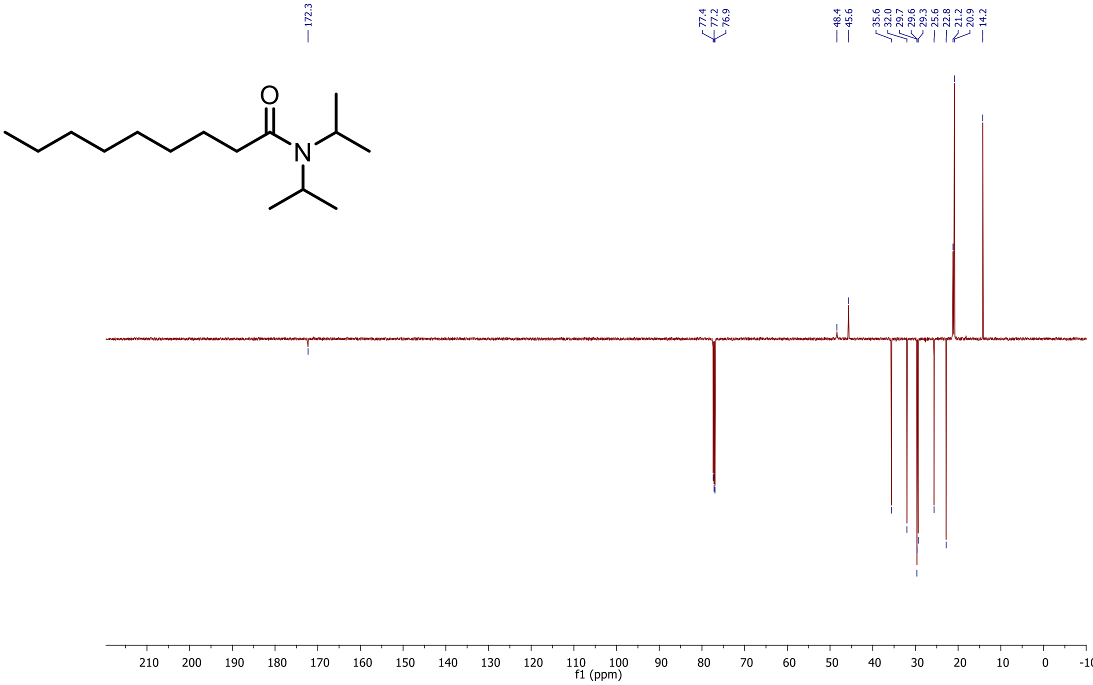

^{13}C
$CDCl_3$
151 MHz

4.2. NMR of Products

a) 4-Phenyl-1-(pyrrolidin-1-yl)butan-1-one-2-D (2a)

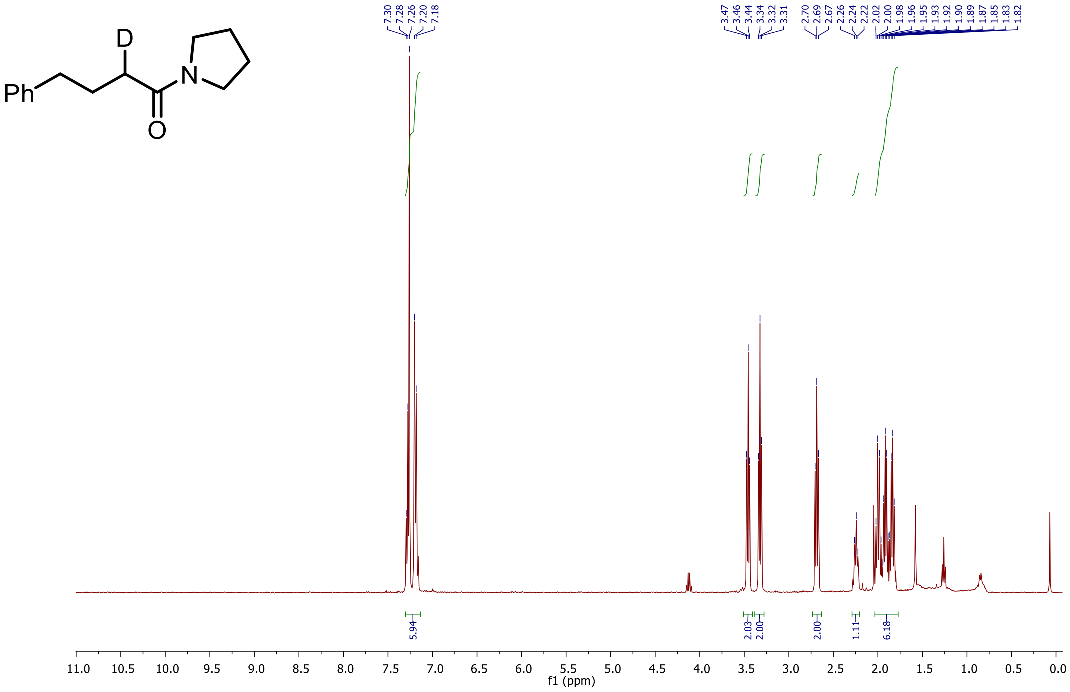

^{1}H
$CDCl_3$
400 MHz

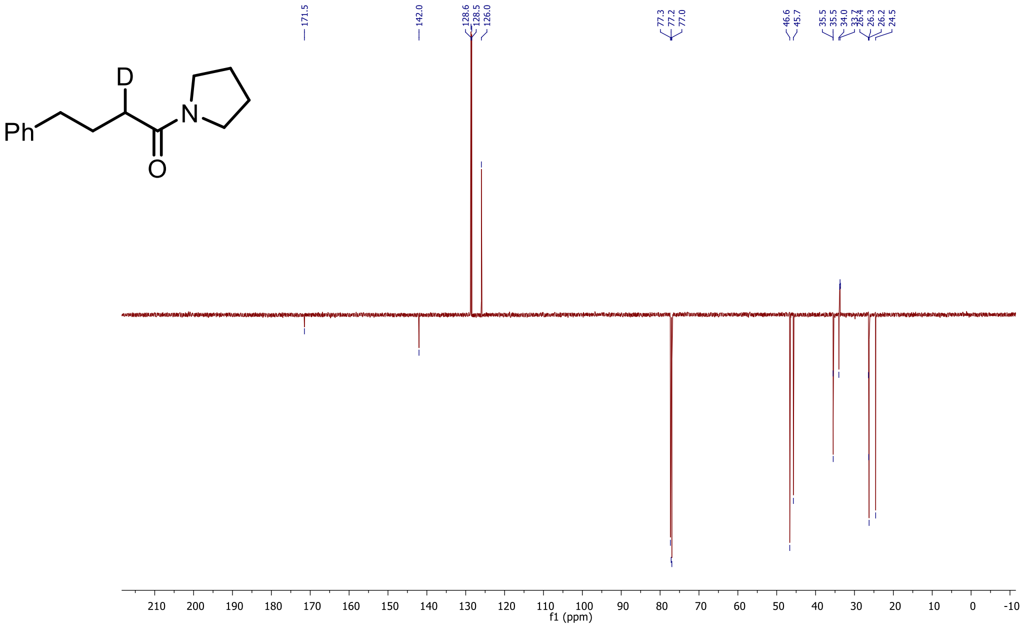

^{13}C
$CDCl_3$
176 MHz

b) *N,N*-dimethyl-4-phenylbutan-1-one-2-D (2b)

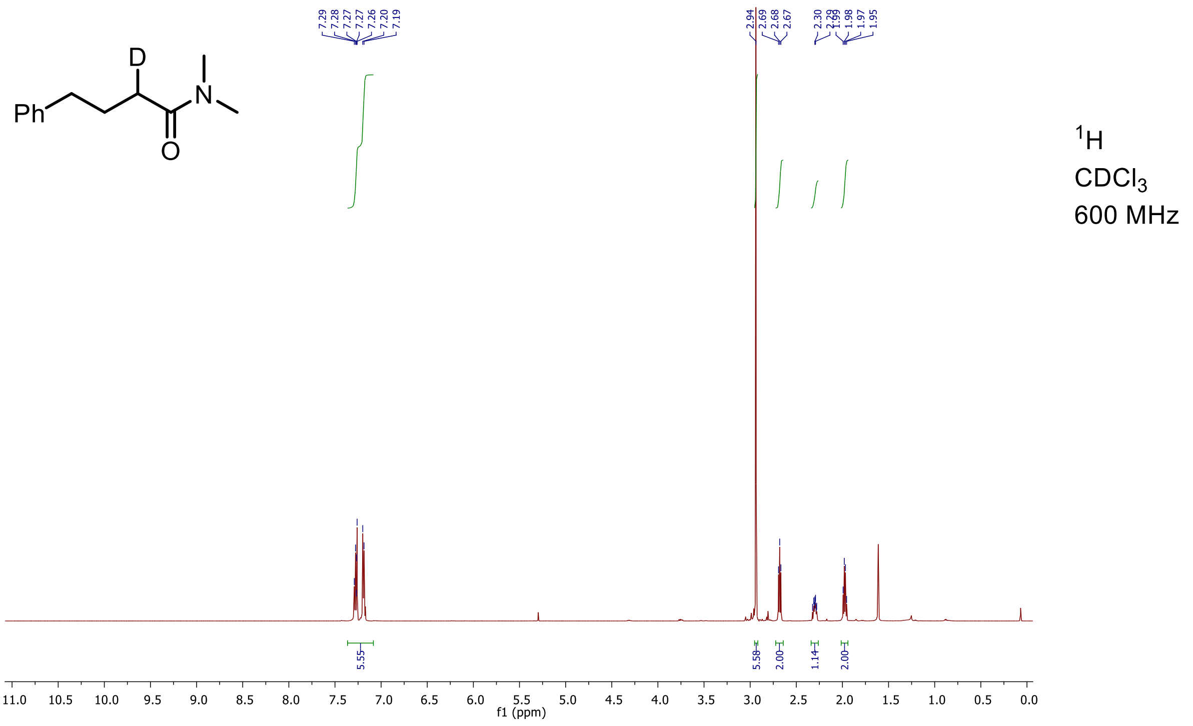

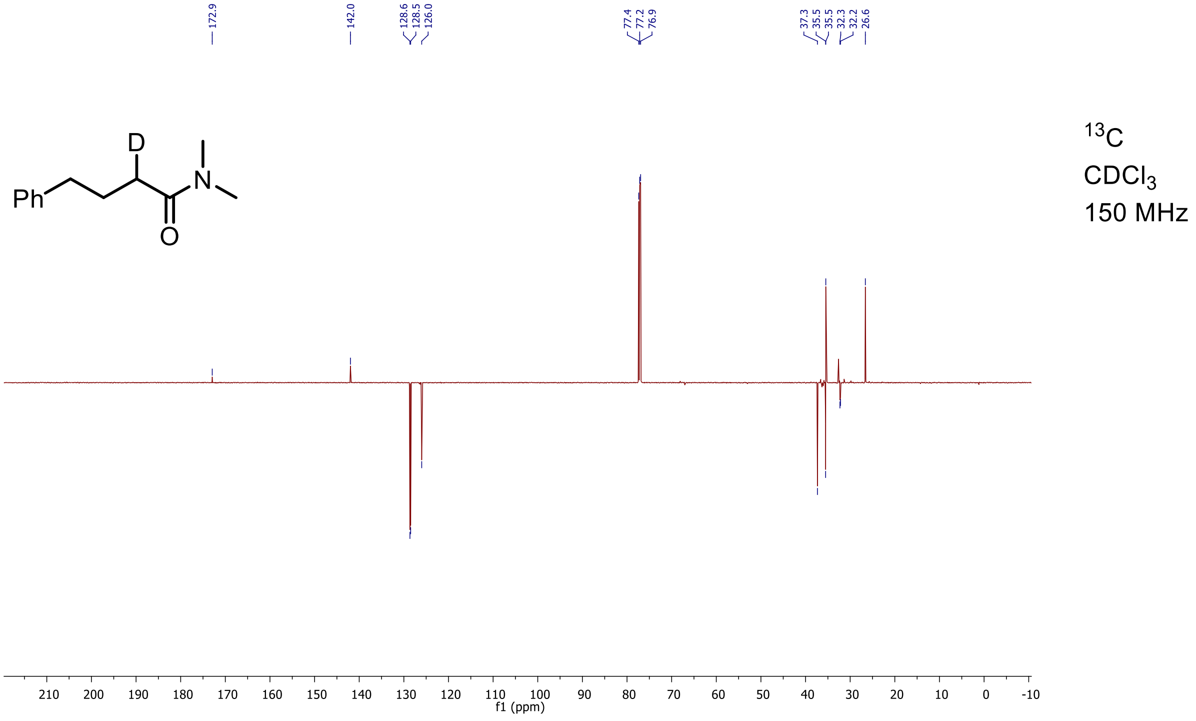

c) *N,N*-Diethylnonanamide-2-D (2c)

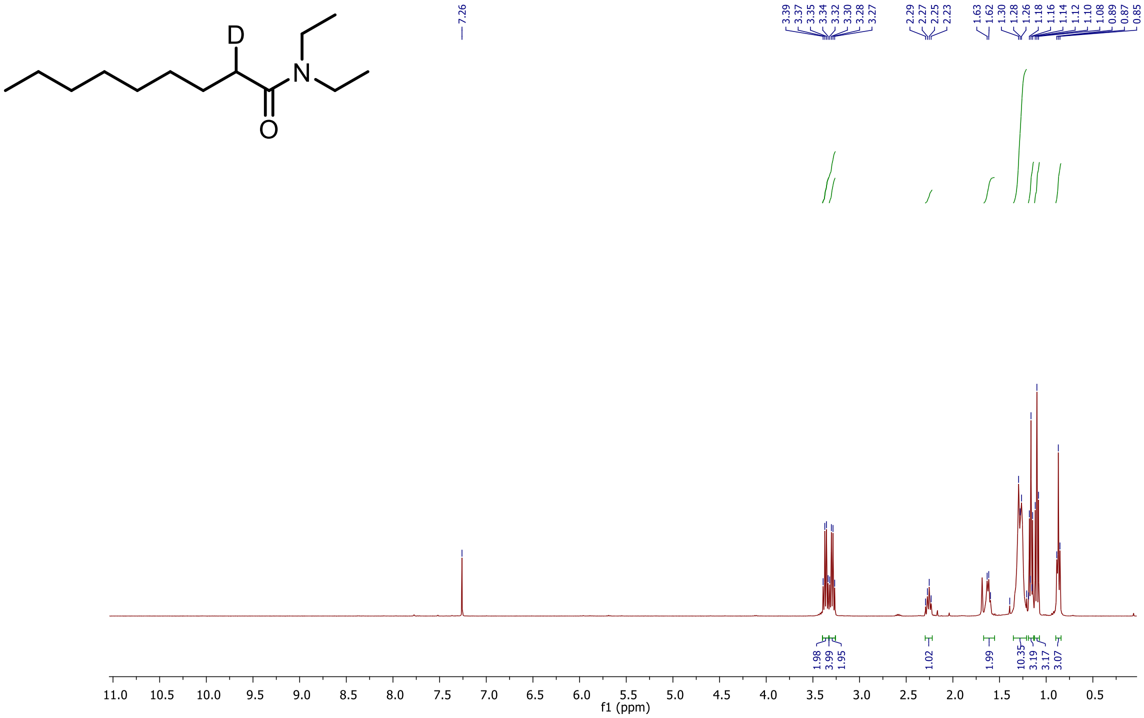

^{1}H
$CDCl_3$
400 MHz

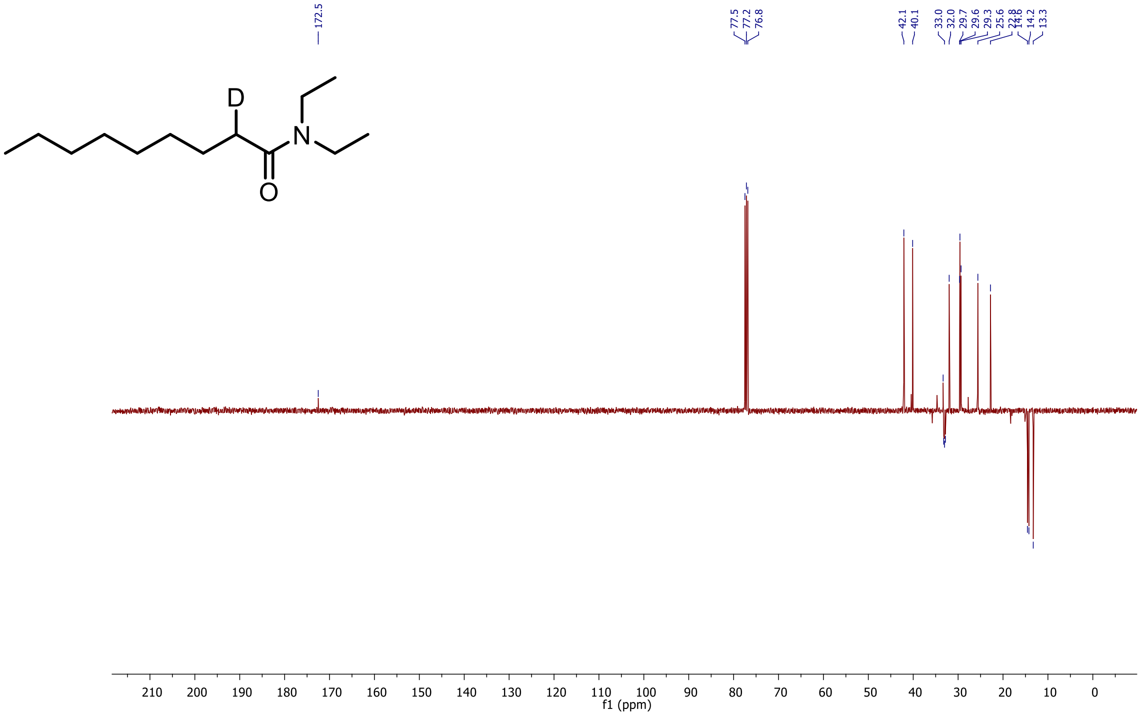

^{13}C
$CDCl_3$
101 MHz

d) *N,N*-Diallylnonanamide-2-D (2d)

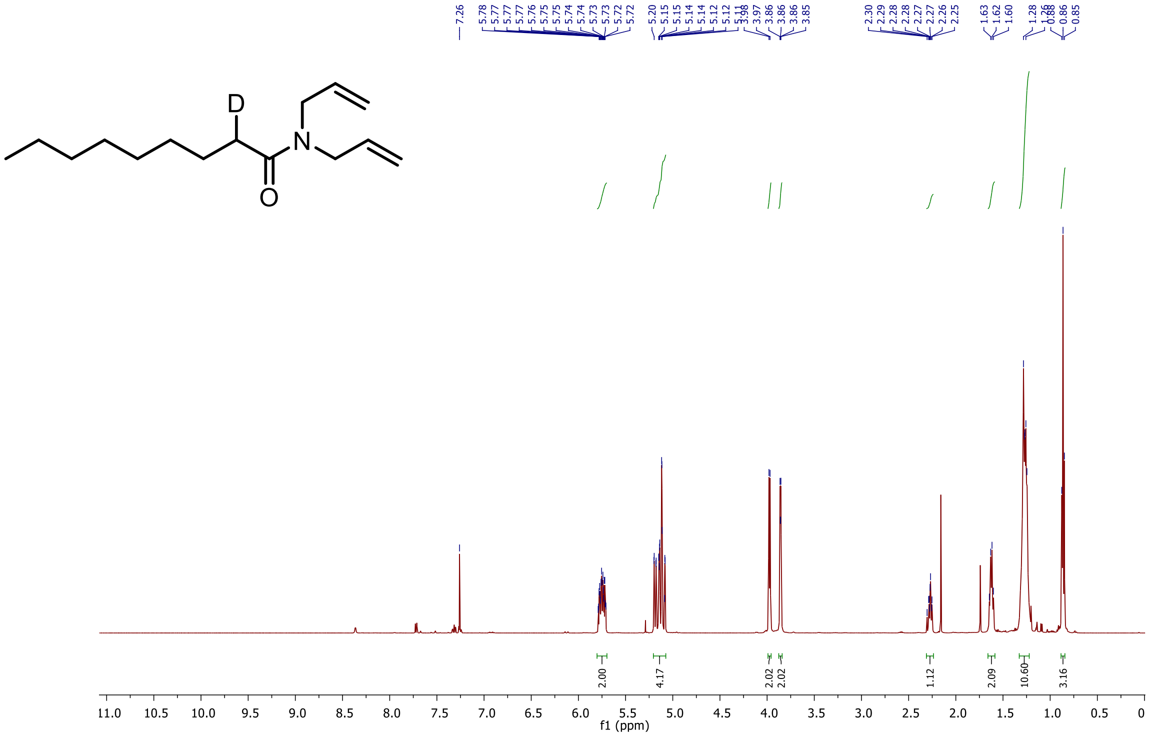

^{1}H
$CDCl_3$
500 MHz

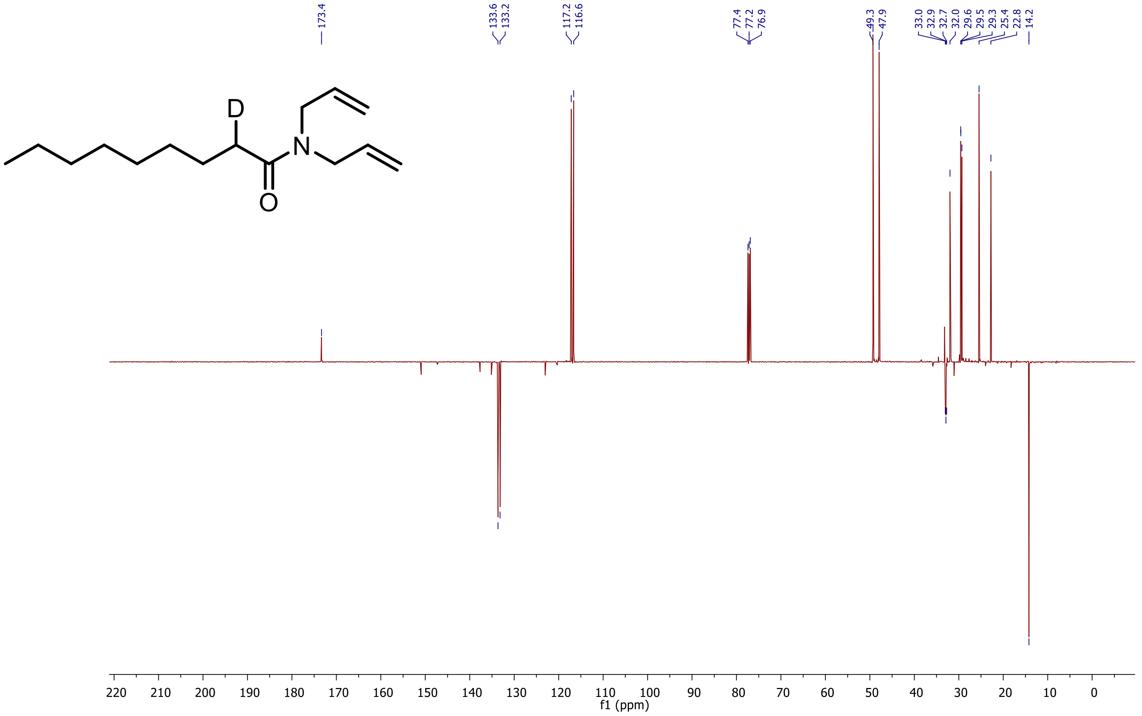

^{13}C
$CDCl_3$
126 MHz

e) ***N,N*-Dibenzylpentanamide-2-D (2e)**

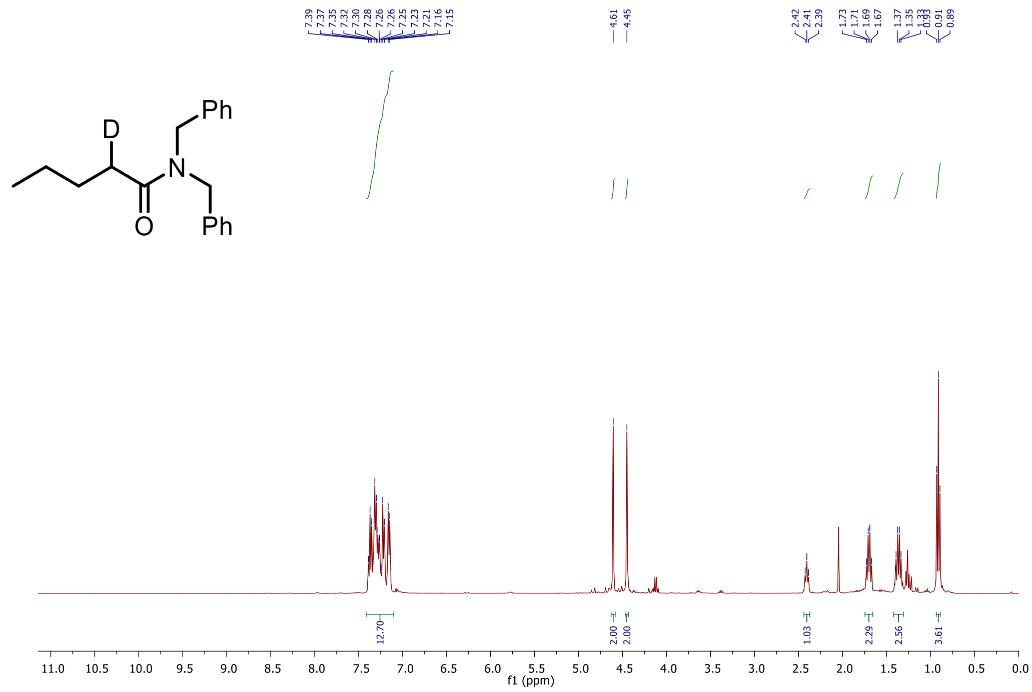

^{1}H
$CDCl_3$
400 MHz

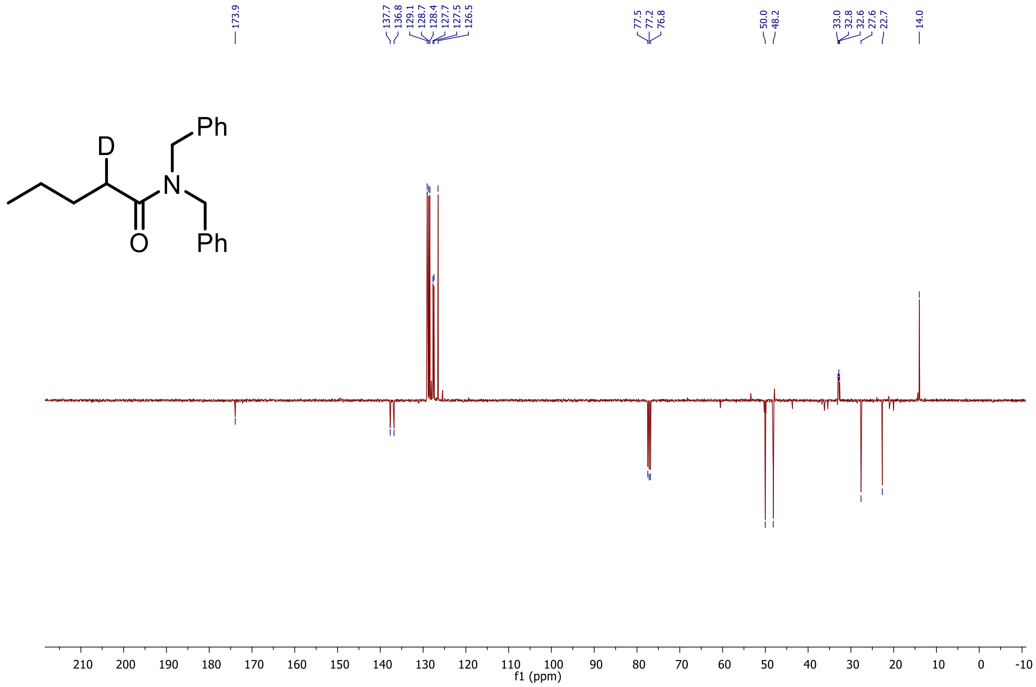

^{13}C
$CDCl_3$
101 MHz

f) 1-(Piperidin-1-yl)nonan-1-one-2-D (2f)

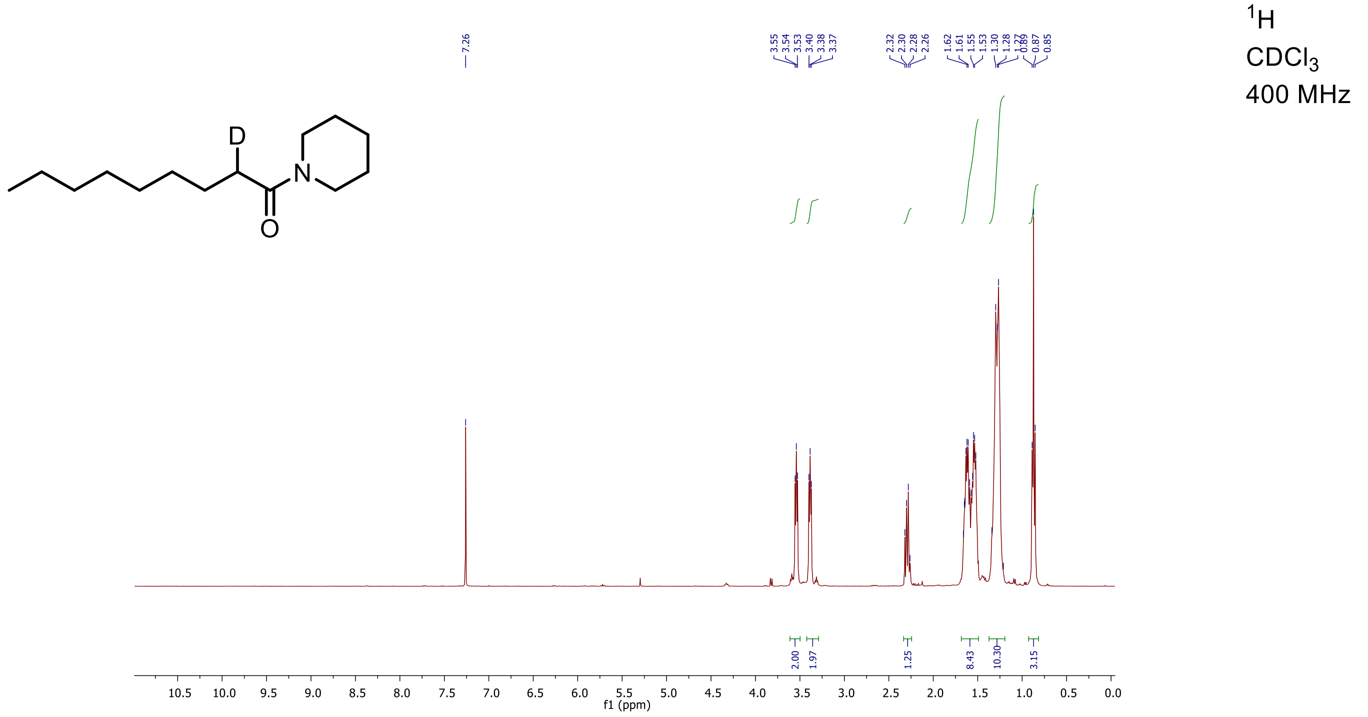

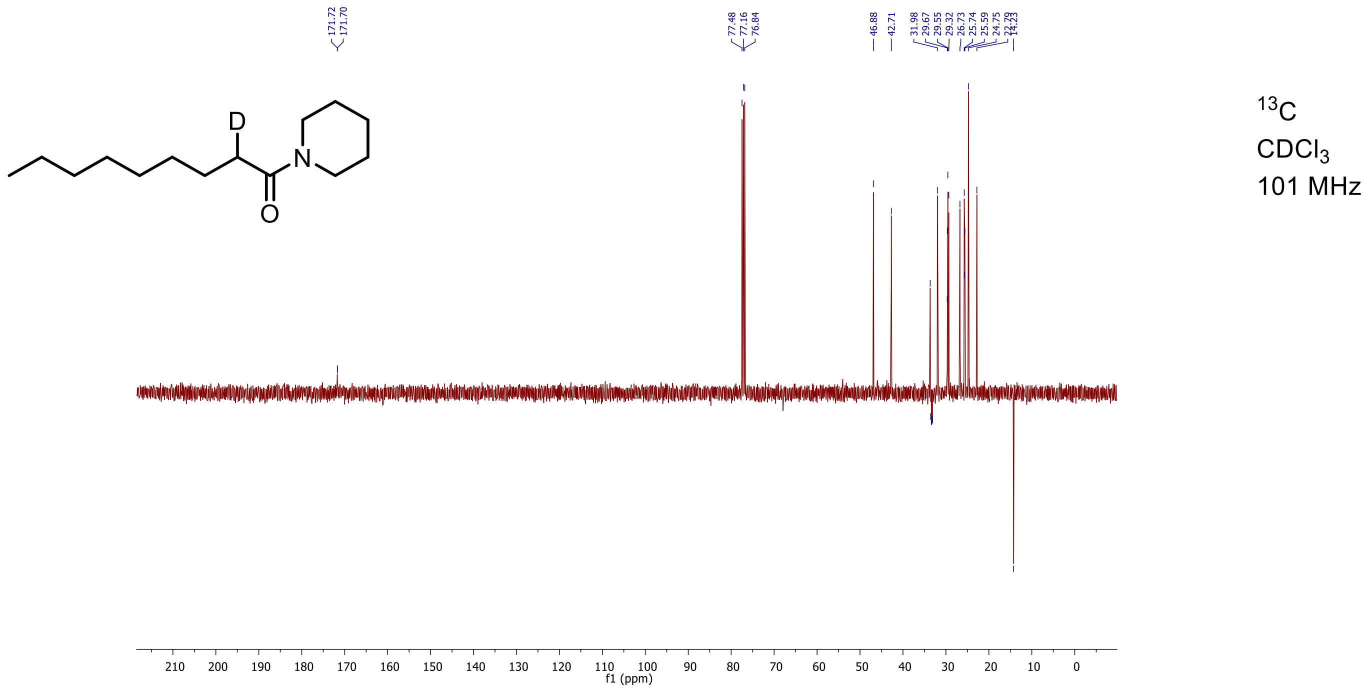

g) 1-(Azepan-1-yl)butan-1-one-2-D (2g)

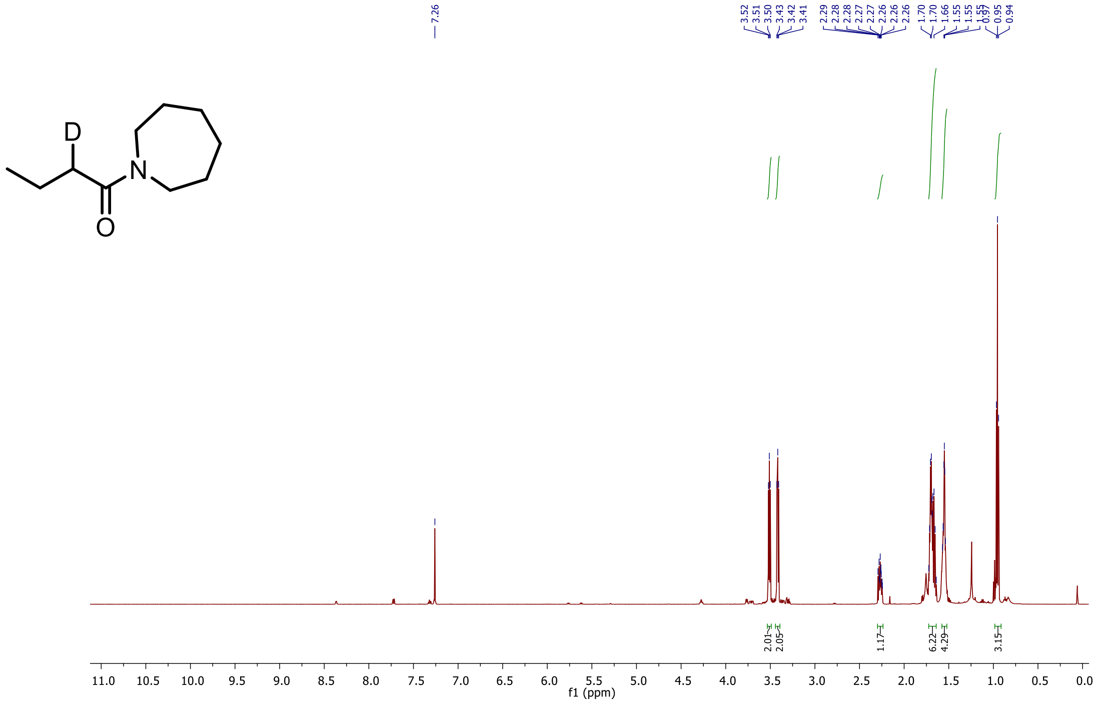

^{1}H
$CDCl_3$
400 MHz

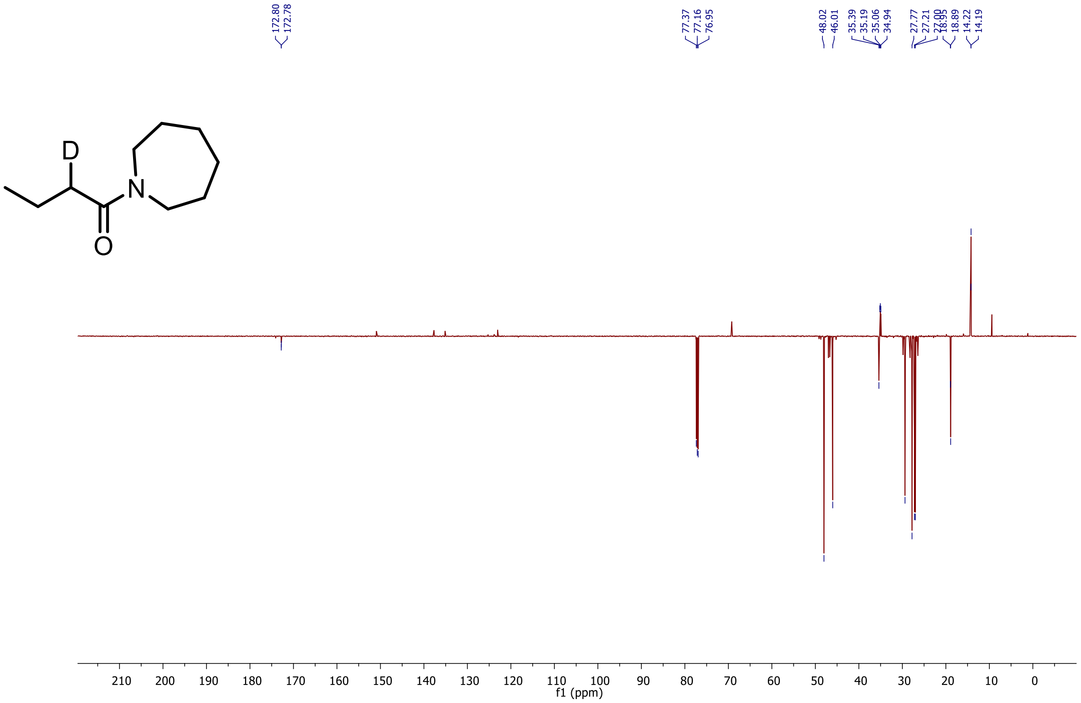

^{13}C
$CDCl_3$
151 MHz

h) 1-Methylazacyclotridecan-2-one-2-D (2h)

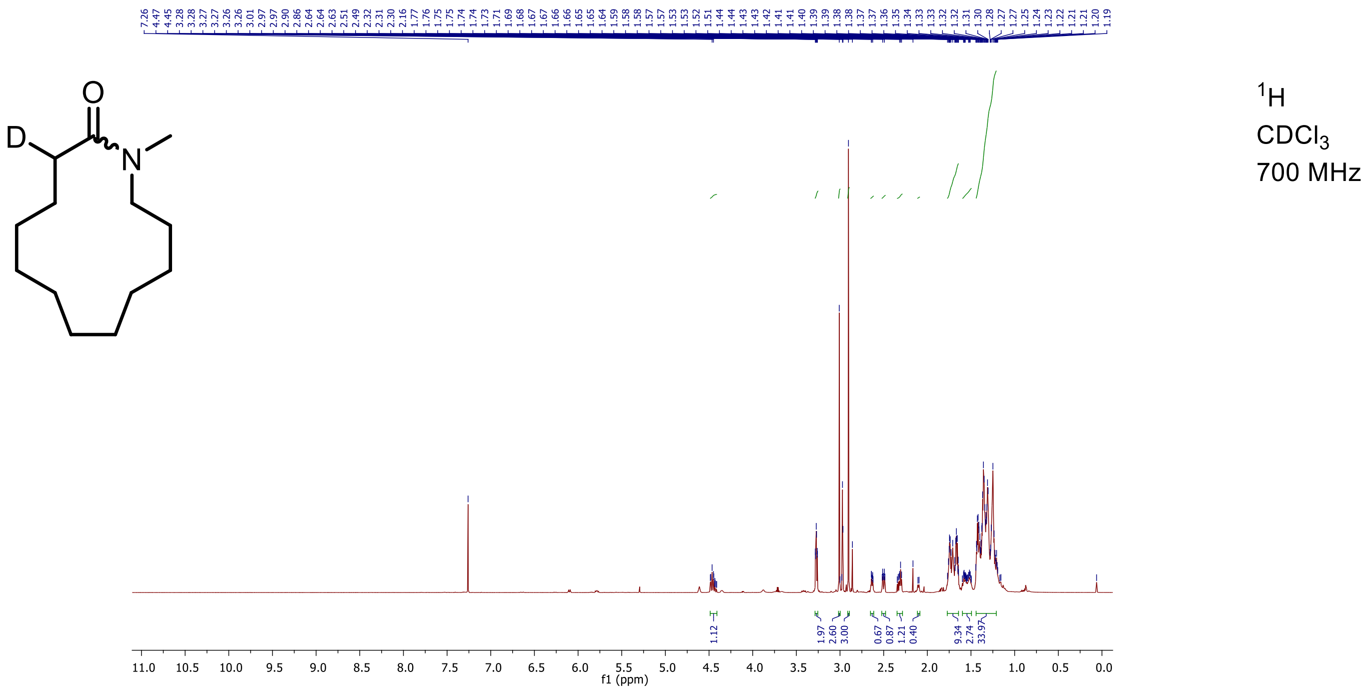

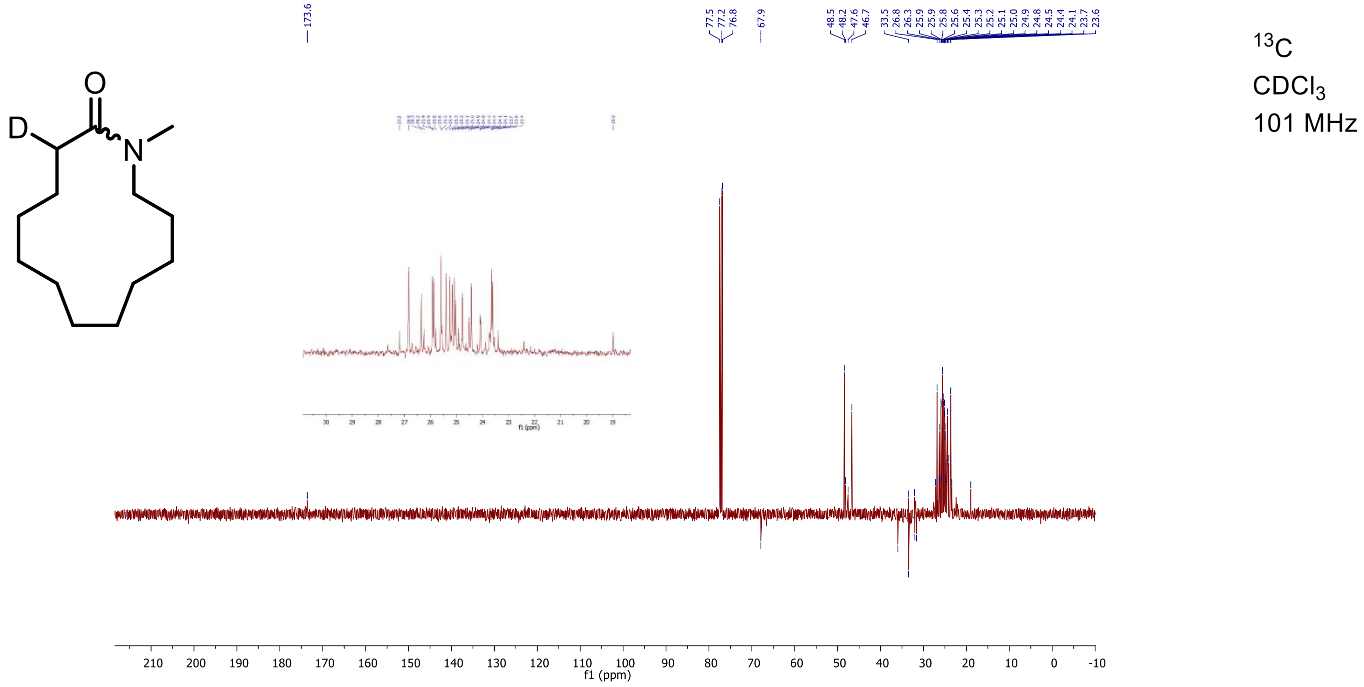

i) 3-Methyl-1-(pyrrolidin-1-yl)butan-1-one-2-D (2i)

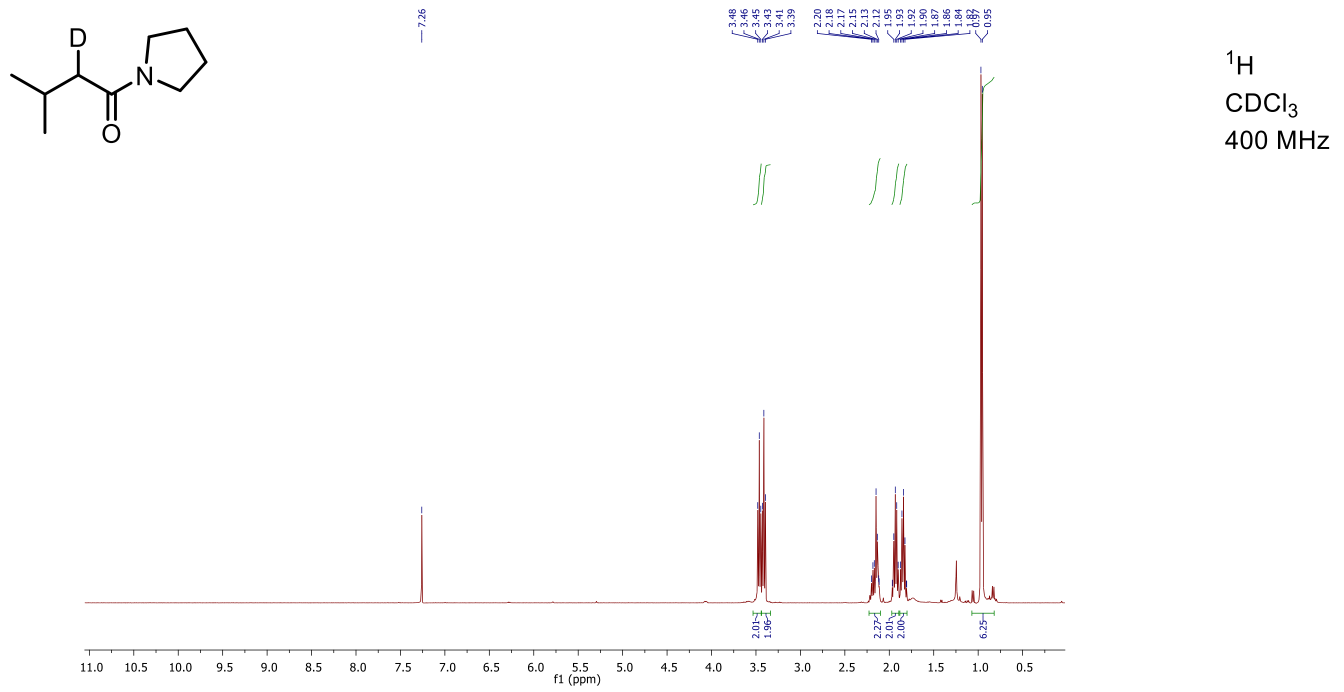

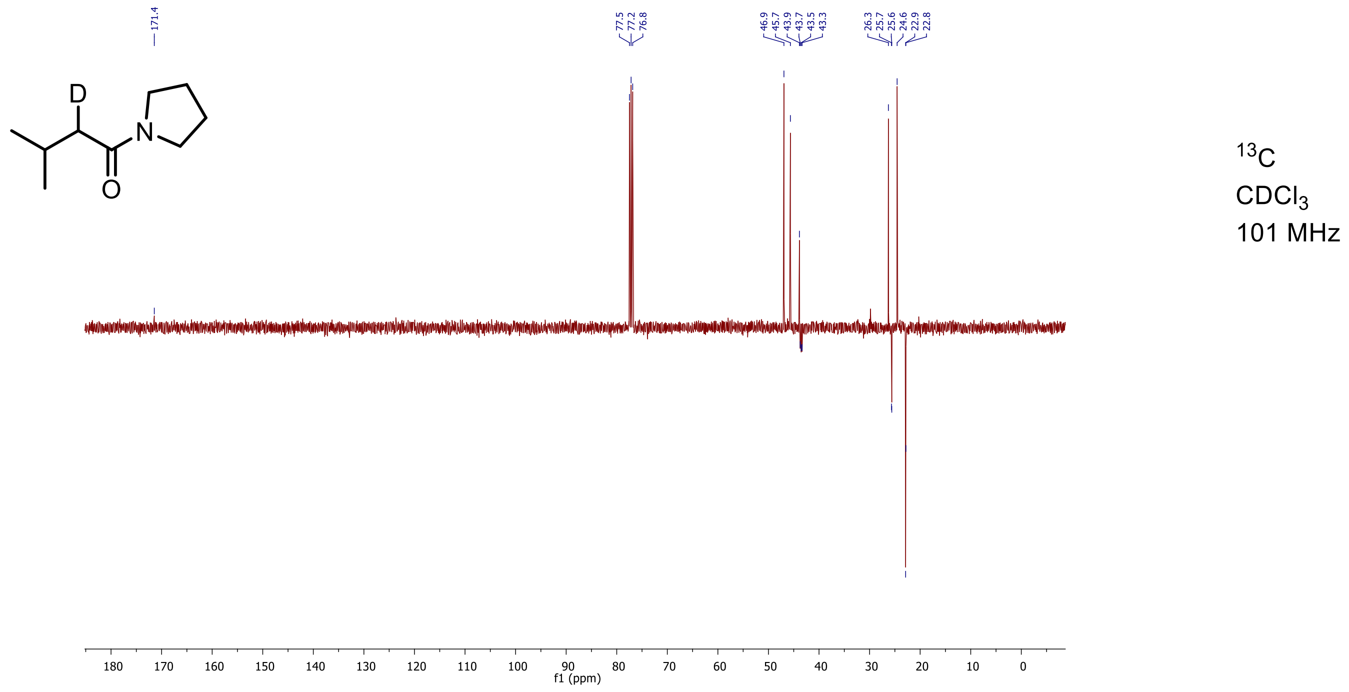

j) 1-(Pyrrolidin-1-yl)undec-10-yn-1-one-2-D (2j)

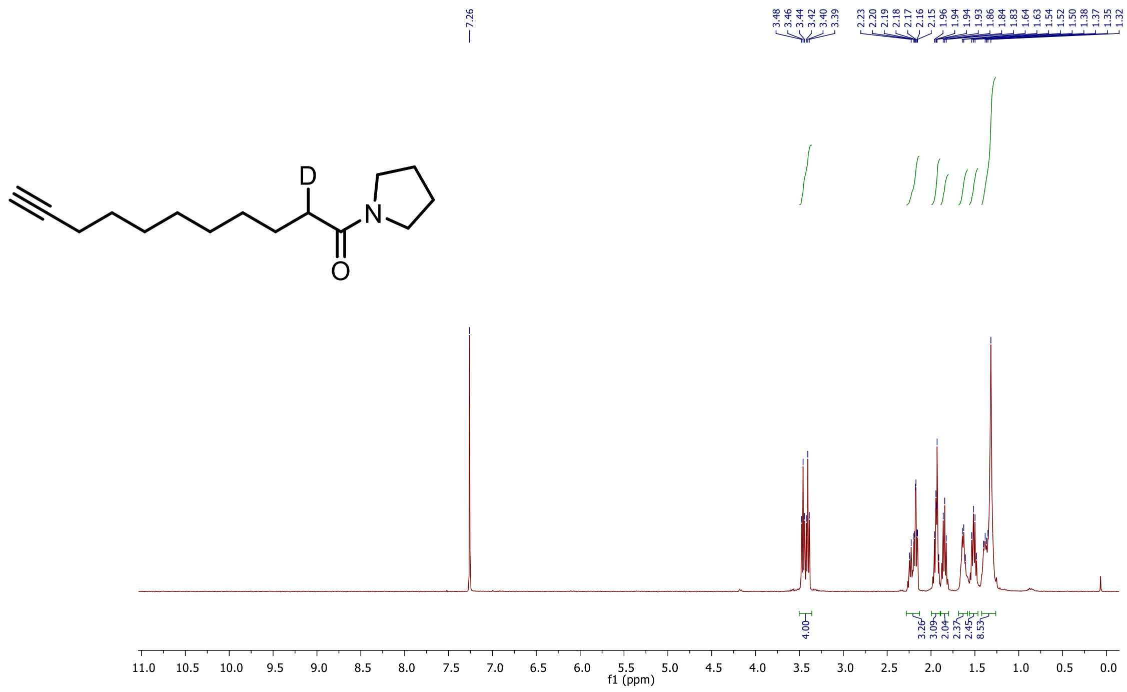

^{1}H
$CDCl_3$
400 MHz

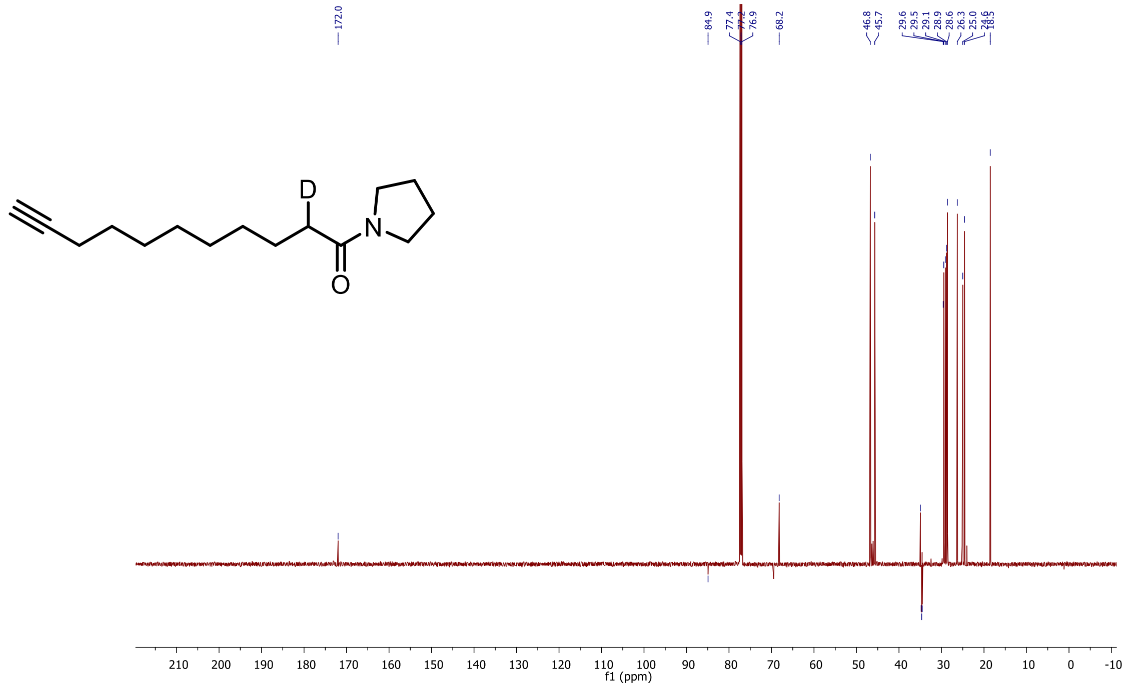

^{13}C
$CDCl_3$
151 MHz

k) *N,N*-Dimethylundec-10-enamide-2-D (2k)

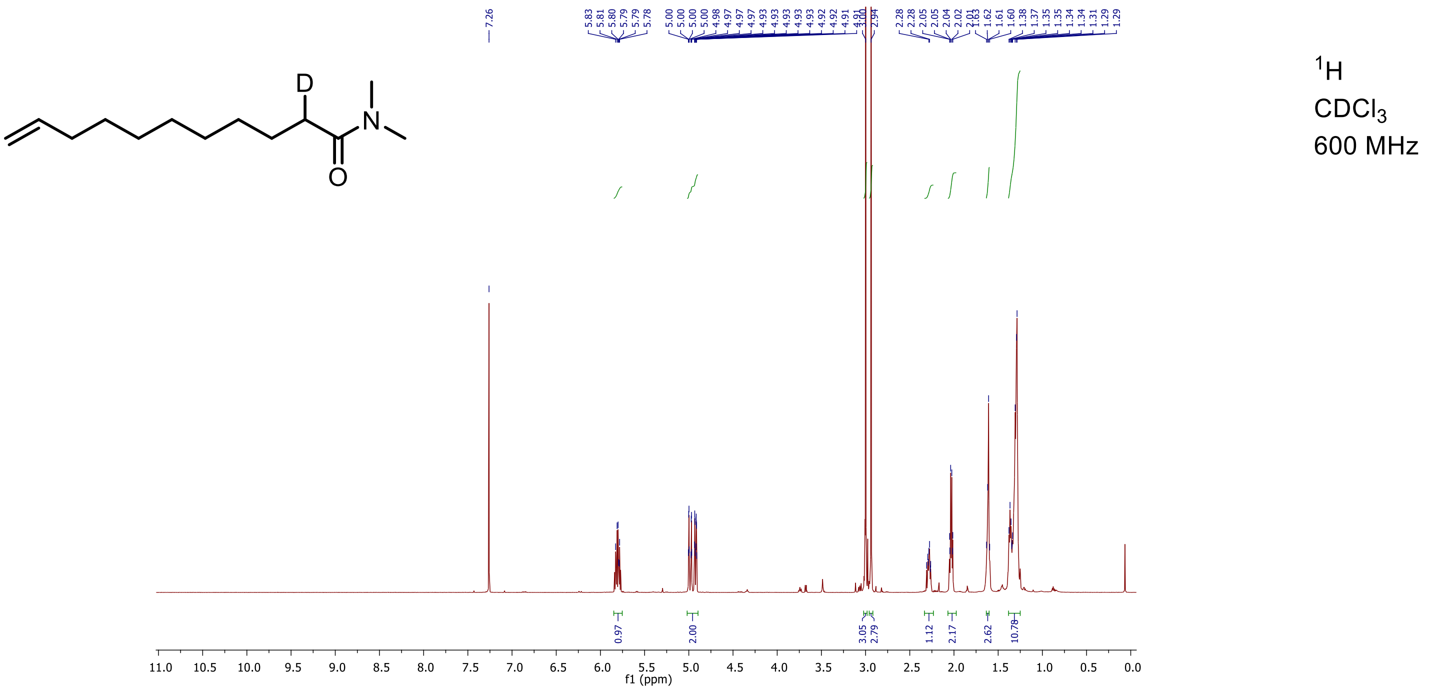

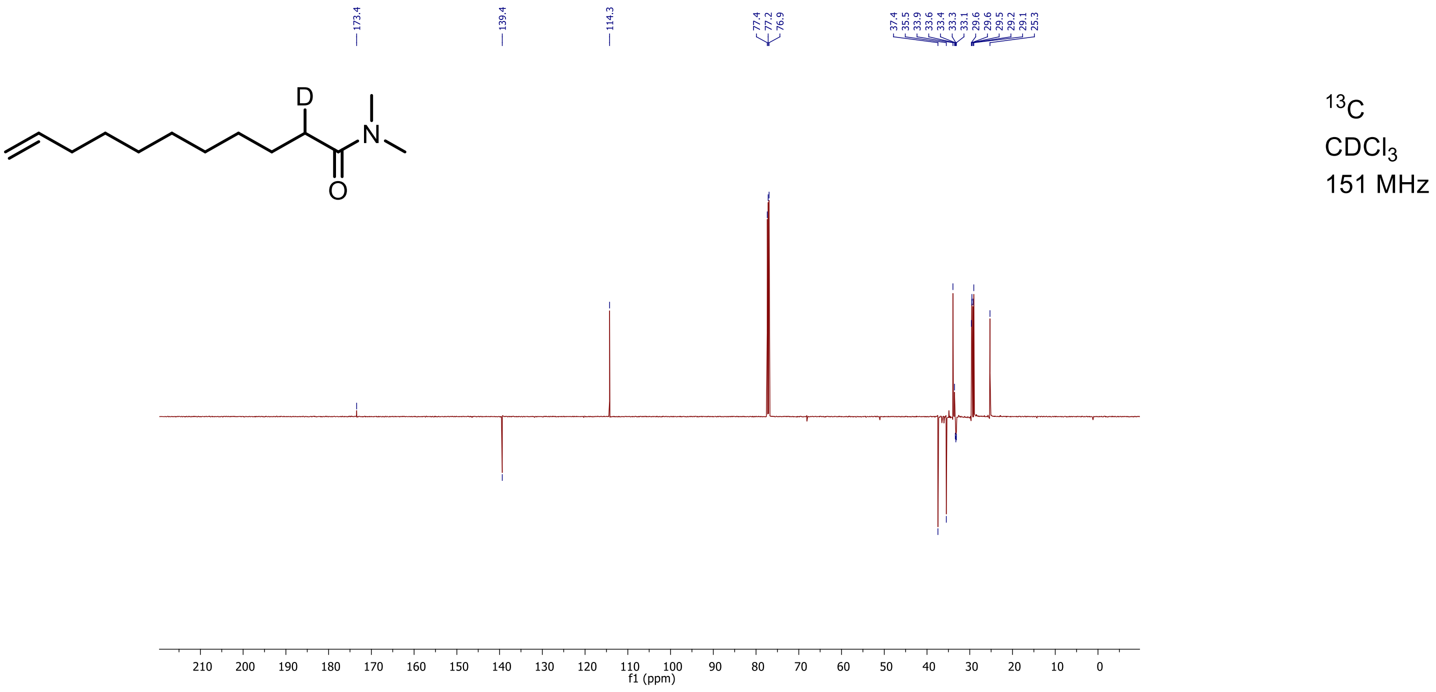

l) Methyl 9-oxo-9-(pyrrolidin-1-yl)nonanoate-2-D (2l)

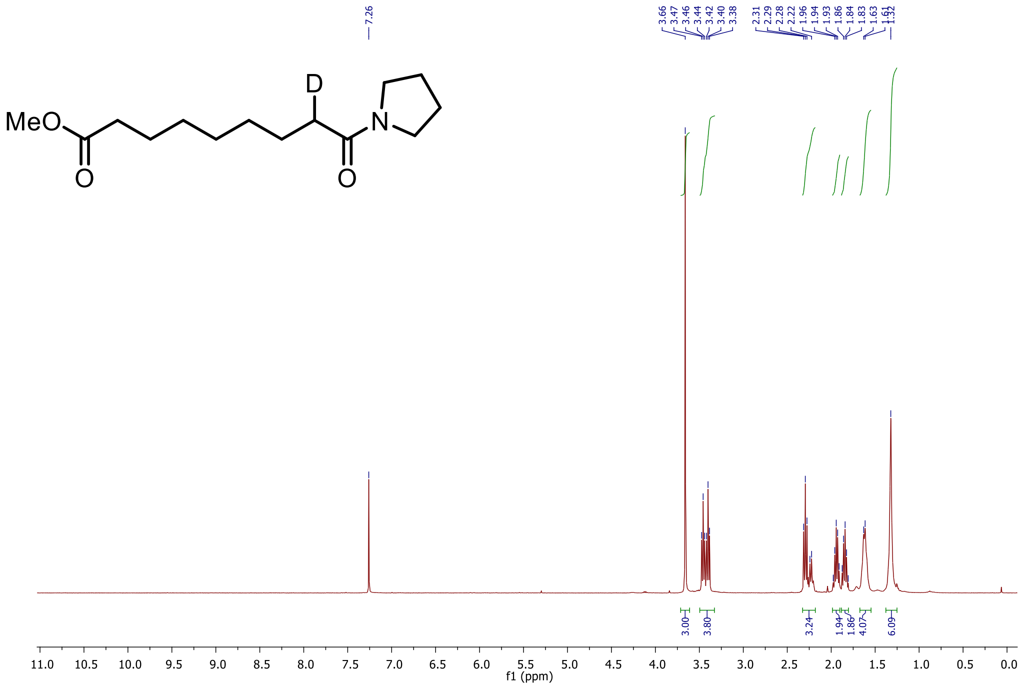

^{1}H
$CDCl_3$
400 MHz

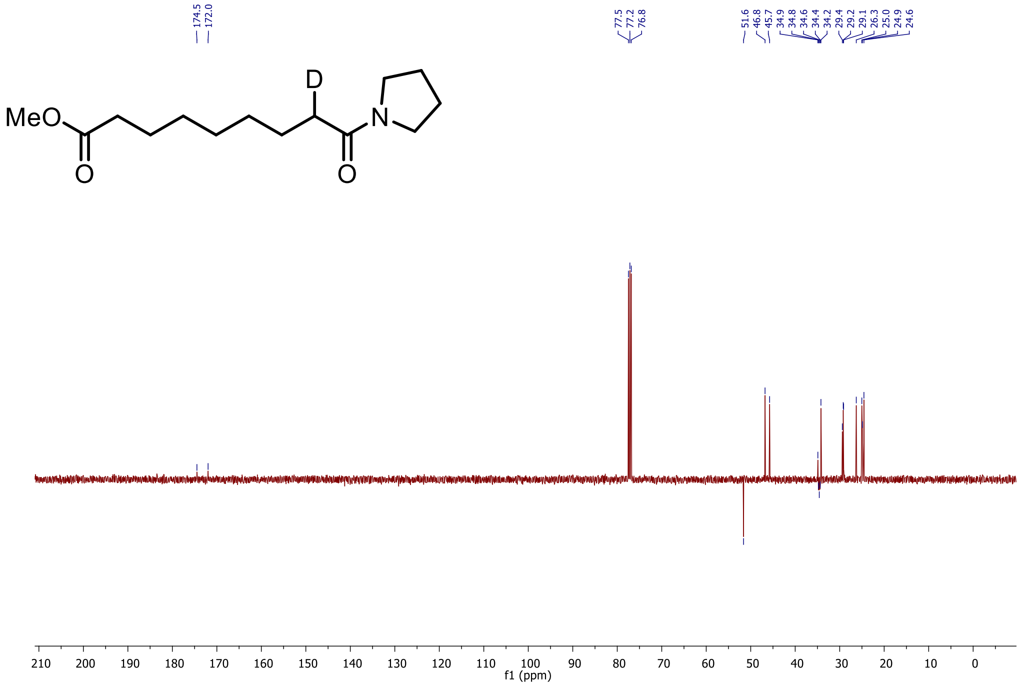

^{13}C
$CDCl_3$
101 MHz

m) *N*-Benzyl-*N*-methyl-10-oxoundecanamide-2-D (2m)

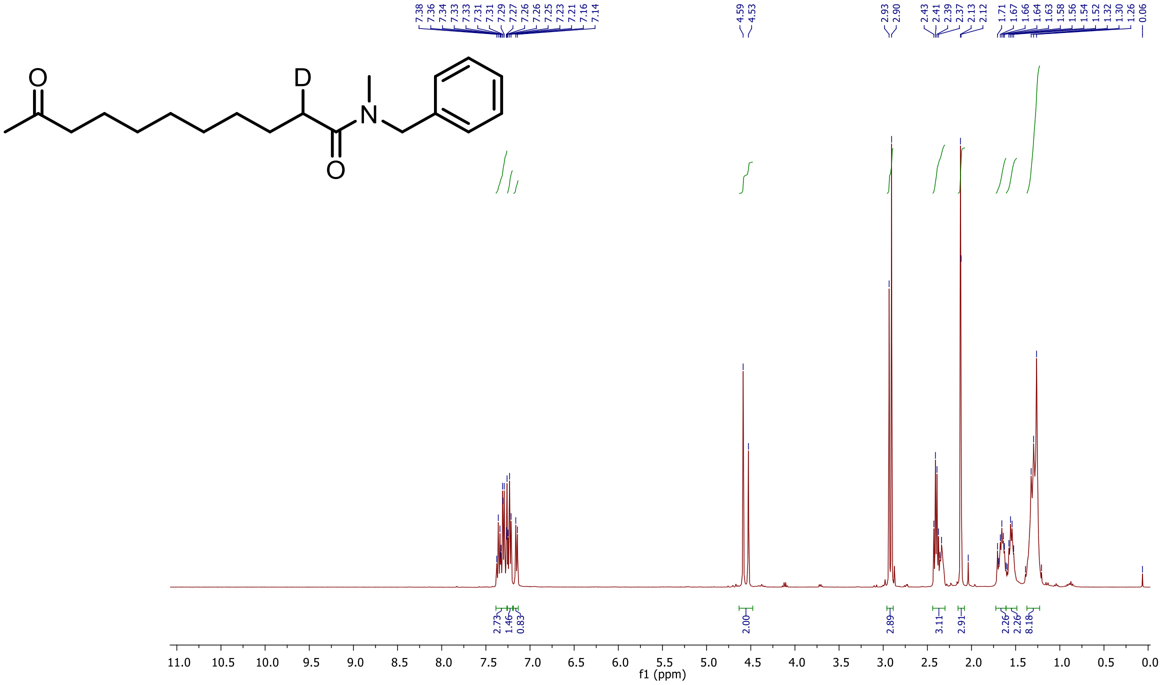

^{1}H
$CDCl_3$
400 MHz

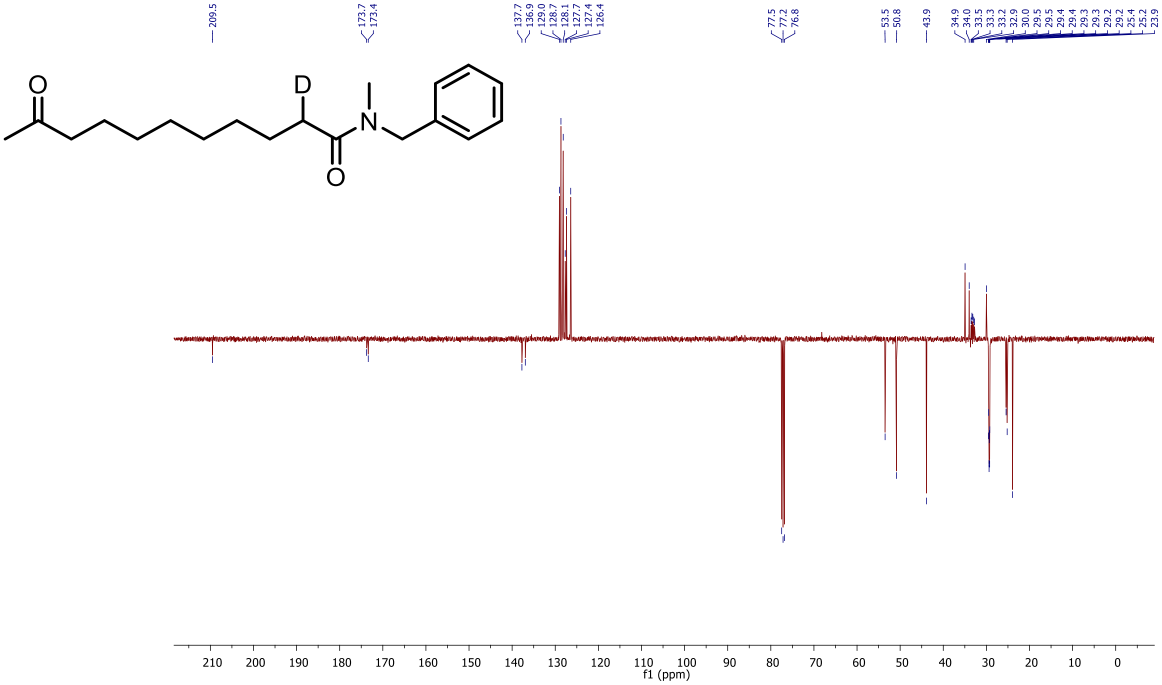

^{13}C
$CDCl_3$
101 MHz

n) *N*-Benzyl-6-cyano-*N*-methylhexanamide-2-D (2n)

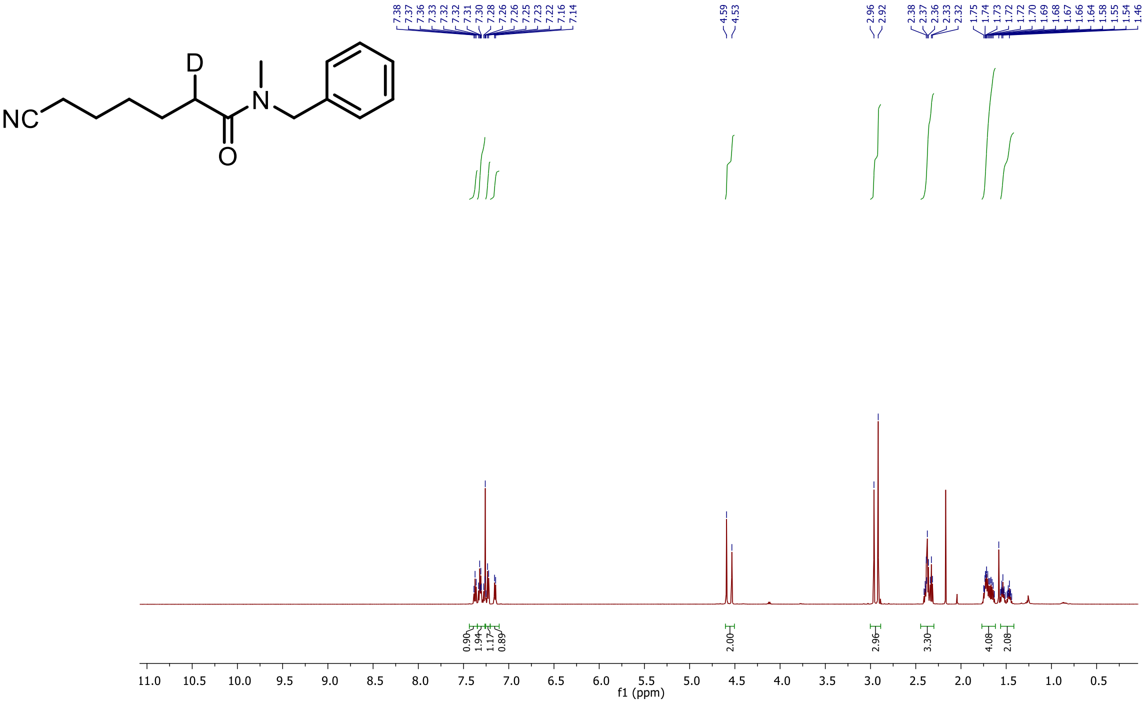

^{1}H
$CDCl_3$
600 MHz

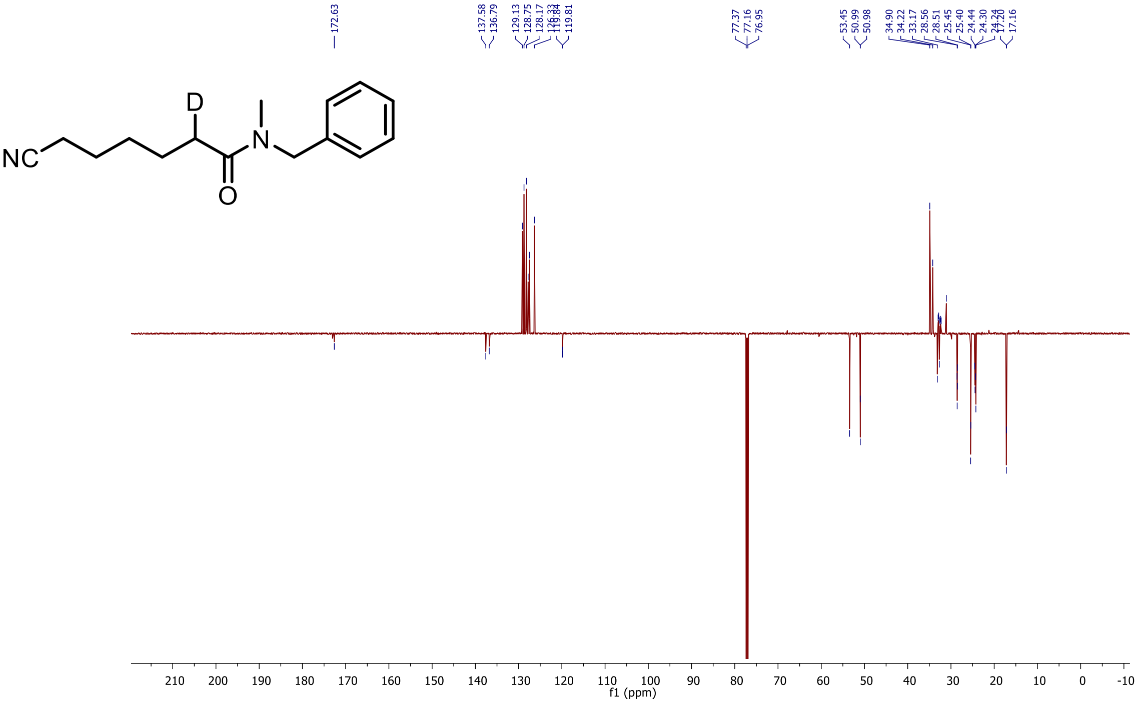

^{13}C
$CDCl_3$
151 MHz

o) 3-(4-Bromophenyl)-*N,N*-dimethylpropanamide-2-D (2o)

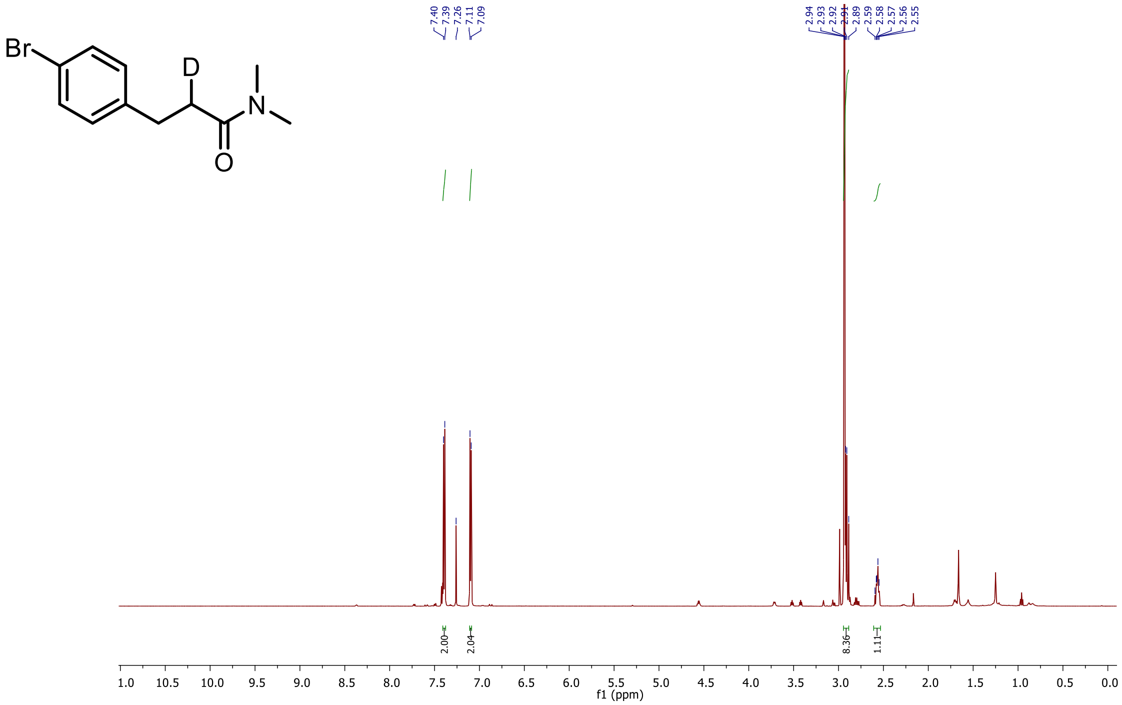

^{1}H
$CDCl_3$
600 MHz

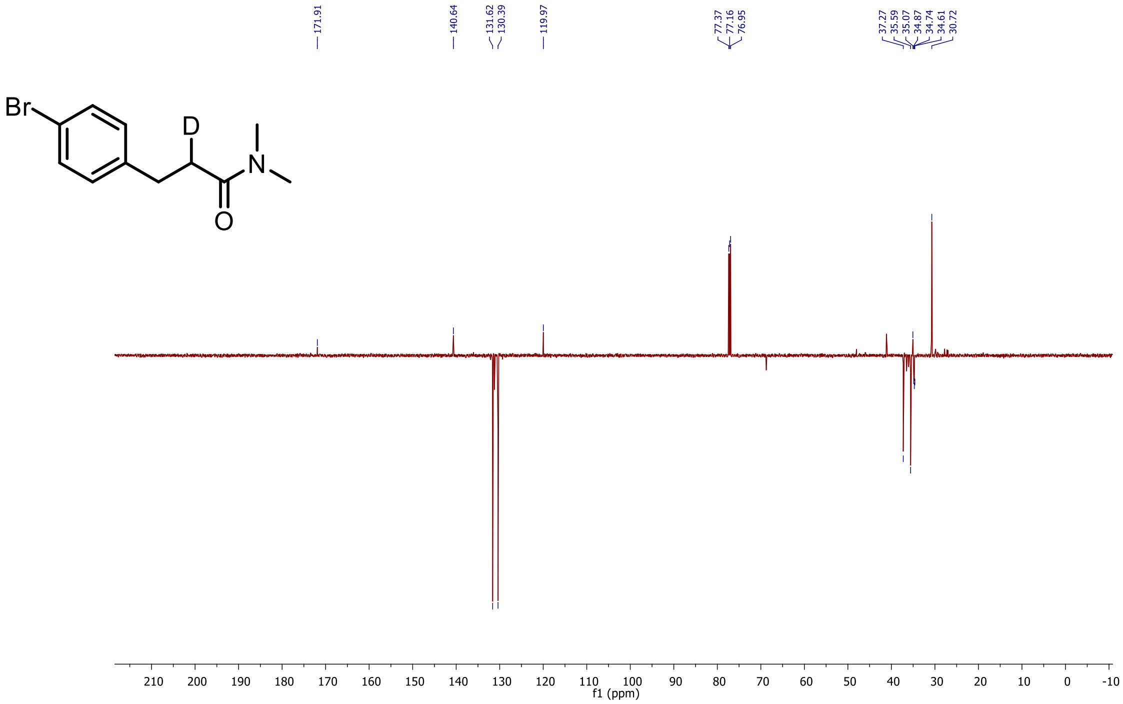

^{13}C
$CDCl_3$
151 MHz

p) 6-Chloro-*N*,*N*-dimethylhexanamide-2-D (2p)

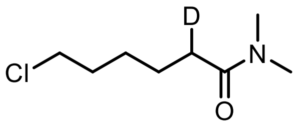

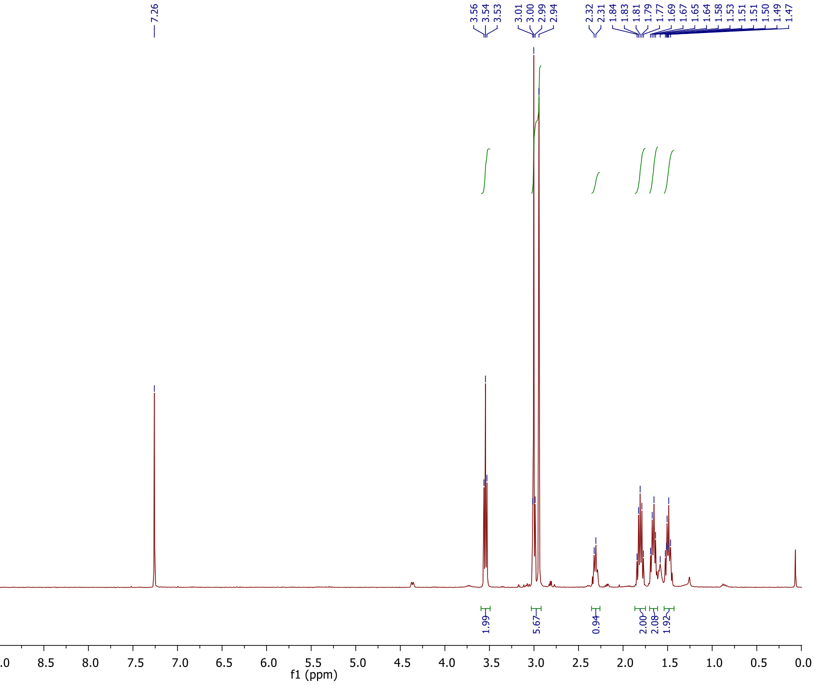

^{1}H
$CDCl_3$
400 MHz

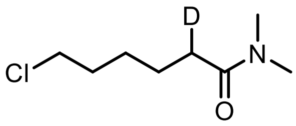

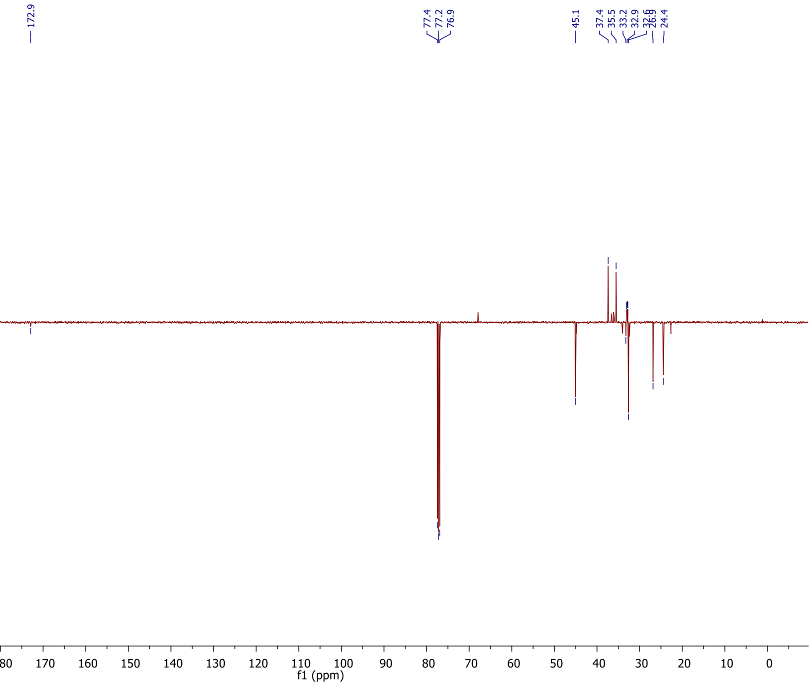

^{13}C
$CDCl_3$
101 MHz

q) 4,4,4-Trifluoro-1-(pyrrolidin-1-yl)butan-1-one-2-D (2q)

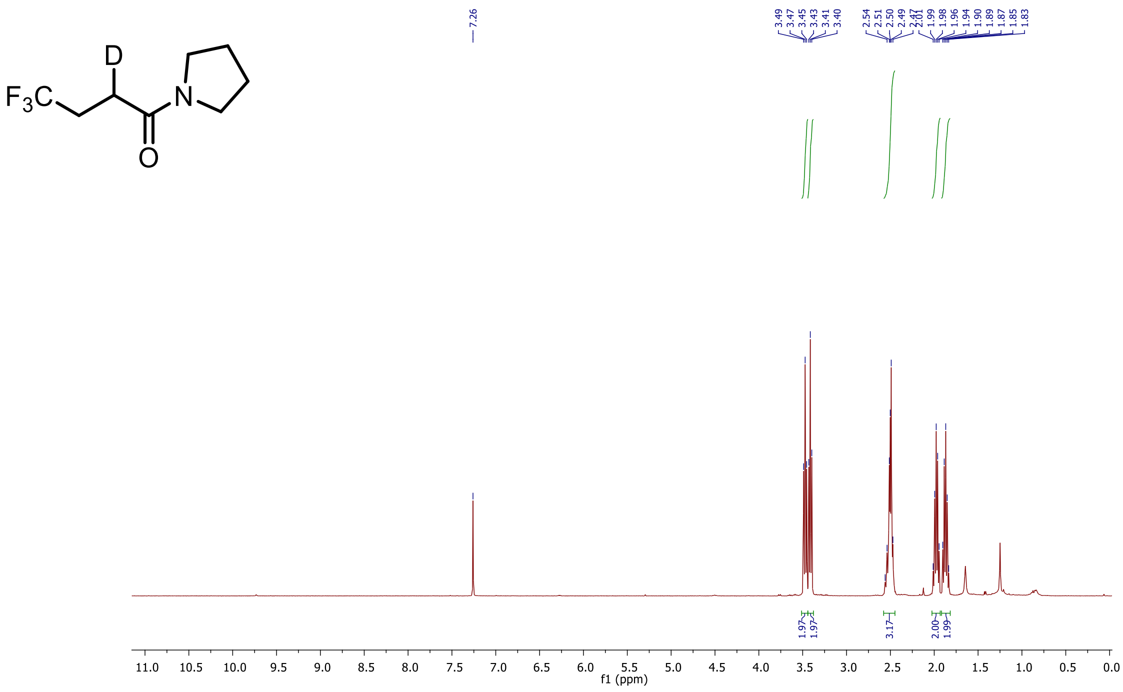

^{1}H
$CDCl_3$
400 MHz

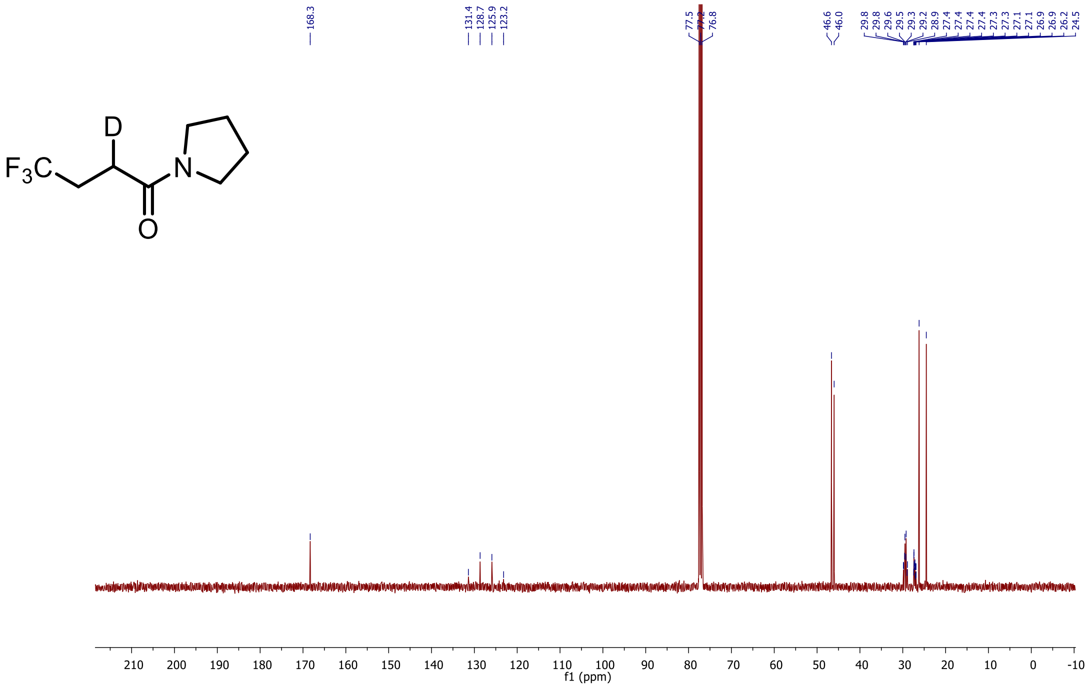

^{13}C
$CDCl_3$
101 MHz

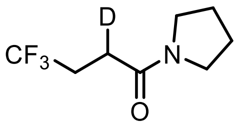

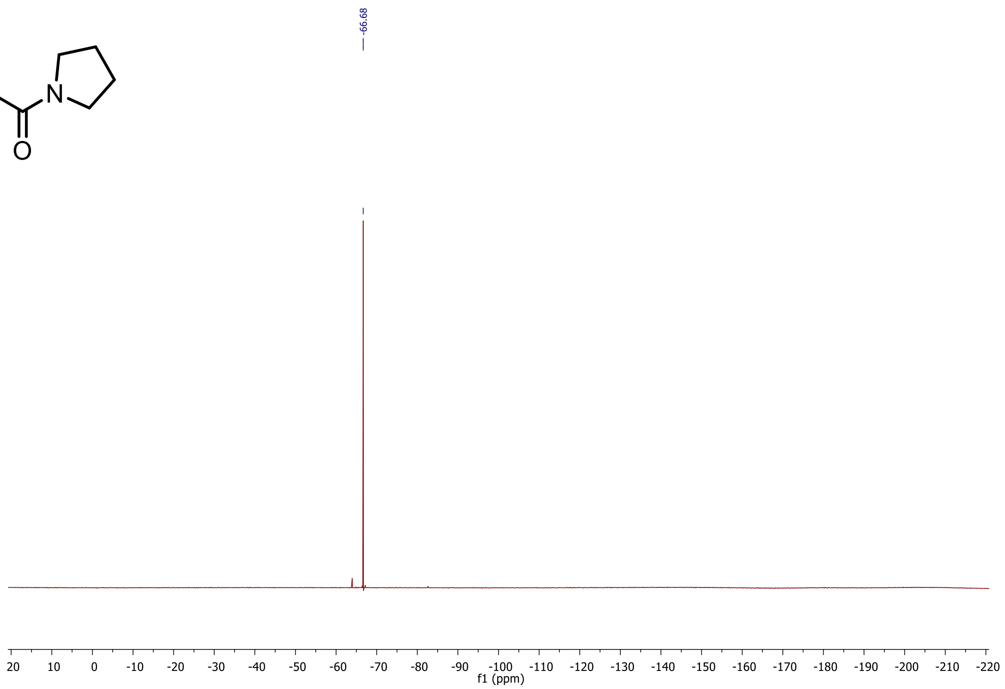

^{19}F
$CDCl_3$
376 MHz

r) (8R,9S,10S,13R,14S,17R)-10,13-Dimethyl-17-((R)-5-oxo-5-(pyrrolidin-1-yl)pentan-2-yl)dodecahydro-3H-cyclopenta[a]phenanthrene-3,7,12(2H,4H)-trione-2-D (2r)

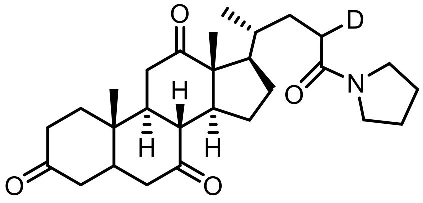

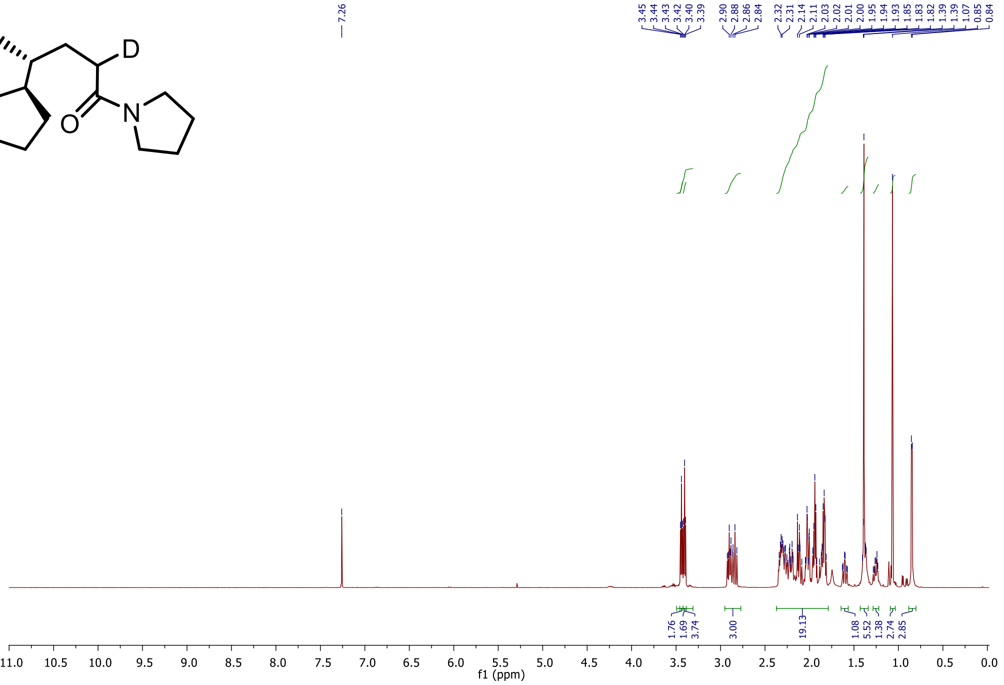

[1]H
$CDCl_3$
600 MHz

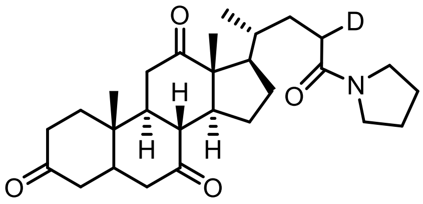

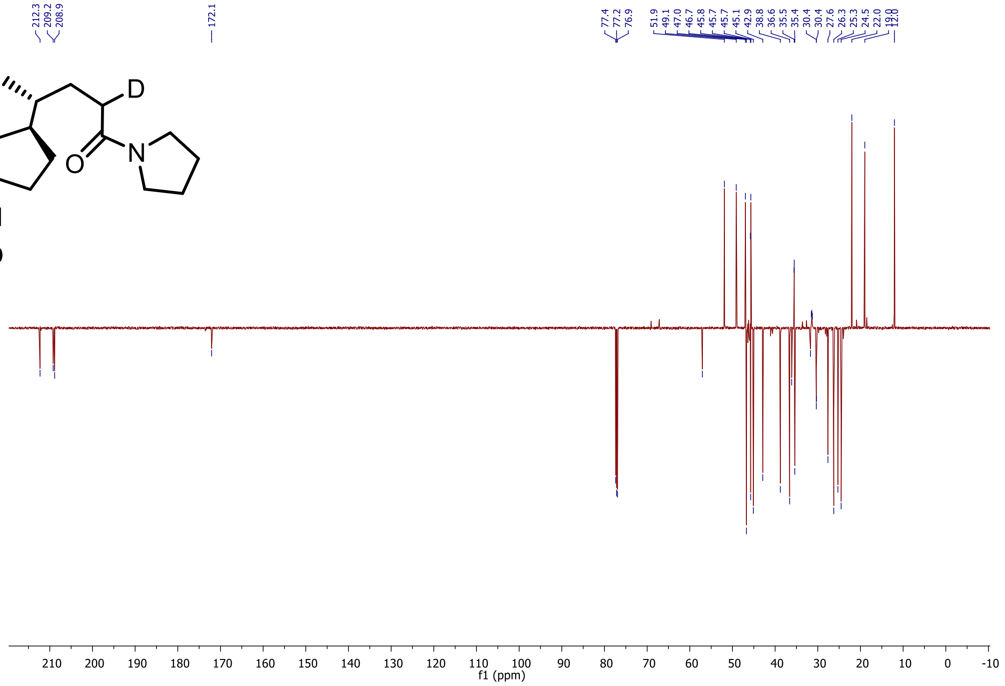

[13]C
$CDCl_3$
151 MHz

s) *N,N*-Dibenzylpentanamide-2-D_2 (7)

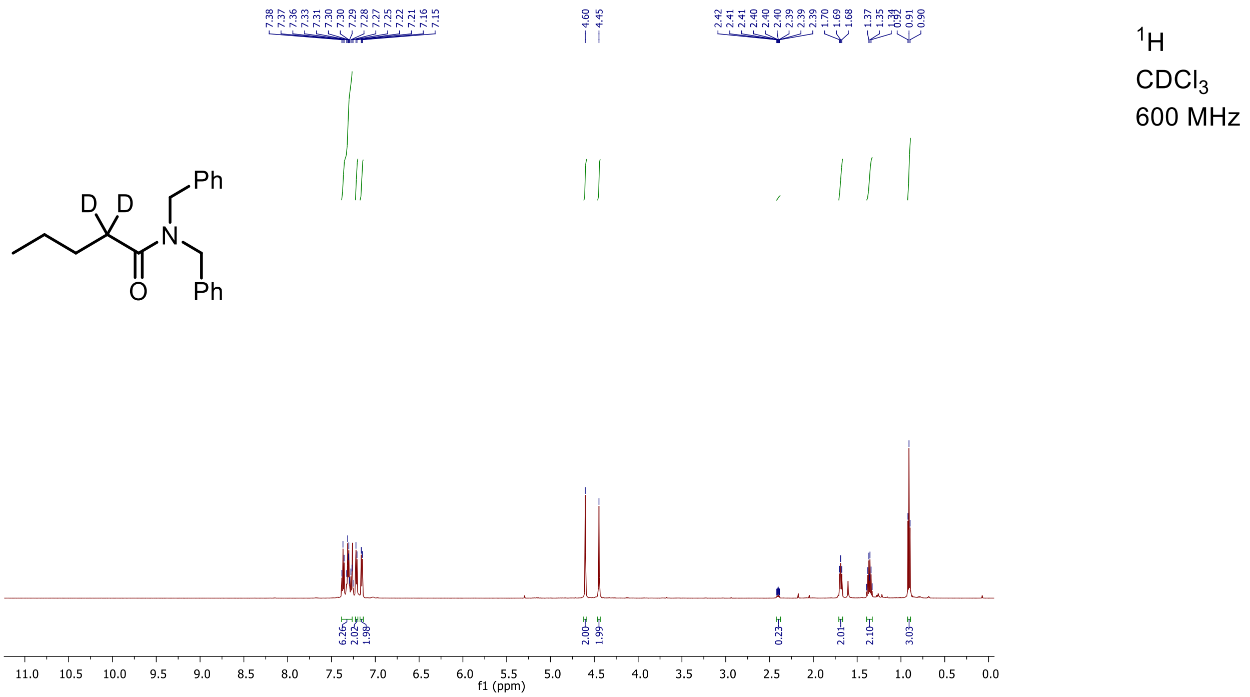

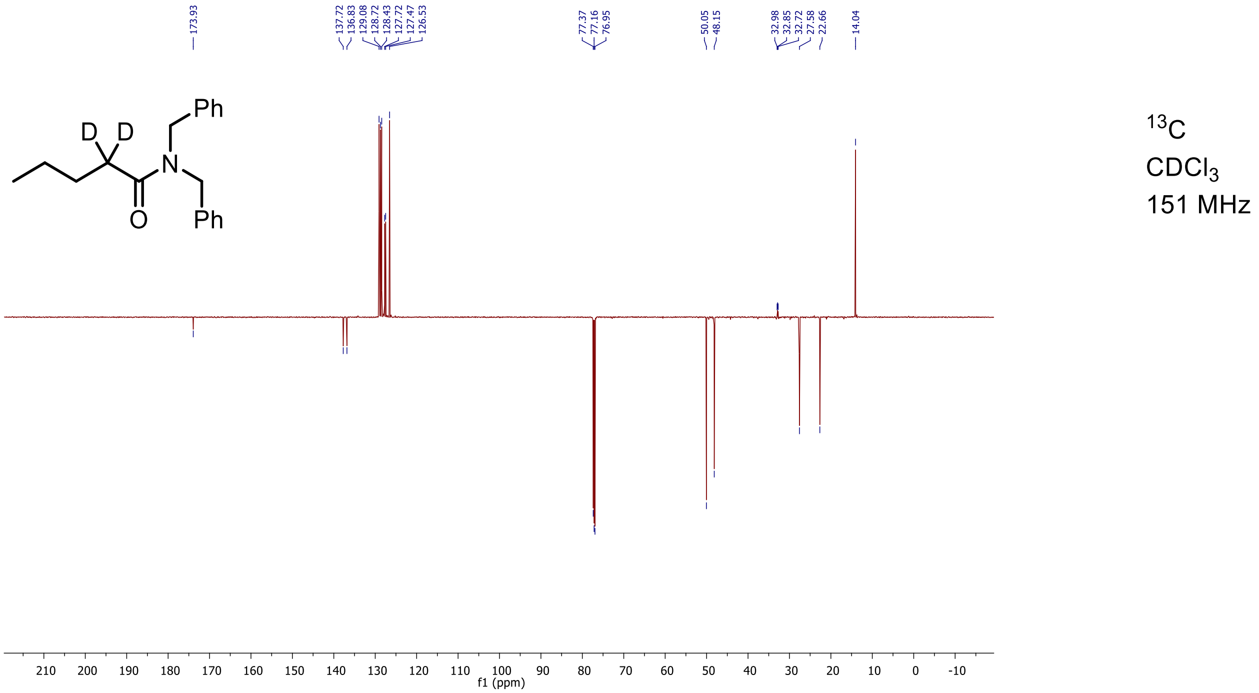

4.3. NMR of Specific Reactions and Mechanistic Studies

a) Dimethylsulfoxide ^{18}O (6)

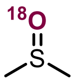

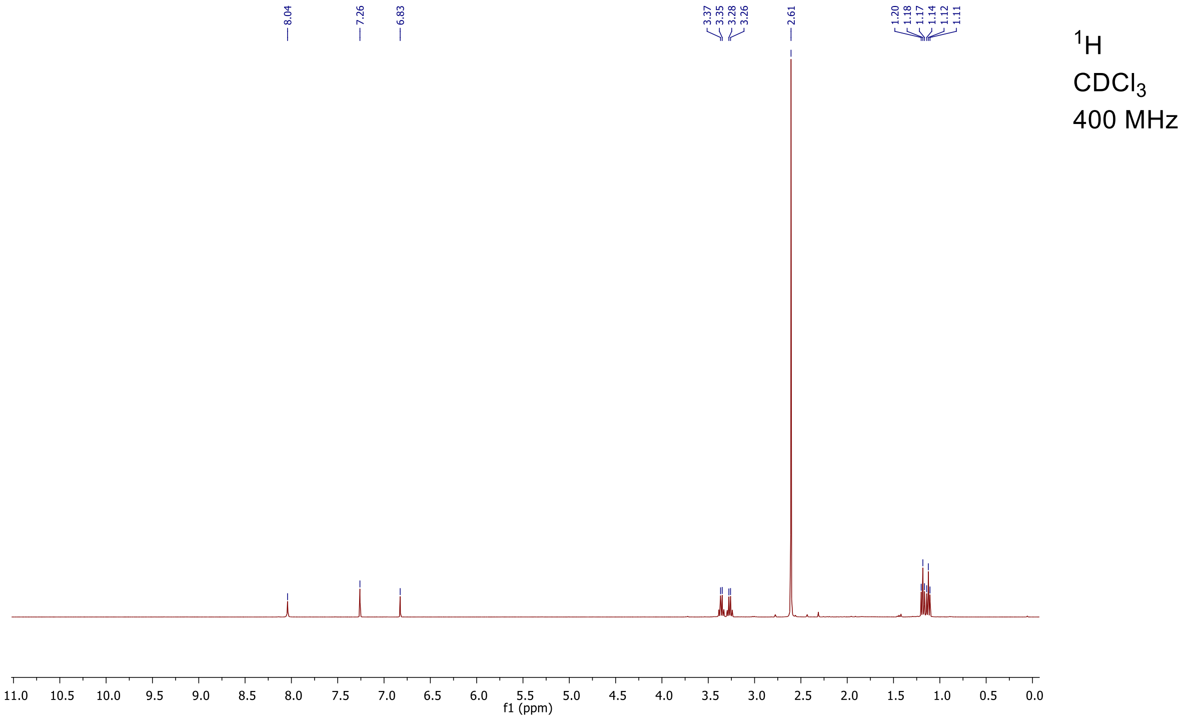

b) 4-Phenyl-1-(pyrrolidin-1-yl)butan-1-one-^{18}O (1a-[18O])

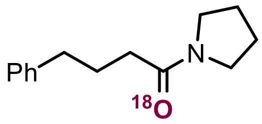

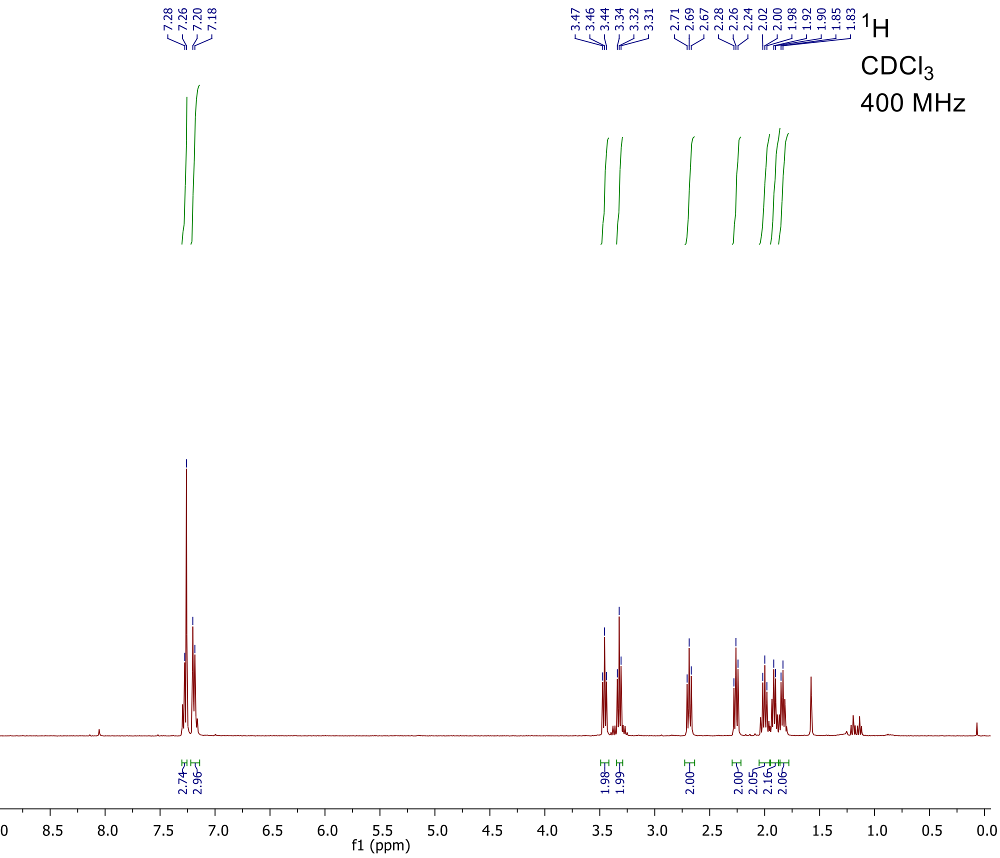

c) ***N,N*-Diethylnonanamide-2-D (2c)**

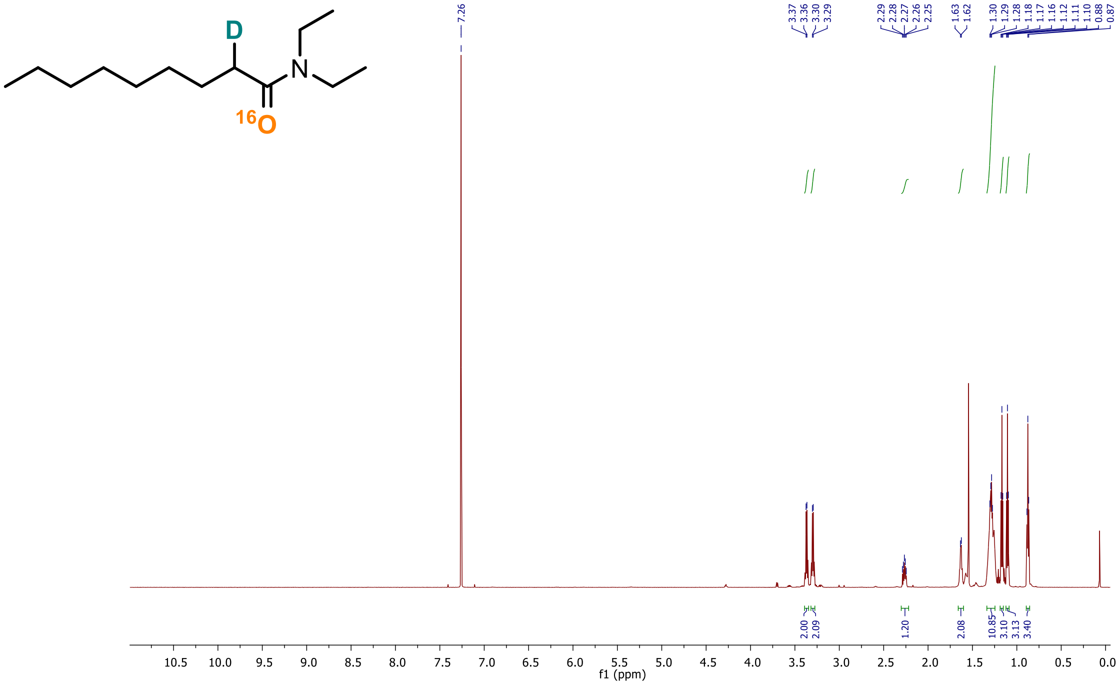

^1H
$CDCl_3$
700 MHz

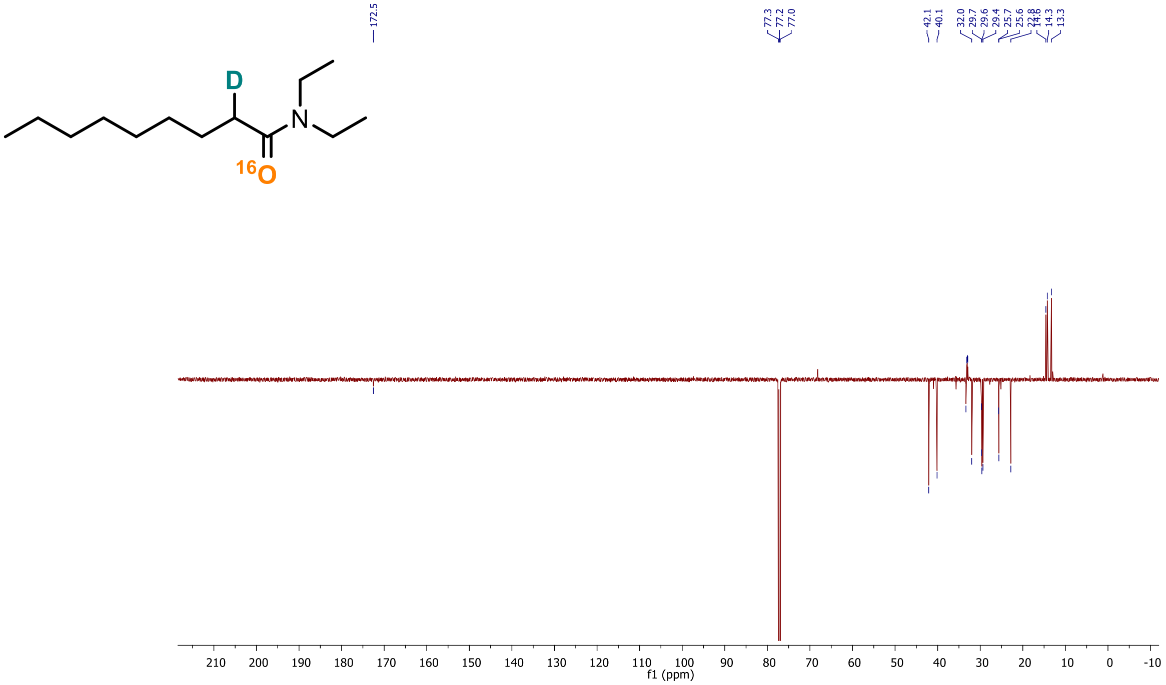

^1H
$CDCl_3$
176 MHz

5. Computational Details

The conformations analysis of all intermediates, products, and reactants were initially searched applying CREST[10] based on the GFN-xTB method[11] developed by S. Grimme The conformational space of transition states was analyzed with Macromodel[12] software The structures obtained at the stage of the conformational search have been re-optimized with Density functional theory (DFT) applying the PBE0 functional[13,14] and the def2-SVP basis set.[15] Frequencies have been calculated at the same level of theory as that used for geometry optimization to characterize the stationary points as either of minima (no imaginary frequencies) or first-order saddle points (one imaginary frequency). Thermal contributions to free energies were calculated from vibrational frequencies using the quasi-rigid rotor-harmonic oscillator (RRHO) approach of R. Paton at 298.15 K using *GoodVibes*.[16–19] Solvation effects were incorporated using the SMD[20] model with dichloromethane as the solvent and the free energies were corrected at the 0.1 M concentration using *GoodVibes*. Molecular structure visualizations were obtained using PyMol. DFT computations have been performed with the Gaussian 16, Revision A.03.[21]

Energy summary of the most stable conformers for each species.

Structure	E	ZPE	H	qh-H	T.S	T.qh-S	G(T)	qh-G(T)
**1**	-673.686212	0.306706	-673.363384	-673.366182	0.060581	0.057105	-673.423965	-673.423287
**dmso**	-552.634265	0.079385	-552.548361	-552.54844	0.033887	0.033894	-552.582248	-552.582334
**2-I-py**	-544.93424	0.078822	-544.848751	-544.848877	0.037476	0.037464	-544.886227	-544.886342
**A(E)**	-2110.785977	0.402948	-2110.353179	-2110.359283	0.092034	0.084646	-2110.445213	-2110.443929
**A(Z)**	-2110.789684	0.403009	-2110.35669	-2110.363136	0.09387	0.08557	-2110.450559	-2110.448706
**Pyridinium Triflate**	-1505.746348	0.12034	-1505.611207	-1505.613538	0.059132	0.056064	-1505.670339	-1505.669602
**Dimethyl sulfonium triflate**	-1437.161943	0.096862	-1437.051986	-1437.053831	0.052441	0.050465	-1437.104427	-1437.104296
**Tf2O**	-1845.236287	0.054582	-1845.16627	-1845.168554	0.058044	0.055519	-1845.224314	-1845.224074
**TS(E)**	-2110.772714	0.401257	-2110.341933	-2110.348277	0.093382	0.08479	-2110.435315	-2110.433067
**TS(Z)**	-2110.766464	0.401084	-2110.33596	-2110.342171	0.094379	0.085129	-2110.430339	-2110.4273

Cartesian coordinates of the most stable conformations computed at the wB97XD/def2-TZVP level of theory and SMD model solvation using dichloromethane as solvent

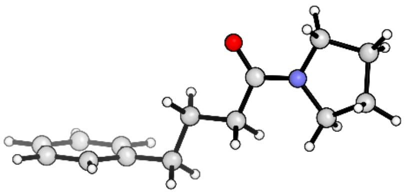

1

C	1.669051	-1.066247	-0.016105
C	3.080618	-0.541653	0.007424
C	3.766491	-0.267028	-1.183849
C	5.059654	0.255833	-1.164947
C	5.692975	0.516217	0.050932
C	5.022615	0.248275	1.245105
C	3.729537	-0.274640	1.220907
H	3.212630	-0.485230	2.162430
H	5.511253	0.445043	2.203509
H	6.707476	0.923426	0.067802
H	5.577457	0.458559	-2.106650
H	3.278653	-0.471471	-2.142081
H	1.502465	-1.708134	0.865441
H	1.531671	-1.707757	-0.902951
C	0.617909	0.044955	-0.033739
H	0.776746	0.694786	-0.909568
H	0.751044	0.695374	0.846290
C	-0.801346	-0.494606	-0.056000
C	-1.853135	0.597778	-0.087947
O	-1.560013	1.787443	-0.149345
N	-3.143531	0.190420	-0.045747
C	-3.620154	-1.185552	0.041752
C	-5.121800	-1.051450	-0.193038
H	-5.692973	-1.868484	0.270760
C	-5.440746	0.325098	0.383425
C	-4.234304	1.158303	-0.032035
H	-4.006075	1.985055	0.656994
H	-4.370869	1.601226	-1.035697

H	-6.389491	0.743883	0.018303
H	-5.498422	0.269921	1.483226
H	-5.333128	-1.065045	-1.275077
H	-3.133669	-1.831887	-0.704673
H	-3.408974	-1.613496	1.039228
H	-0.950265	-1.148693	-0.933977
H	-0.984898	-1.141251	0.820375

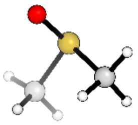

DMSO

S	-0.261794	0.420616	0.000000
O	1.108614	1.060923	0.000000
C	-0.261794	-0.772928	-1.356855
H	-1.198271	-1.350379	-1.355430
H	0.614149	-1.430581	-1.252456
H	-0.185216	-0.190095	-2.285859
C	-0.261794	-0.772928	1.356855
H	-0.185216	-0.190095	2.285859
H	0.614149	-1.430581	1.252456
H	-1.198271	-1.350379	1.355430

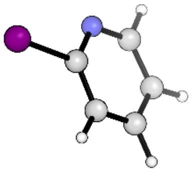

2-I-py

C	-0.025860	3.297445	-0.000000
C	-1.185574	2.527625	-0.000000
C	1.203749	2.639584	0.000000
N	-1.167861	1.191842	-0.000000
C	1.231383	1.247545	0.000000
C	0.000000	0.588783	-0.000000
H	-2.172500	3.002967	-0.000000
H	-0.086807	4.387716	-0.000000
H	2.140099	3.203848	0.000000
H	2.171260	0.693596	0.000000
I	-0.023004	-1.536546	-0.000000

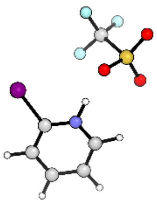

Pyridinium Triflate

C	-1.186258	3.316893	-0.018482
C	-2.515986	3.019190	0.280563
C	-2.951147	1.695597	0.303016
C	-2.035676	0.687404	0.022930
N	-0.764770	1.005682	-0.260475
C	-0.321548	2.272256	-0.288003
H	0.737734	2.385828	-0.537441
H	-0.034633	0.243950	-0.472722
I	-2.566741	-1.323197	0.024565
H	-3.986551	1.442795	0.533672
H	-3.224838	3.821835	0.498268
H	-0.821110	4.344217	-0.044092
O	1.132929	-0.722852	-0.777860
S	2.521627	-0.176782	-0.877301
O	3.451980	-1.045420	-1.588794
O	2.562345	1.257271	-1.196749
C	3.078210	-0.239231	0.885422
F	2.256340	0.478899	1.650201
F	3.084288	-1.486703	1.338592
F	4.302754	0.258353	1.008437

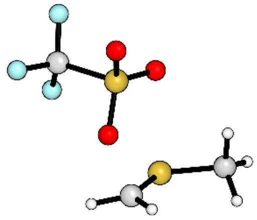

Dimethyl sulfonium triflate

S	-0.846861	0.844834	-0.286555
O	0.278127	1.040504	0.828103
O	-1.946387	1.728344	0.018799
O	-0.259390	0.772249	-1.609163
C	-1.424509	-0.858586	0.178549
F	-1.839914	-0.856014	1.427483
F	-2.421034	-1.180511	-0.623381
F	-0.443932	-1.728211	0.037381

S	2.659420	-0.296127	0.584445
C	1.685934	1.195027	0.487726
H	2.056566	1.883027	1.258883
H	1.773819	1.669779	-0.499549
C	2.592547	-0.927618	-1.109235
H	3.243797	-1.813942	-1.122943
H	2.987943	-0.188734	-1.821170
H	1.578222	-1.228534	-1.398960

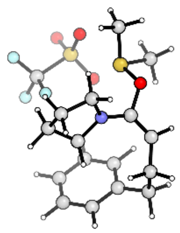

A_E

C	-0.853067	2.836780	-0.563225
C	-1.601338	1.958368	-1.347848
C	-2.972128	1.772069	-1.123308
C	-3.577847	2.507691	-0.094805
C	-2.837100	3.392704	0.689291
C	-1.469545	3.556258	0.460904
H	-0.884696	4.242726	1.079203
H	-3.331348	3.957771	1.484664
H	-4.648688	2.382394	0.094788
C	-3.763402	0.767591	-1.918168
H	-4.819004	1.080121	-1.952194
H	-3.403559	0.750813	-2.960570
C	-3.705277	-0.665753	-1.352668
C	-2.372588	-1.325645	-1.499318
C	-1.581232	-1.843024	-0.539083
N	-1.631174	-1.748498	0.820653
C	-1.396106	-2.897927	1.697397
C	-1.813078	-2.386264	3.075363
H	-1.239474	-2.858133	3.886149
C	-1.603010	-0.878117	2.972655
H	-2.149995	-0.307381	3.736993
C	-2.089816	-0.580902	1.561683
H	-1.669763	0.338441	1.127167
H	-3.191718	-0.482728	1.557866
H	-0.532735	-0.629339	3.059655
H	-2.879638	-2.602149	3.250507
H	-1.981818	-3.776542	1.377747
H	-0.333262	-3.196265	1.695952
O	-0.498253	-2.623898	-0.996209
S	0.990980	-1.874373	-0.806242
C	1.407872	-1.770358	-2.539686
H	1.181049	-2.737026	-3.008783
H	0.803299	-0.960031	-2.965628
H	2.472292	-1.518048	-2.605413
C	1.919569	-3.312753	-0.306731
H	1.732480	-3.478751	0.762059
H	1.584190	-4.168158	-0.908529
H	2.979777	-3.086234	-0.470676
H	-1.985587	-1.420637	-2.519220
H	-4.445620	-1.266729	-1.911404
H	-4.041940	-0.666623	-0.306146
H	-1.104794	1.402304	-2.147435
H	0.218376	2.937413	-0.751381
S	3.050622	0.760767	-0.550802
O	3.196667	-0.717641	-0.406261
O	4.314082	1.480359	-0.690957
O	1.956313	1.147592	-1.451887
C	2.445025	1.260179	1.126761
F	1.296617	0.652316	1.418731
F	3.332250	0.938733	2.061990
F	2.243657	2.573760	1.178167

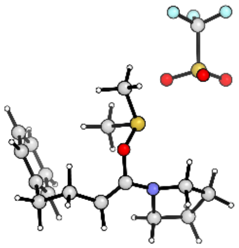

A_Z

C	-4.009885	-1.499135	2.289652
C	-3.789510	-2.831072	1.937090
C	-4.006080	-3.236684	0.618980
C	-4.432696	-2.314412	-0.336694
C	-4.653833	-0.971125	0.000586
C	-4.438275	-0.580420	1.329582
H	-4.620781	0.458668	1.619048
C	-5.074832	0.021672	-1.049934
H	-5.668333	0.825477	-0.583863
H	-5.734791	-0.478972	-1.776226
C	-3.901532	0.649918	-1.824155

C	-3.059322	1.579278	-1.010744
C	-1.722664	1.526914	-0.868990
N	-0.934282	2.250514	-0.007540
C	-1.524326	2.978810	1.104708
C	-0.336048	3.707678	1.759506
H	-0.537849	4.787823	1.807295
C	0.872976	3.409618	0.858737
H	1.471500	2.577821	1.261090
H	1.543972	4.272111	0.739382
C	0.250068	2.978389	-0.459300
H	-0.039603	3.848928	-1.081632
H	0.924739	2.344494	-1.053309
H	-0.166778	3.364741	2.790074
H	-2.040480	2.275675	1.777069
H	-2.276268	3.710246	0.751491
O	-1.009514	0.606880	-1.634281
S	0.217435	-0.182216	-0.797997
C	0.920878	-0.918645	-2.258432
H	1.663339	-1.645566	-1.909744
H	1.417322	-0.112125	-2.812673
H	0.122424	-1.389044	-2.846944
C	-0.706021	-1.530743	-0.084446
H	-1.240127	-2.076806	-0.872200
H	-1.400886	-1.088778	0.643973
H	0.025060	-2.164726	0.433946
O	2.218646	-1.206683	0.417810
S	3.271019	-0.150871	0.385854
O	3.547096	0.481128	1.676659
O	3.168113	0.744070	-0.777363
C	4.791255	-1.144902	0.036278
F	4.660719	-1.808325	-1.110481
F	5.012745	-2.027453	1.004109
F	5.854230	-0.354015	-0.060099
H	-3.569259	2.400099	-0.497800
H	-3.277914	-0.142676	-2.265449
H	-4.331769	1.211495	-2.673399
H	-4.605453	-2.643168	-1.366437
H	-3.847213	-4.280595	0.334330
H	-3.458107	-3.552531	2.688665
H	-3.852918	-1.172653	3.321476

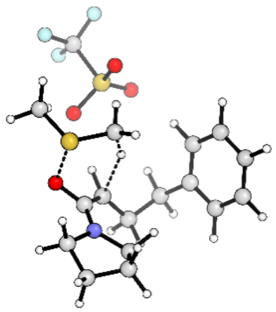

TS(E)

C	2.667642	3.953384	0.844787
C	2.333421	3.392923	-0.387383
C	1.081392	2.793591	-0.592934
C	0.168153	2.781671	0.469790
C	0.500840	3.341573	1.705501
C	1.751904	3.926188	1.898996
H	2.011408	4.367692	2.865000
H	-0.229131	3.323472	2.519639
H	-0.821513	2.339605	0.322940
C	0.739210	2.167984	-1.921500
H	-0.342572	1.967860	-1.965399
H	0.968874	2.879957	-2.730286
C	1.508610	0.866045	-2.216764
C	1.056154	-0.280594	-1.394266
C	1.779855	-1.333223	-0.828977
N	3.097358	-1.385223	-0.519050
C	3.781614	-2.660990	-0.275623
C	5.256699	-2.274161	-0.275282
H	5.861857	-2.944692	0.351273
C	5.237676	-0.830256	0.216011
H	6.157803	-0.274168	-0.013261
C	4.021496	-0.258221	-0.500203
H	4.296618	0.059161	-1.522680
H	3.573847	0.606284	0.014120
H	5.082853	-0.798428	1.307176
H	5.659510	-2.315966	-1.300417
H	3.526473	-3.397868	-1.053345
H	3.485700	-3.089240	0.696149
O	1.050634	-2.371473	-0.507963
S	0.622411	-2.379263	1.480354
C	-1.005721	-3.021290	1.135621
H	-0.865647	-4.029751	0.721885
H	-1.551027	-3.082567	2.089701
H	-1.522243	-2.359391	0.422806

C	0.387402	-0.655868	1.665867
H	1.050833	-0.286205	2.459186
H	0.694451	-0.166715	0.691068
H	-0.672748	-0.401010	1.826586
H	-0.027955	-0.445144	-1.347698
H	2.592021	1.033984	-2.162893
H	1.292996	0.592934	-3.269147
H	3.055594	3.427329	-1.209361
H	3.646938	4.420488	0.982010
S	-2.918025	0.226197	-0.128150
O	-2.663892	0.195678	1.324342
O	-2.986334	1.562989	-0.732706
O	-2.155817	-0.773903	-0.907364
C	-4.652272	-0.402162	-0.257678
F	-5.044038	-0.448150	-1.527061
F	-4.745111	-1.629538	0.249555
F	-5.490607	0.385697	0.408257

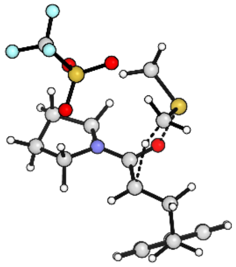

TS(Z)

C	5.564180	1.716173	-0.199661
C	4.832404	0.998672	-1.147248
C	4.756324	-0.399235	-1.092255
C	5.435000	-1.060611	-0.059116
C	6.167430	-0.348084	0.889507
C	6.233758	1.044624	0.823116
H	6.809348	1.604378	1.565163
H	6.693128	-0.883856	1.684705
H	5.392238	-2.152763	-0.001045
C	3.942301	-1.173543	-2.094400
H	3.822328	-0.580349	-3.014728
H	4.478028	-2.094511	-2.374851
C	2.544929	-1.596526	-1.582647
C	1.662978	-0.433106	-1.308232
C	1.349451	0.027364	-0.026161
N	0.617590	1.140272	0.221261
C	0.611554	1.806817	1.519897
C	-0.428423	2.901779	1.332481
H	-0.293569	3.732224	2.040399
C	-0.236170	3.306727	-0.125305
H	-1.109385	3.819428	-0.553555
C	0.052974	1.982984	-0.831389
H	0.763584	2.117525	-1.663700
H	-0.863186	1.515153	-1.230699
H	0.629910	3.982062	-0.220935
H	-1.434764	2.476794	1.469380
H	0.359144	1.114270	2.333371
H	1.611361	2.227276	1.742920
O	1.725710	-0.711858	0.992571
S	0.435992	-2.184400	1.442760
C	-0.792971	-1.314785	2.398285
H	-0.274765	-0.881550	3.265008
H	-1.321928	-0.554048	1.805724
H	-1.508388	-2.076004	2.749775
C	-0.298872	-2.405292	-0.119874
H	-1.397580	-2.294122	-0.077919
H	0.097162	-1.580769	-0.800882
H	0.028519	-3.367705	-0.535279
H	1.387757	0.190086	-2.164174
H	2.662918	-2.218391	-0.683255
H	2.087084	-2.220202	-2.369640
H	4.315586	1.532914	-1.950356
H	5.613680	2.806683	-0.263759
S	-3.391724	-0.210559	-0.240594
O	-3.348249	-1.652355	0.080290
O	-2.998008	0.671418	0.873872
O	-2.851347	0.150151	-1.559461
C	-5.203540	0.117020	-0.414662
F	-5.723057	-0.614574	-1.396461
F	-5.848727	-0.180315	0.709851
F	-5.424253	1.399136	-0.691984

6. References

[1] T. E. Stang, P. J.; Dueber, *Org. Synth.* **1974**, *54*, 79.

[2] P. Adler, C. J. Teskey, D. Kaiser, M. Holy, H. H. Sitte, N. Maulide, *Nat. Chem.* **2019**, *11*, 329–334.

[3] C. R. Gonçalves, M. Lemmerer, C. J. Teskey, P. Adler, D. Kaiser, B. Maryasin, L. González, N. Maulide, *J. Am. Chem. Soc.* **2019**, *141*, 18437–18443.

[4] A. De La Torre, D. Kaiser, N. Maulide, *J. Am. Chem. Soc.* **2017**, *139*, 6578–6581.

[5] C. J. Teskey, P. Adler, C. R. Gonçalves, N. Maulide, *Angew. Chem. Int. Ed.* **2019**, *58*, 447–451.

[6] D. Kaiser, C. J. Teskey, P. Adler, N. Maulide, *J. Am. Chem. Soc.* **2017**, *139*, 16040–16043.

[7] J. Bai, B. K. Zambroń, P. Vogel, *Org. Lett.* **2014**, *16*, 604–607.

[8] D. Doskočilová, J. Dybal, P. Schmidt, B. Schneider, J. Kříž, *J. Mol. Struct.* **1995**, *350*, 9–18.

[9] A. H. Fenselau, J. G. Moffatt, *J. Am. Chem. Soc.* **1966**, *88*, 1762–1765.

[10] P. Pracht, F. Bohle, S. Grimme, *Phys. Chem. Chem. Phys.* **2020**.

[11] S. Grimme, C. Bannwarth, P. Shushkov, *J. Chem. Theory Comput.* **2017**, *13*, 1989–2009.

[12] N. Schrödinger, LLC, New York, **2020**.

[13] J. P. Perdew, M. Ernzerhof, K. Burke, *J. Chem. Phys.* **1996**, *105*, 9982–9985.

[14] C. Adamo, V. Barone, *J. Chem. Phys.* **1999**, *110*, 6158–6170.

[15] F. Weigend, R. Ahlrichs, *Phys. Chem. Chem. Phys.* **2005**, *7*, 3297.

[16] G. Luchini, J. V. Alegre-Requena, Y. Guan, I. Funes-Ardoiz, R. S. Paton, **2019**, DOI 10.5281/zenodo.595246.

[17] I. M. Alecu, J. Zheng, Y. Zhao, D. G. Truhlar, *J. Chem. Theory Comput.* **2010**, *6*, 2872–2887.

[18] S. Grimme, *Chem. Eur. J.* **2012**, *18*, 9955–9964.

[19] Y.-P. Li, J. Gomes, S. Mallikarjun Sharada, A. T. Bell, M. Head-Gordon, *J. Phys. Chem. C* **2015**, *119*, 1840–1850.

[20] A. V. Marenich, C. J. Cramer, D. G. Truhlar, *J. Phys. Chem. B* **2009**, *113*, 6378–6396.

[21] G. E. S. M. J. Frisch, G. W. Trucks, H. B. Schlegel, V. B. M. A. Robb, J. R. Cheeseman, G. Scalmani, A. V. M. G. A. Petersson, H. Nakatsuji, X. Li, M. Caricato, H. P. H. J. Bloino, B. G.

Janesko, R. Gomperts, B. Mennucci, D. W.-Y. J. V. Ortiz, A. F. Izmaylov, J. L. Sonnenberg, A. P. F. Ding, F. Lipparini, F. Egidi, J. Goings, B. Peng, N. R. T. Henderson, D. Ranasinghe, V. G. Zakrzewski, J. Gao, R. F. G. Zheng, W. Liang, M. Hada, M. Ehara, K. Toyota, H. N. J. Hasegawa, M. Ishida, T. Nakajima, Y. Honda, O. Kitao, J. E. P. T. Vreven, K. Throssell, J. A. Montgomery, Jr., K. N. K. F. Ogliaro, M. J. Bearpark, J. J. Heyd, E. N. Brothers, J. N. V. N. Staroverov, T. A. Keith, R. Kobayashi, S. S. I. K. Raghavachari, A. P. Rendell, J. C. Burant, R. C. J. Tomasi, M. Cossi, J. M. Millam, M. Klene, C. Adamo, O. F. J. W. Ochterski, R. L. Martin, K. Morokuma, and D. J. F. J. B. Foresman, *Gaussian, Inc., Wallingford, CT* **2016**.
